# New evidence from high-resolution computed microtomography of Triassic stem-mammal skulls from South America enhances discussions on turbinates before the origin of Mammaliaformes

**DOI:** 10.1038/s41598-024-64434-5

**Published:** 2024-06-15

**Authors:** Pedro H. M. Fonseca, Agustín G. Martinelli, Pamela G. Gill, Emily J. Rayfield, Cesar L. Schultz, Leonardo Kerber, Ana Maria Ribeiro, Heitor Francischini, Marina B. Soares

**Affiliations:** 1 Programa de Pós-Graduação em Geociências, Instituto de Geociências, Universidade Federal do Rio Grande do Sul, Av. Bento Gonçalves, 9500, Bairro Agronomia, Porto Alegre, Rio Grande do Sul 91501-970 Brazil; 2 CONICET-Sección Paleontología de Vertebrados, Museo Argentino de Ciencias Naturales “Bernardino Rivadavia”, Av. Ángel Gallardo 470, C1405DJR Buenos Aires, CABA Argentina; 3 Núcleo Milenio EVOTEM-Evolutionary Transitions of Early Mammals-ANID, Santiago, Chile; 4 Palaeobiology Research Group, School of Earth Sciences, University of Bristol, Life Sciences Building, Bristol, BS8 1TQ UK; 5 Science Department, Natural History Museum, Cromwell Road, London, SW7 5HD, UK; 6 Centro de Apoio à Pesquisa Paleontológica, Universidade Federal de Santa Maria, São João do Polêsine, Brazil; 7 Museu de Ciências Naturais/SEMA, Porto Algre, RS Brazil; 8 Departamento de Geologia e Paleontologia, Museu Nacional, Universidade Federal do Rio de Janeiro, Quinta da Boa Vista s/n, São Cristovão, Rio de Janeiro, RJ 20940-040 Brazil

**Keywords:** Evolution, Palaeontology

## Abstract

The nasal cavity of living mammals is a unique structural complex among tetrapods, acquired along a series of major morphological transformations that occurred mainly during the Mesozoic Era, within the Synapsida clade. Particularly, non-mammaliaform cynodonts document several morphological changes in the skull, during the Triassic Period, that represent the first steps of the mammalian bauplan. We here explore the nasal cavity of five cynodont taxa, namely *Thrinaxodon*, *Chiniquodon*, *Prozostrodon*, *Riograndia*, and *Brasilodon*, in order to discuss the main changes within this skull region. We did not identify ossified turbinals in the nasal cavity of these taxa and if present, as non-ossified structures, they would not necessarily be associated with temperature control or the development of endothermy. We do, however, notice a complexification of the cartilage anchoring structures that divide the nasal cavity and separate it from the brain region in these forerunners of mammals.

## Introduction

Synapsida is a clade that diverged from basal amniotes at about 320 Ma, during the end of the Carboniferous Period, and includes the mammals as the crown group^1–3^. Its evolutionary history relates the morphological changes from the traditionally and erroneously called “mammal-like reptiles” (such as “pelycosaurs”, anomodonts, therocephalians, cynodonts) to the bauplan of mammals, a successful lineage with more than 6600 living species^4^. The excellent fossil record of synapsids has enabled researchers to address hypotheses about the origin of several physiological traits that now characterize mammals, such as the presence of hair, lactation, an evolved neocortex in the brain, thermoregulation, elevated aerobic capacity, truncated growth, dental diphyodonty, among others^1,5–15^. Particularly, the progressive increase of sustained aerobic capacity is evident along the synapsid lineage, markedly in more derived groups, such as cynodonts^8,16–18^. With a raise in aerobic activity there is a gradual increase in lung ventilation and, consequently, the loss of water and heat through respiration^19^. To control this, living mammals have structures inside the nasal cavity, named turbinals^20–23^. The turbinals and nasal septum are structures completely ossified in the snout of current mammals, which occurs during their perinatal stage from the ossification of the cartilages of the nasal capsule^24–26^. In addition to these physiological functions, the turbinals, together with other bones, like the presphenoid and mesethmoid^24^, provide the separation of the nasal cavity from the brain cavity by the cribriform plate.

If turbinals were present in extinct groups of synapsids, such as “pelycosaurs”, therocephalians, non-mammaliaform cynodonts^19,27–39^, they remained cartilaginous^19^ (but see^40^); therefore, they are hardly preserved in the fossil record. Despite this, other structures can prove indirectly the presence of these cartilaginous tissues inside the nasal cavity of non-mammaliaform synapsids^19,28,29,31,33^. Recently, Laaß and Kaestner^40^ described the presence of ossified maxilloturbinals for the fossorial anomodont dicynodont *Kawingasaurus fossilis* (late Permian of Tanzania). As a conclusion, the authors claimed the presence of endothermy in Permian cistecephalid dicynodonts^40^. In non-mammaliaform cynodonts (e.g., *Bolotridon*, *Massetognathus*, *Probainognathus*, *Pseudotherium*, *Brasilodon*) ridges located in the lateral and dorsal surfaces of the nasal cavity were identified as attachment areas of the cartilaginous turbinals and nasal septum, respectively^29,31,33,38,39^. In addition, some structures inside the nasal cavity were identified as probable fragments of ossified turbinal (i.e., nasoturbinal, first ethmoid, and mesethmoid) in the non-mammaliaform probainognathian *Brasilodon quadrangularis*^29^. However, Crompton et al.^31^ pointed out that these elements were misidentified. Subsequently, a similar structure inside the nasal cavity, likely a maxilloturbinal, was described for *Pseudotherium argentinus*^33^.

The presence of the turbinals in extant mammals and of structures provisionally associated with the turbinals (the ridges on the wall of the nasal cavity) in non-mammaliaform synapsids is directly associated with an endothermic condition by some authors^8,19,29,31,34,38,40^. In this sense, we used here as definition of endothermy as the continuous generation of body heat through metabolic processes, maintaining a relatively constant internal temperature independently of external conditions (i.e., tachymetabolic endothermy sensu^41^), excluding short-term torpor, aestivation, and hibernation phases^42^ (for discussions on termoregulatory regimens and terminology in extant and extinct taxa, see also, for example, Griggs et al.^41^, Clarke and Pörtner^43^, Lovegrove^44^, and Legendre and Davesne^45^). The origin of physiological traits related to endothermy along the synapsid clade is a hallmark event usually linked to other mammalian traits (e.g., high aerobic capacity, bigger brains, nocturnal lifestyle, parental care)^17,39,46–54^. In the search for the origin of endothermy, other proxies were investigated in the fossil record, such as the size of the nutrient foramina of the femur, morphology of semicircular canals of the inner ear, cementum annuli count in postcanine teeth, shift of the sprawling to erect posture, and closure of the pineal foramen, and indirect evidence of the presence of fur and vibrissae^13,15,18,31,42,46,55–63^. However, recent compelling work indicates that turbinals in current mammals do not have a direct relationship with endothermy, but are rather environmental, phylogenetic, or morphological responses^64^.

In addition to the presence of turbinals, other changes in the nasal cavity contributed to the evolutionary success of the non-mammaliaform cynodonts and mammals, such as modifications in the ventral region of the nasal cavity^31^. The main transformation is the development of the secondary bony palate, separating the nasal cavity from the oral cavity, and its posterior expansion^65,66^. In early diverging cynodonts, such as *Procynosuchus* and *Galesaurus*, the maxilla and palatine present a medial projection in the palatal region, but do not contact in the medial line to produce a closed secondary bony palate^27,67^. In cynognathians and some early diverging probainognathians, there is complete closure of the secondary bony palate^2^, but it remains relatively short compared to more crowned probainognathians, such as *Chiniquodon*, *Aleodon*, *Probainognathus*, *Prozostrodon*, *Riograndia*, *Brasilodon* and mammaliaforms^2,15,68–74^. In mammals, the ossification of the nasal capsule along with the participation of the palatine and vomer, forms the primary palate and separates the olfactory region from the respiratory region of the nasal cavity through the nasopharyngeal duct (Macrini^26^ and reference herein). The beginning of this mammalian configuration can be traced back to *Thrinaxodon*, in which due to the closure of the secondary bony palate, the structures that formerly composed the primary palate become part of the roof of the nasopharyngeal duct. This duct, formed by the vomer and palatine, sections the nasal cavity, allowing an improvement in olfactory capacities in its ventrodorsal region, and respiratory ability in its rostral portion^31^. Thus, the vomer, which anteriorly formed the primary palate in the early synapsids, divides the ventral region of the nasal cavity and serves as an anchorage for the other cartilaginous structures that divide the rest of the cavity in epicynodonts^8,15,19,29,31^. However, despite our knowledge of the evolution of the nasal cavity, little is known about the cartilaginous structures and the process of ossification of the nasal capsule, since this is not readily preserved in the fossil record^8,19,75^. In mammals, part of the nasal turbinals participated in the formation of the ventral region of the nasal cavity and other adjacent structures, such as the maxillary recess and sinus^26^.

**Figure 1:**
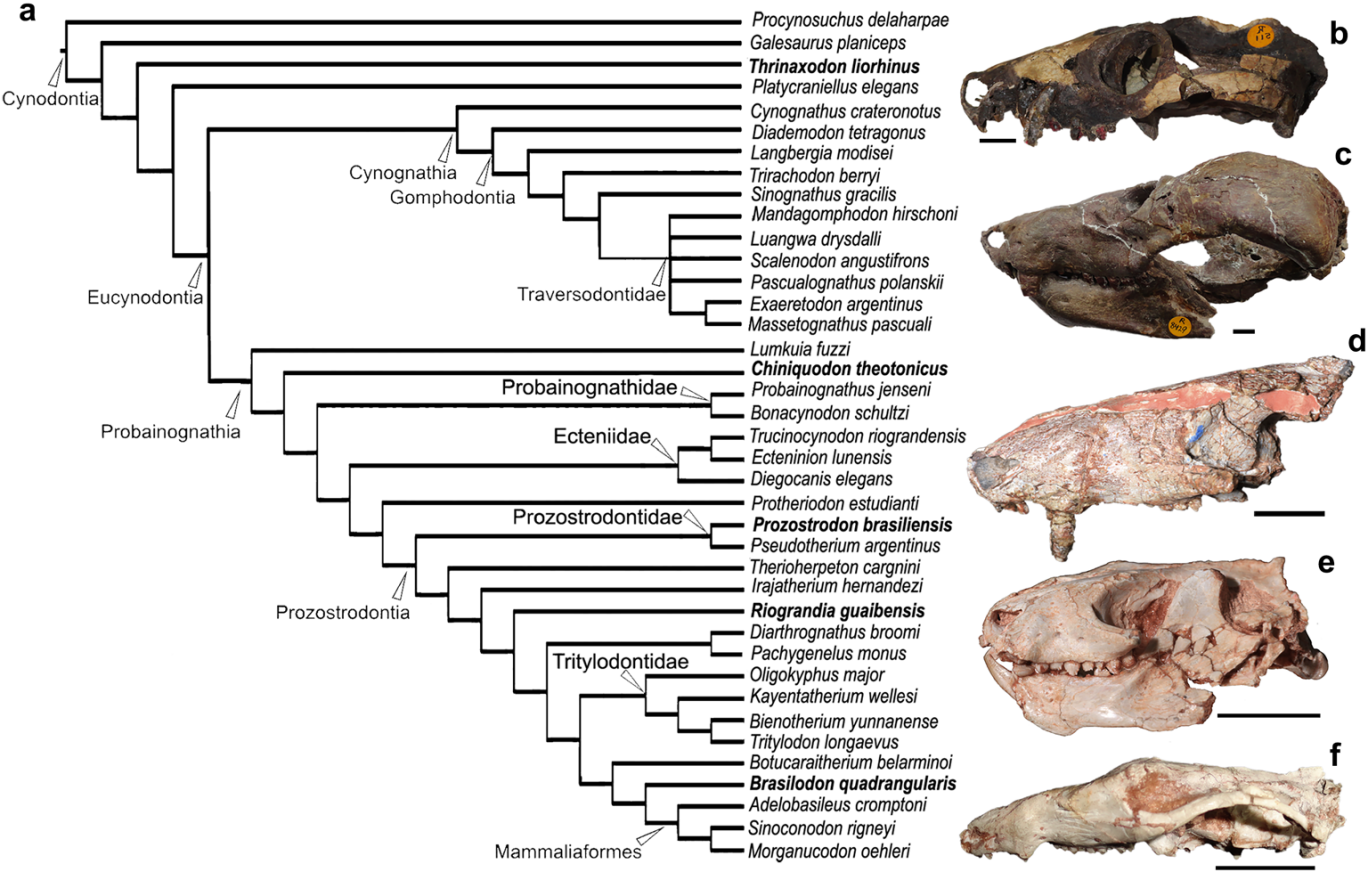
Phylogeny of non-mammaliaform cynodonts and earliest mammaliaforms. (**a**) Phylogeny of the Cynodontia (modified from Kerber et al.^73^). (**b–f**) Selected specimens of the species here analysed, in lateral view, and their phylogenetic placement, *Thrinaxodon liorhinus* (NHMUK PV R511, inverted) (**b**), *Chiniquodon theotonicus* (NHMUK PV R8429) (**c**), *Prozostrodon brasiliensis* (UFRGS-PV-248-T) (**d**), *Riograndia guaibensis* (UFRGS-PV-596-T, inverted) (**e**), *Brasilodon quadrangularis* (UFRGS-PV-1043-T) (**f**). Scale bar: 1 cm.

Although descriptions of the nasal cavity have already been made for several non-mammaliaform cynodonts^19,27,29,31,34,39,73,75–80^, improvement in the resolution and acquisition of tomographic images, as well as the advancement of techniques and analysis of these images, allows an increase in knowledge about the origin and evolution of the nasal cavity in the lineage of cynodonts, in which mammals are included. Thus, the aim of this contribution is to describe the cranial elements of the rostrum that encompass the nasal cavity, as well as its inner structures in five different species of cynodonts using µCT-scan images: the earliest epicynodont *Thrinaxodon liorhinus*, and the non-mammaliaform probainognathians *Chiniquodon theotonicus*, *Riograndia guaibensis*, *Prozostrodon brasiliensis*, and *Brasilodon quadrangularis* (Fig. 1), using as comparison the early mammaliaform *Morganucodon watsoni*^81^ and the current marsupial *Didelphis virginiana*^26,82^. Furthermore, through comparisons with different ontogenetic stages of *Brasilodon*, we can also evaluate variations at different growth stages. Finally, we provide new data about the discussions on the presence or absence of cartilaginous structures correlated with turbinals in non-mammaliaform cynodonts and discuss the physiological implications of this.

## Results

### General remarks of the rostrum

**Figure 2:**
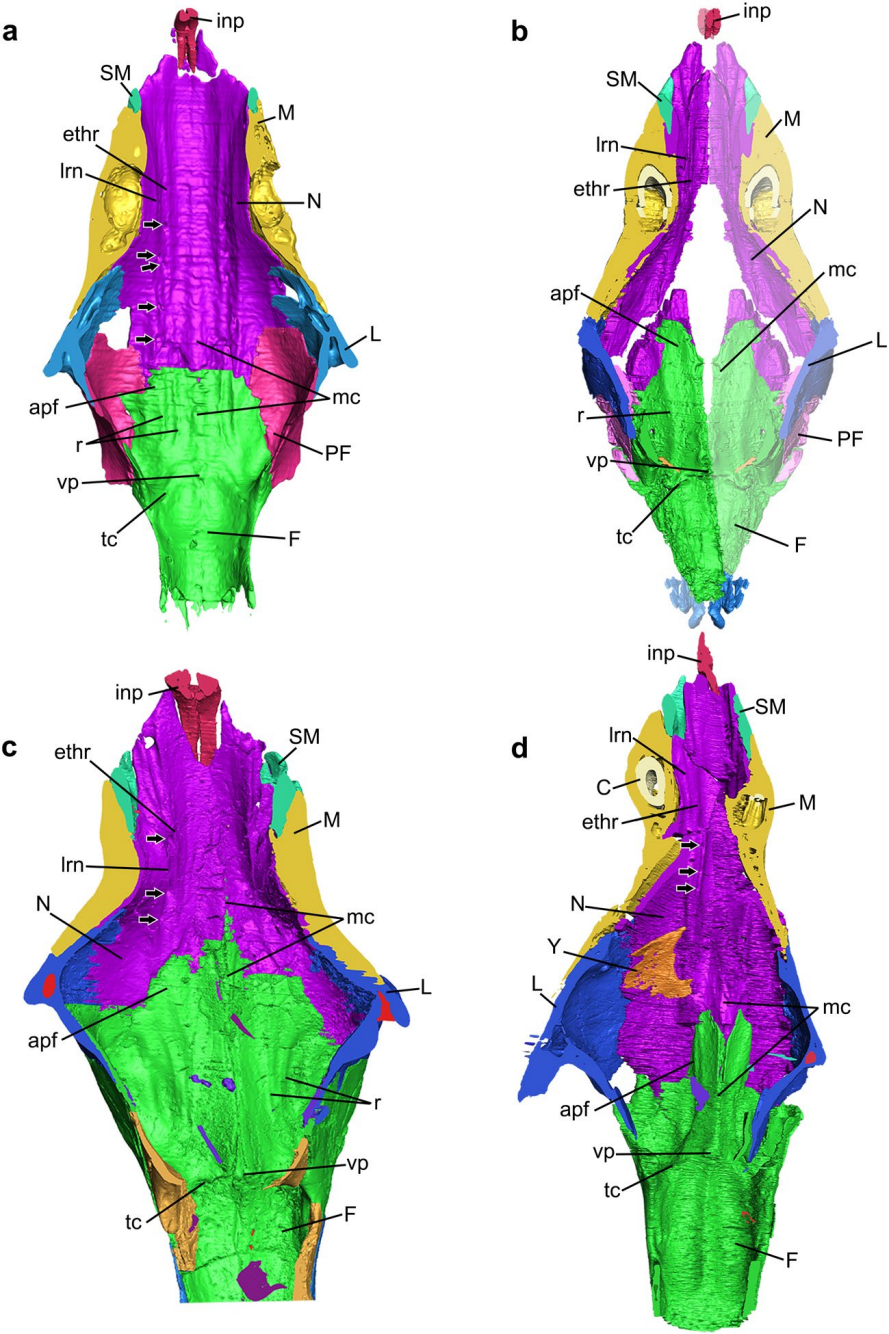
Nasal cavities of the studied cynodonts. (**a–d**) Skull section in ventral (internal) view, exhibiting the ventral region of the roof of the nasal cavity, in *Thrinaxodon liorhinus* (NHMUK PV R511) (**a**), *Prozostrodon brasiliensis* (UFRGS-PV-248-T; the left half of skull is mirrored) (**b**), *Riograndia guaibensis* (UFRGS-PV-596-T) (**c**), and *Brasilodon quadrangularis* (UFRGS-PV-1043-T) (**d**). Black arrows indicate visible foramina. Lighter region in (**b**) indicates mirroring. apf, anterior projection of the frontal; C, canine; F, frontal; ethr, ethmoid ridge of the nasal; inp, internarial process; L, lacrimal; lrn, lateral ridge of the nasal; M, maxilla; mc, median crest; N, nasal; PF, prefrontal; r, ridge; SM, Septomaxilla; tc, transverse crest; vp, ventral projection; Y, structure identified as a turbinal by Ruf et al.^29^.

**Figure 3:**
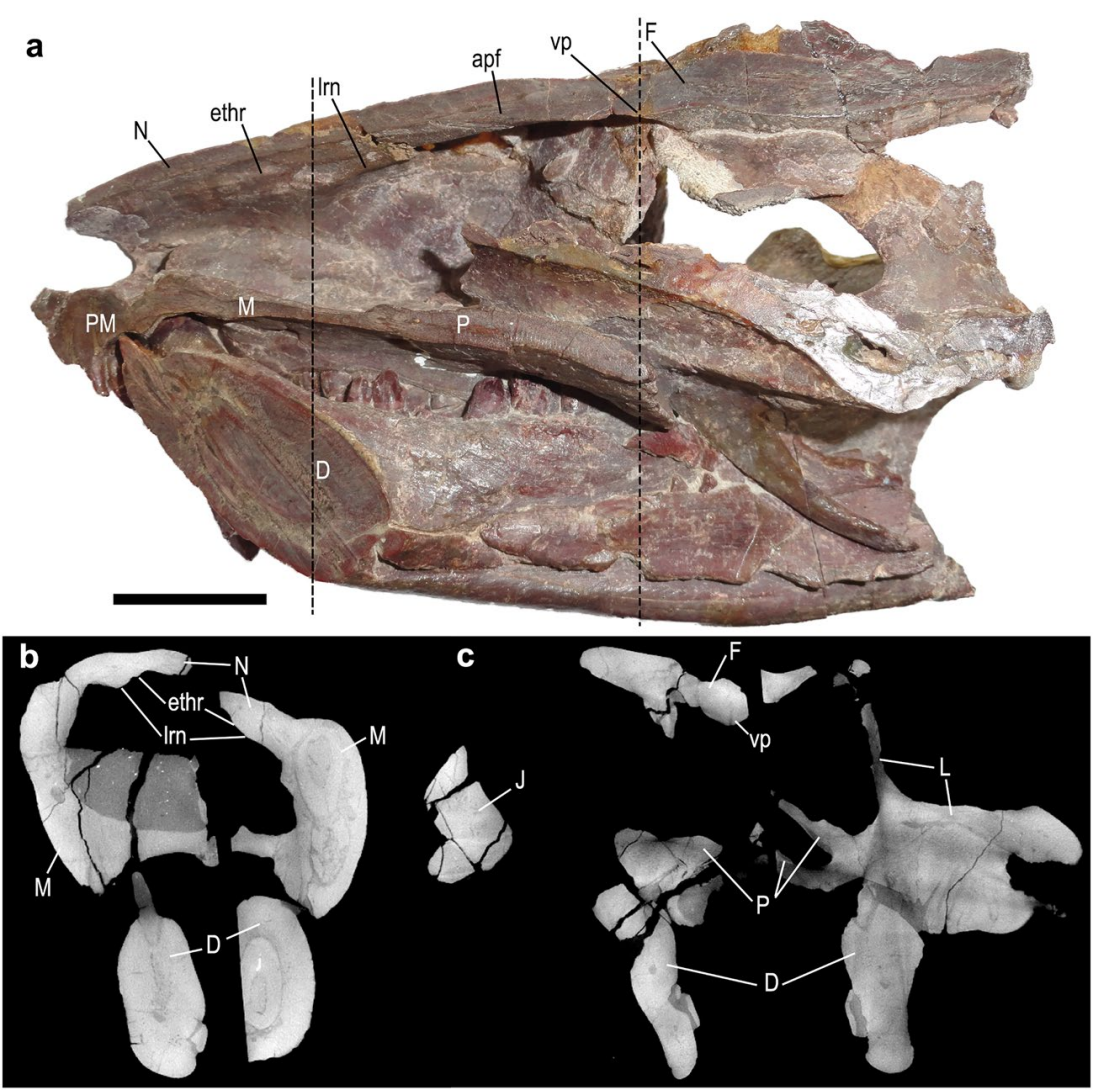
Skull of *Chiniquodon theotonicus* (NHMUK PV R8429). (**a**) Skull section in sagittal line exhibiting the right half. (**b**) Transverse section slice of the anterior portion of the nasal region. (**c**) Transverse section slice of the posterior portion of the nasal region. The dotted line corresponds to the point of the transverse section slice. apf, anterior projection of the frontal; D, dentary; F, frontal; ethr, ethmoid ridge of nasal; J, jugal; L, lacrimal; lrn, lateral ridge of nasal; M, maxilla; N, nasal; P, palatine; PM, premaxilla; vp, ventral projection. Scale bar 1 cm.

**Figure 4:**
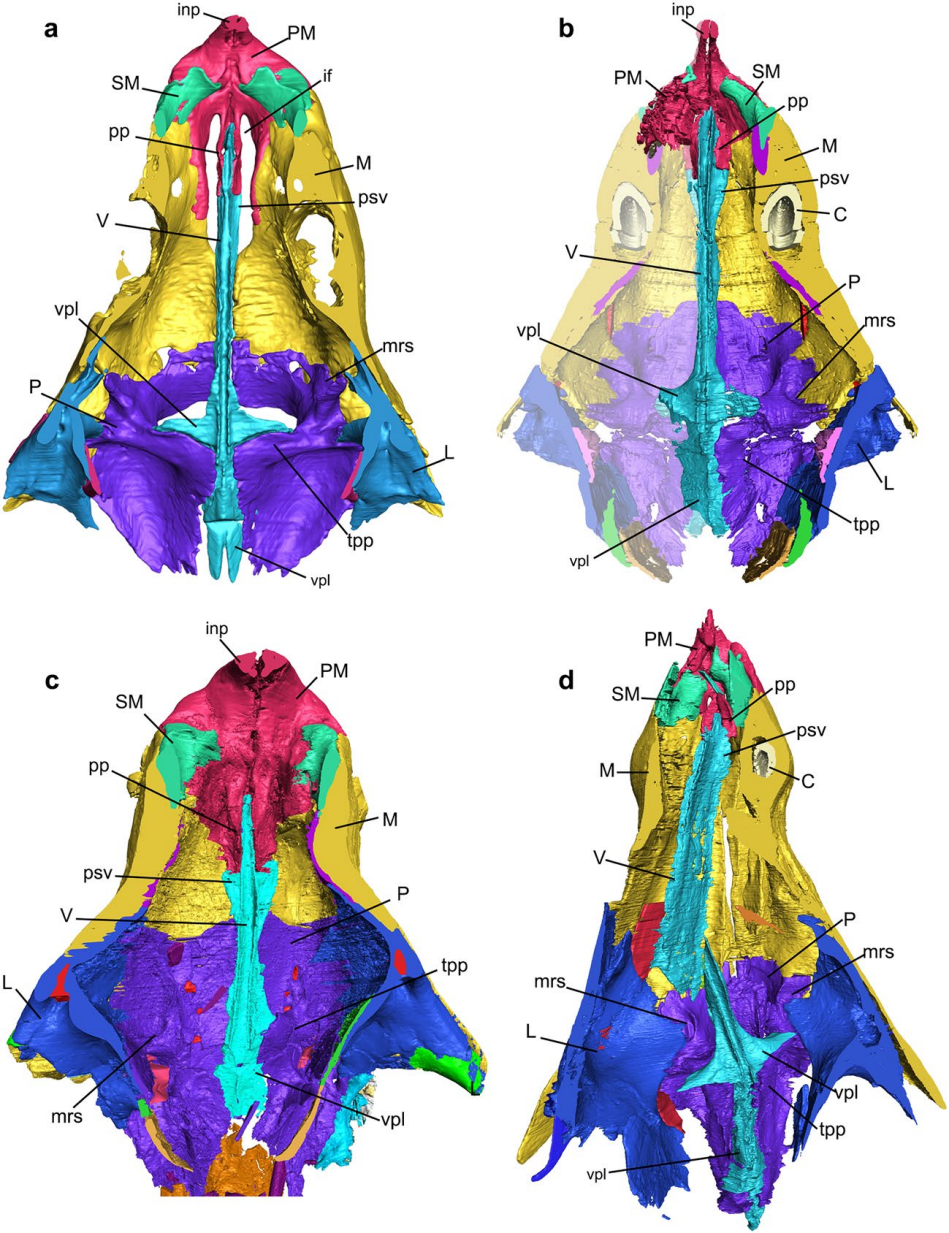
Nasal cavities of the studied cynodonts. (**a–d**) Skull section in dorsal view, exhibiting the dorsal region of the floor of the nasal cavity in *Thrinaxodon liorhinus* (NHMUK PV R511) (**a**), *Prozostrodon brasiliensis* (UFRGS-PV-248-T, left half of the skull is mirrored) (**b**), *Riograndia guaibensis* (UFRGS-PV-596-T) (**c**), and *Brasilodon quadrangularis* (UFRGS-PV-1043-T) (**d**). Lighter region in (**b**) indicates mirroring. C, canine; if, incisive foramen; inp, internarial process of premaxilla; L, lacrimal; M, maxilla; mrs, maxillary recess; P, palatine; PM, premaxilla; pp, palatine process of the premaxilla; psv, paraseptal shelf of the vomer; SM, septomaxilla; tpp, transverse process of the palatine; V, vomer; vpl, vomerine plate.

**Figure 5:**
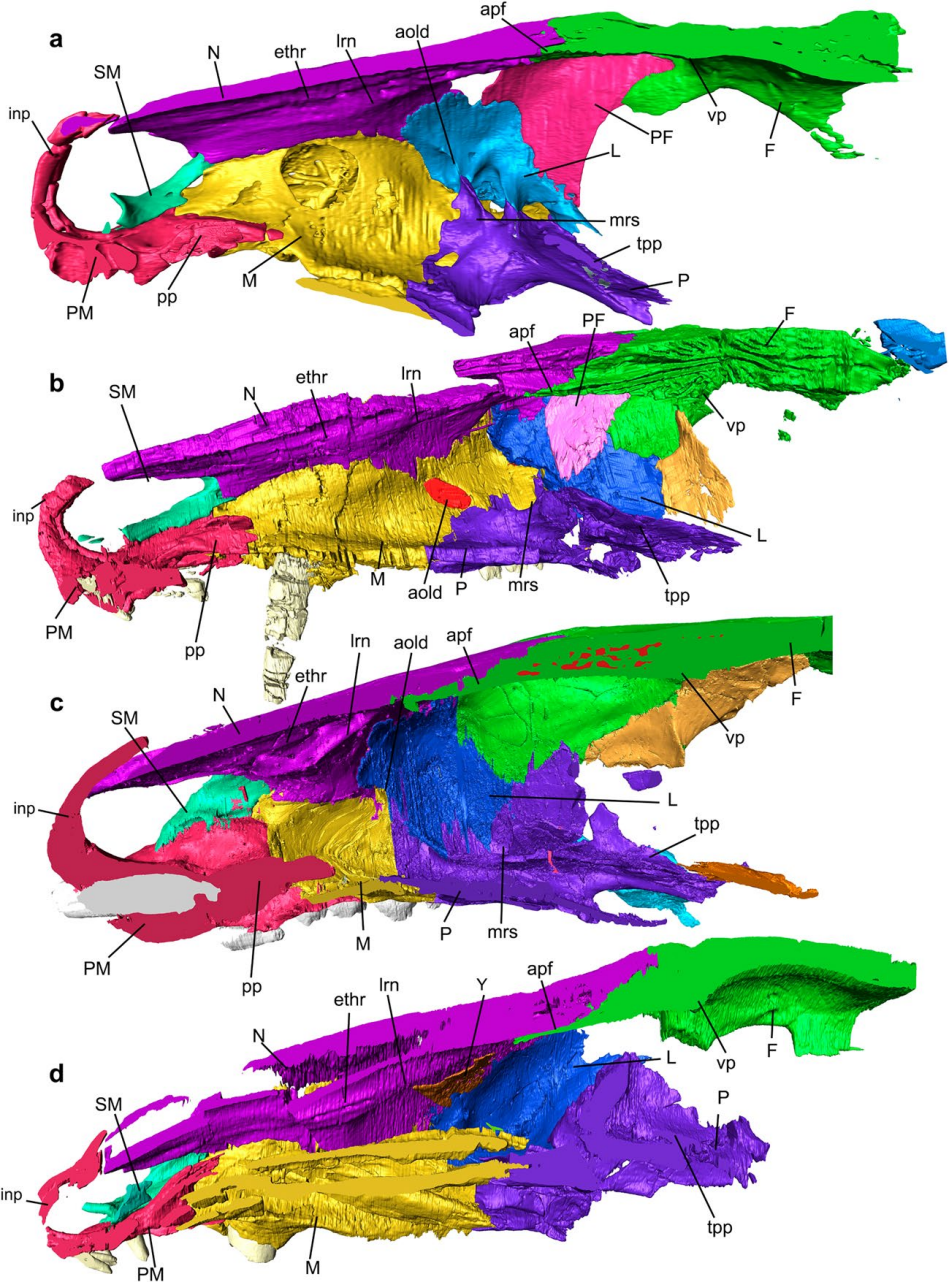
Nasal cavities of the studied cynodonts. (**a–d**) Right half of the skull in lateral view, exhibiting the lateral wall of the nasal cavity, in *Thrinaxodon liorhinus* (NHMUK PV R511) (**a**), *Prozostrodon brasiliensis* (UFRGS-PV-248-T, left half of the skull is mirrored) (**b**), *Riograndia guaibensis* (UFRGS-PV-596-T) (**c**), and *Brasilodon quadrangularis* (UFRGS-PV-1043-T) (**d**). aold, anterior opening of lacrimal duct; apf, anterior projection of the frontal; ethr, ethmoid ridge of nasal; F, frontal; inp, internarial process of premaxilla; L, lacrimal; lrn, lateral ridge of nasal; M, maxilla; mrs, maxillar recess; N, nasal; P, palatine; PF, prefrontal; PM, premaxilla; pp, palatine process of the premaxilla; SM, septomaxilla; tpp, transverse process of the palatine; vp, ventral projection; Y, structure identified as a turbinal by Ruf et al.^29^.

**Figure 6:**
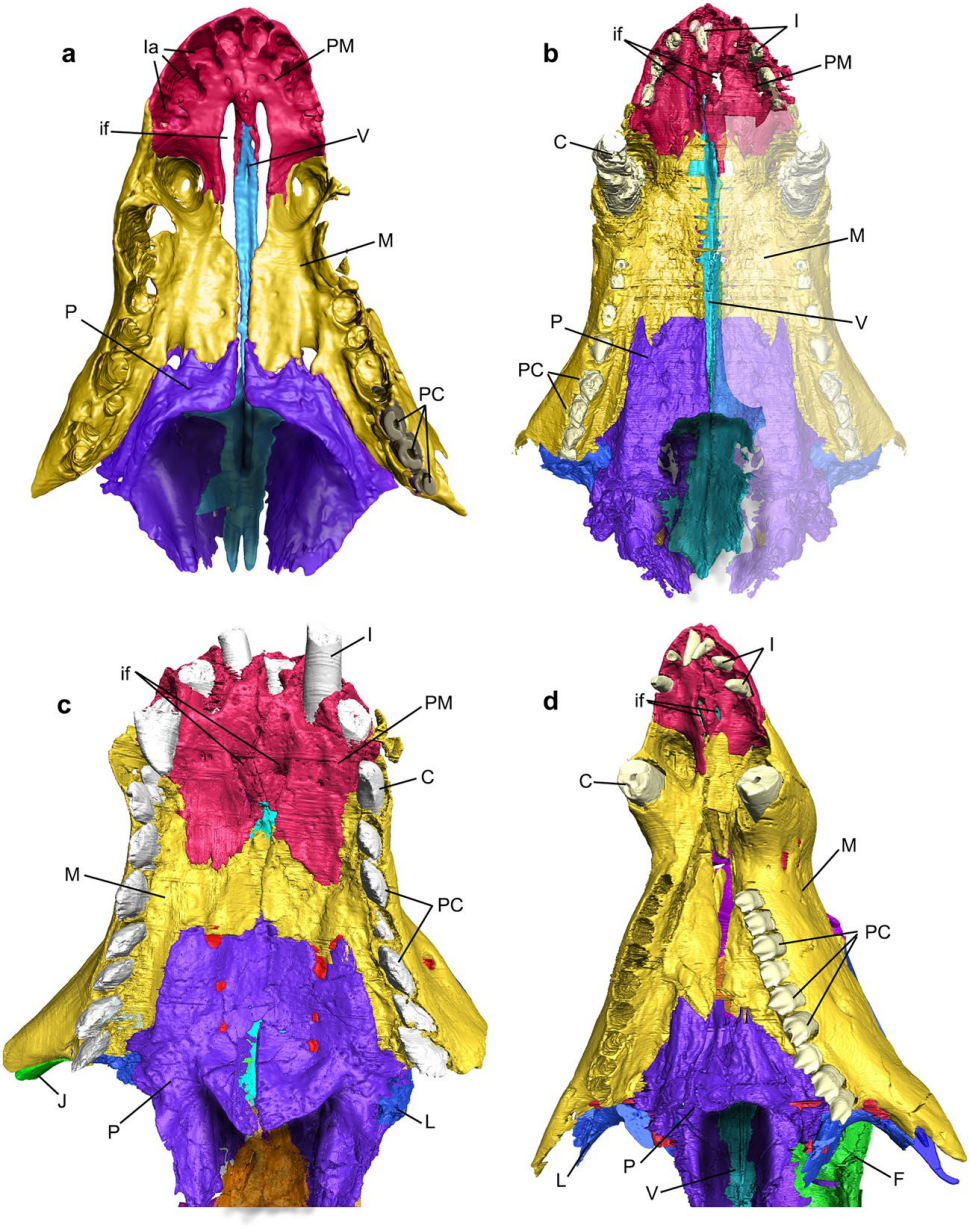
Nasal cavity in the studied cynodonts. (**a–d**) Ventral view of the palatal region, exhibiting the secondary palate and the border of the secondary choana, in *Thrinaxodon liorhinus* (NHMUK PV R511) (**a**), *Prozostrodon brasiliensis* (UFRGS-PV-248-T, left half of the skull is mirrored) (**b**), *Riograndia guaibensis* (UFRGS-PV-596-T) (**c**), and *Brasilodon quadrangularis* (UFRGS-PV-1043-T) (**d**). Lighter region in (b) indicates mirroring. C, canine; F, frontal; I, incisive; if, incisive foramen; J, jugal; L, lacrimal; la, upper incisor alveolus; M, maxilla; P, palatine; PC, upper postcanine; PM, premaxilla; V, vomer.

**Figure 7:**
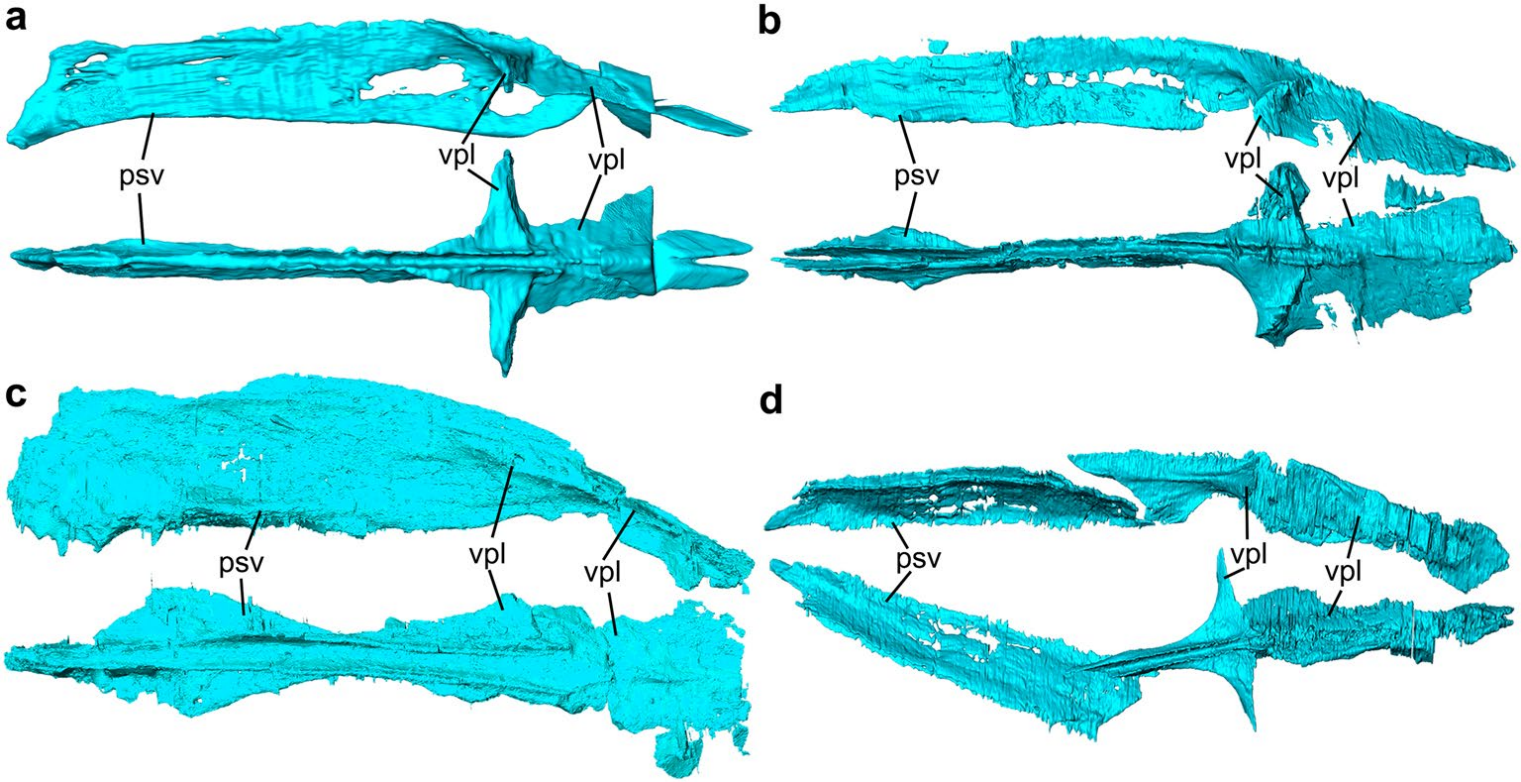
Three-dimensional model of the isolated vomer in the studied cynodonts. (**a–d**) Vomer of *Thrinaxodon liorhinus* (NHMUK PV R511) in left lateral (above) and dorsal (below) views (**a**), *Prozostrodon brasiliensis* (UFRGS-PV-248-T) in lateral (above) and dorsal (below) views (**b**), *Riograndia guaibensis* (UFRGS-PV-596-T) in lateral (above) and dorsal (below) views (**c**), and *Brasilodon quadrangularis* (UFRGS-PV-1043-T) in lateral (above) and dorsal (below) views (**d**). psv, paraseptal shelf of the vomer; vpl, vomerine plate.

The rostrum includes the nasal cavity that is enclosed by the premaxillae, septomaxillae, maxillae, nasals, part of the frontals, lacrimals, vomer, and palatines in *Riograndia* and *Brasilodon*, whereas in *Thrinaxodon*, *Chiniquodon*, and *Prozostrodon* the prefrontals also limit this cavity anteroposteriorly (Figs. 2, 3, 4, 5, 6, 7). The anterior portion of the nasal cavity includes the external nares that are divided by the internarial bar in the studied species. The internarial bar is formed entirely by the premaxillae and fits dorsally into a medial notch of the nasals (Figs. 4, 5). In *Riograndia* (Figs. 4c, 5c), the base of the internarial bar is transversely wider and longer than in *Thrinaxodon*, *Chiniquodon*, *Prozostrodon*, and *Brasilodon*. Posteriorly, the nasal cavity is divided into two areas: the olfactory region (dorsal) and the nasopharyngeal duct (ventral), which are divided by the vomer and palatine. In the dorsal portion, the posterior border remains opened, lacking an ossified posterior wall that appears in mammals (i.e., the cribriform plate), and the limits between the nasal cavity and the mesocranium (that is a portion of the olfactory bulbs and the unossified orbital vacuity) are marked dorsally by the transversal crest of the frontal and ventrally by the anteroventral border of the orbital vacuity, which corresponds to the area above the transverse lamina.

The floor of the nasal cavity is formed by the premaxillae, maxillae, palatines, and vomer (Figs. 4, S2). In the anteriormost portion, the premaxilla forms the ventral surface and the border of the external nares, contacting laterally and posteriorly the maxilla. In *Chiniquodon*, *Prozostrodon*, *Riograndia*, and *Brasilodon*, the premaxilla forms the anterior portion of the secondary osseous palate through the medial contact of the palatal process. In *Thrinaxodon* the secondary bony palate is incomplete since the palatal processes (Fig. 6a) of the maxilla do not contact in the medial line (see the discussion of Jasinoski et al.^83^, erroneously cited by Pusch et al.^80^ as complete). Due to the reduced length of the snout of *Riograndia*, the floor of the nasal cavity in this taxon presents a higher participation of the premaxilla than the maxilla (Figs. 4c, S2c), when compared with *Thrinaxodon*, *Chiniquodon*, *Prozostrodon,* and *Brasilodon*.

The nasal cavity is divided into respiratory (anteriorly) and olfactory (posteriorly) regions, although a clear limit between both regions cannot be clearly recognized. The respiratory region is smaller than the olfactory region. In the ventral portion of the nasal cavity runs the nasopharyngeal duct (or nasopharyngeal passage) which is divided into two portions (Figs. 3, 4, 5), and delimited from the remainder of the nasal cavity by the vomer. The nasopharyngeal duct connects the respiratory region to the posterior region of the mouth, where the secondary choana is located. In *Thrinaxodon*, the floor of the nasopharyngeal duct opens once the palatal processes of the maxilla does not contact the medial line (Fig. 6). Probably, the floor of the nasopharyngeal duct in *Thrinaxodon* is formed by unossified cartilage^66^. The regions that comprise the nasal cavity of the five analysed taxa will be described in the following sections.

### The roof of the nasal cavity

#### Nasal

The general condition present in non-mammaliaform cynodonts is that the roof of the nasal cavity is formed by the nasal, anteriorly, and by the anterior portion of the frontal, posteriorly (Figs. 2, S1). The nasal forms the dorsal and lateral portions of the lateral wall of the cavity (Figs. 2, 3, 4, 5). Also, the anterior border of the nasal forms the contour of the external nares (Figs. 4, S2). Posteriorly, the nasal overlaps the frontal.

In the medial contact of both nasals, there is a median crest (mc) (sensu Kermack et al.^81^ and Crompton et al.^31^) that runs posteriorly and continues on the ventral surface of the frontal (see below). In all studied specimens the median crest progressively increases in height posteriorly (Figs. 2, S1). In *Brasilodon* this crest is more developed than in *Thrinaxodon*, *Chiniquodon*, *Prozostrodon*, and *Riograndia* (Fig. 2). In *Thrinaxodon* and *Chiniquodon* the nasal portion of the median crest is weakly marked in comparison to other specimens. In *Riograndia* (Figs. 2c, S1c) the length of the median crest is reduced in comparison to *Brasilodon*, *Chiniquodon*, and *Thrinaxodon*. The holotype of *Prozostrodon* presents the nasal broken but the median crest is evident along the fragments (Figs. 2b, S1b).

Parallel to the medial crest (mc), two ridges delimit a groove that runs along the entire length of the nasals and reaches the ventral surface of the frontal in the five taxa (Figs. 2, S1). These ridges are similar to those described in the same position in the roof of the nasal cavity of *Morganucodon* (figs. 19, 20, 21, 22, 25, 27, 28, 30, and 100 in Kermack et al.^75^). Kermack et al.^81^ named the most lateral ridge as the lateral ridge of the nasal (lrn), and the most medial ridge as the ethmoid ridge (ethr). Although they have some morphological differences, probably these ridges are homologous to those present in *Thrinaxodon*, *Chiniquodon*, *Prozostrodon*, *Riograndia*, and *Brasilodon*; therefore, we use the same nomenclature (see Fig. 2). In *Thrinaxodon* (NHMUK PV R511) and *Chiniquodon* (NHMUK PV R842), the ethmoid and lateral ridges are parallel and rectilinear, and run along the entire length of the roof of the nasal cavity until close to the transverse crests (tc; Figs. 2a, 3a), which are two smaller crests, transversely positioned on each side of the ventral projection (vp) of the medial crest, on the ventral surface of the frontal. In both specimens, the lateral and ethmoidal ridges are well marked for their entire length, being formed anteriorly by the nasal and posteriorly by the frontal. In *Prozostrodon* (UFRGS-PV-248-T), *Riograndia* (UFRGS-PV-596-T, MCN-PV 2264), and *Brasilodon* (UFRGS-PV-929-T) (Fig. 2b–d), the ethmoid and lateral ridges follow the contour of the nasal cavity, diverging from the medial region at the level of expansion of the nasal cavity, reaching the frontal. Furthermore, in *Riograndia* and *Brasilodon* both ridges are less marked in the posterior portion of the nasal, close to the contact with the frontal, differently from *Prozostrodon* in which the ridges are continuous until near the transverse crests, in the ventral surface of the frontal (Figs. 2b,c, S1b,c). In *Prozostrodon*, *Riograndia*, and *Brasilodon*, the lateral ridges of the nasal are well developed and project medially in comparison to the ethmoid ridge (rthr; Fig. 2b,c).

These crests have been recorded in other non-mammaliaform cynodonts such as *Procynosuchus*, *Galesaurus*, *Nythosaurus*, *Diademodon*, *Exaeretodon*, *Luangwa*, *Oligokyphus*, and other specimens of *Thrinaxodon*^19,36,78^. Most of these authors identified the ridges parallel to the medial ridge as ridges for cartilaginous nasal turbinals^19,36,78^, which are likely homologous to the lateral and ethmoid ridges here identified. However, the homologies for these structures among cynodonts and other therapsids have to be explored deeper, and is beyond the scope of this contribution.

The grooves formed by the ethmoidal and lateral ridges are divided into two branches anteriorly, that end in the posterior border of the external nares, close to contact with the septomaxilla (Figs. 2, S1). In *Thrinaxodon*, *Chiniquodon*, and *Brasilodon* the grooves are weakly marked in comparison to *Prozostrodon* and *Riograndia*. Furthermore, the posterior region of the grooves gradually reduces in depth in *Prozostrodon*, *Riograndia*, and *Brasilodon,* disappearing before the contact with the frontal (Fig. 2c,d). In *Chiniquodon* the grooves are poorly marked all along their length, although more marked in the posterior portion. In *Prozostrodon*, *Riograndia*, and *Brasilodon*, the grooves are weakly marked at the level of the nasal expansion, whereas in *Thrinaxodon* the ridges are marked until the level of the transverse crests (tc; Figs. 2, S1). Similar grooves are described for *Morganucodon* by Kermack et al.^81^ named as ethmoidal vessels trough. In the reconstruction made by Kermack et al.^81^ (fig. 100) those grooves are well marked in *Morganucodon* and extended over the frontal bone; however, the illustrations of the nasal bones show that the grooves (fig. 19 in reference^81^) are less marked and seem to be more similar to the condition found in non-mammaliaform cynodonts.

The grooves in the ventral surface of the nasals of *Thrinaxodon*, *Chiniquodon*, *Prozostrodon*, *Riograndia*, and *Brasilodon* are associated with from two to five foramina, which open onto the dorsal surface of the nasal (black arrows in Figs. 2, S1). *Thrinaxodon* NHMUK PV R511 (Figs. 2a, S1a) preserves five foramina; the anterior one is at the level of the posterior border of the canine root, the second and third at the level of the anterior border of the expansion of the nasal, and the fourth most posteriorly. In *Chiniquodon* NHMUK PV R842 at least five foramina were identified; the anterior one placed anterior to the nasal constriction and the others posterior to it. In *Prozostrodon* UFRGS-PV-248-T (Figs. 2b, S1b), three foramina are identified, with the most anterior placed at the nasal constriction and the two others next to the contact of the nasal with the frontal. *Riograndia* UFRGS-PV-596-T has five openings preserved foramina (Fig. 2c). The anteriormost foramen is the largest, located near the edge of the external nares, the second is located at the level of the posterior border of the septomaxilla, and the third, fourth, and fifth foramina are located near to the suture between the nasal and maxilla. The fifth foramen is the smallest and lies slightly ventral to the fourth one. In *Brasilodon* UFRGS-PV-1043-T and UFRGS-PV-929-T (Figs. 2d, S1d), there are three foramina in each groove; the anterior one is anterior to the nasal constriction and exits dorsally, whereas the posteriormost is at the end of the groove and branches into two exits dorsally. Kermack et al.^81^ described three foramina for *Morganucodon* in the lateral groove of the ventral surface of the nasal. It is interesting to note that in *Riograndia* UFRGS-PV-596-T and MCN-PV 2264, the three posteriormost foramina open dorsally inside the contact between the nasal and maxilla. In the other non-mammaliaform cynodonts the foramina open in the dorsal surface of the nasal.

#### Frontal

In general, the anteriormost region of the frontal is formed by the anterior projection of the frontal (apf) (sensu Ruf et al.^29^) that extends below the nasal and forms a platform that supports this bone. The anterior projection of the frontals has a uniform and convex anterior edge in *Thrinaxodon*, *Prozostrodon*, and *Riograndia*. In *Prozostrodon*, *Riograndia*, and *Brasilodon* the anterior projection of the frontal is more projected than in *Thrinaxodon* (Figs. 2, S1). In the latter the anterior border has a medial rectangular projection and is anteroposteriorly shorter than in the aforementioned genera. In the medial line of the anterior portion of the frontal is the continuation of the median crest (sensu Kermack et al.^81^ and Crompton et al.^31^) that started in the nasal (Fig. 2). This crest grows in size posteriorly, with its deepest part at the level of the end of the nasal cavity (Figs. 2, S1).

In *Thrinaxodon* and *Chiniquodon* the ventral projection (vp) is weakly marked compared to *Prozostrodon*, *Riograndia*, and *Brasilodon* (Figs. 2, 3). In *Prozostrodon* the median crest is relatively developed but less than in *Riograndia* and *Brasilodon*. *Brasilodon* has the most developed medial crest (mc) compared to other taxa. In *Prozostrodon* (UFRGS-PV-248-T) the median crest and ventral projections appear to be formed by the thickening of the dorsal portion of the frontal, and this condition causes the roof of the nasal cavity to have a triangular shape in cross section. Therefore, *Prozostrodon* presents a singular condition in comparison to *Thrinaxodon*, *Chiniquodon*, *Riograndia*, and *Brasilodon*, with the crest being restricted to the medial portion of the ventral surface. In *Riograndia* the posterior portion of the median crest that corresponds to the ventral projections is developed and presents a conical shape, similar to a callus. This is due to the median crest increasing in size abruptly, differing from the condition seen in other taxa, where the crest increases progressively culminating in the posterior portion of the frontal bone (Fig. 2).

Lateral to the ventral medial projection, all specimens present two short transverse crests (tc) that smooth laterally (Fig. 2), not reaching the descending process of the frontal, named transverse crests (sensu Kermack et al.^81^ and Crompton et al.^31^). The transverse crests form part of the posterodorsal boundary between the nasal cavity and the olfactory bulbs. Hence, the posterior surface of the transverse crests is more concave than the anterior portion, due to the development of the olfactory bulbs. The transverse crests are transversely oriented in *Riograndia* (Fig. 2c), but obliquely placed (anteromedial to posterolaterally) in *Thrinaxodon*, *Prozostrodon*, and *Brasilodon* (Fig. 2a,b,d). The transverse crests are notably more marked in *Brasilodon* than in *Thrinaxodon*, *Chiniquodon*, *Prozostrodon*, and *Riograndia*. In *Thrinaxodon* and *Prozostrodon* they are less distinct in comparison to *Riograndia* and *Chiniquodon* due to the presence of a depression anterior to the transverse crest in the last two taxa. Furthermore, in *Brasilodon* a tiny crest is recognized in the ventral surface of the transverse crests in the specimen UFRGS-PV-929-T and weakly marked in the specimen UFRGS-PV-1043-T (Fig. 2d). The anterior portion of this tiny crest starts medially at the ventral surface of the transverse crests and ends at the posteriormost edge (Figs. 2d, S1d).

In all specimens the posterior edge of the ventral projections and transverse crests (tc) form the anterodorsal limits of the brain cavity, more specifically the anterodorsal edge of the olfactory bulb (Fig. 2). In *Thrinaxodon*, *Prozostrodon*, *Riograndia*, and *Brasilodon* the medial crest (mc) presents a tiny continuation in this region that abruptly reduces its size and disappears (Fig. 2). In *Chiniquodon* this structure extends a little further posteriorly than the other taxa (Fig. 3). This continuation of the medial crest marks the division of the two bulbs. *Galesaurus*^36^ and *Probainognathus*^31^ have two low ridges in the same region.

In *Thrinaxodon*, *Prozostrodon*, and *Riograndia*, crests parallel to the medial crest are observed on the ventral surface of the frontal, anterior to the transverse crests. In *Thrinaxodon* they are the two parallel ridges that continue the extension of the ethmoidal ridge and the lateral ridge of the nasal (Fig. 2a). In *Prozostrodon* only one ridge is observed and, despite not being directly connected to the nasal ridge, the frontal ridge is aligned with the nasal ridge (Fig. 2b). In *Riograndia* two parallel ridges are faintly marked at this position (Fig. 2c). These ridges delimit the medial border of the expansion of the olfactory region of the nasal cavity.

### Floor of the nasal cavity

#### Premaxilla

The premaxilla forms the anterior and ventral region of the nasal cavity. The anteriormost portion of the premaxilla forms the border of the external nares and the internarial bar (Figs. 4, 5). The premaxilla contacts the septomaxilla anterolaterally (Figs. 4, S2). In the contact with the maxilla, the premaxilla forms a small part of the lateral and ventral portion of the anterior region of the nasal cavity, and the anterior and lateral portion of the incisive foramen in *Chiniquodon*, *Prozostrodon*, *Riograndia*, and *Brasilodon*. Otherwise, in *Thrinaxodon* the premaxilla forms the anterior border of the incomplete secondary bony palate (Figs. 4a, S2a).

In the inner nasal cavity, in the medial line, the premaxilla contacts the anterior portion of the vomer through the palatine process in all studied species (Fig. 4). In *Thrinaxodon*, *Chiniquodon*, *Prozostrodon*, and *Brasilodon*, the dorsal region of the palatine process of the premaxilla (pp) is formed by a thin lamina that contacts the lateral wall of the anterior portion of the vomer (Figs. 4a,b,d, S2). In *Riograndia* the dorsal portion of the palatine process has a flat surface that is at the same level as the dorsal region of the vomer (Fig. 4c). The ventral portion of the palatine process presents a lateral flat expansion that forms a shelf that continues to the paraseptal shelf of the vomer (psv). This surface covers the incisive foramen passage in dorsal view, in *Chiniquodon*, *Prozostrodon*, *Riograndia*, and *Brasilodon*. This surface does not prevent the opening of the incisive foramen, but limits it dorsally, so that the passage of the incisive foramen is restricted to a lateral opening, between the lateral branch of the premaxilla and the palatine process and the posterior region. In *Thrinaxodon* the ventral projection of the palatine process is reduced and does not cover the opening present in the secondary palate, which remains incomplete in this taxon (see discussion in reference^83^).

In general, the incisive foramen is formed by the premaxilla and maxilla and is located posteriorly to the upper incisor teeth, at the medial line. The anterior border is formed by the premaxilla, the posterior border by the maxilla, and medially the palatine process of the premaxilla articulates with the vomer to enclose the incisive foramen dorsally (pp—Figs. 4, S2). In *Thrinaxodon* the vomer is widely exposed ventrally through the incisive foramen, once the secondary plate remains open, whereas in *Chiniquodon*, *Prozostrodon*, *Riograndia*, and *Brasilodon* a small part of the anterior portion of the vomer, that articulates with the palatine process of the premaxilla, appears through the posterior region of the incisive foramen (Figs. 4, 6). In *Thrinaxodon* the palatine process of the premaxilla is narrow and does not cover the foramen dorsally, in ventral view. In *Brasilodon*, *Chiniquodon*, and *Prozostrodon*, the lateral expansion in the posterior portion of the palatine process of the premaxilla dorsally covers the posteriormost portion of the incisive foramen, whereas in *Riograndia* the lateral expansion of the palatine process completely covers the incisive foramen dorsally.

#### Septomaxilla

In general, the septomaxilla forms the ventral and posterior border of the external nares (Figs. 5, S3). In *Prozostrodon* and *Riograndia* the septomaxilla participates in the posterodorsal portion of the border of the external nares. Whereas in *Prozostrodon* the participation in the dorsal border of the external nares is reduced, in *Riograndia* the septomaxilla shows an anterior projection in the dorsal border. In the ventral portion the septomaxilla contacts the dorsal surface of the premaxilla in *Thrinaxodon*, *Riograndia*, and *Brasilodon*. In *Riograndia* (Fig. 5c) this contact is wider in the dorsal view. In *Prozostrodon* the ventral region is thin and the anterior portion of the septomaxilla is projected anterodorsally and does not contact the premaxilla (Figs. 5b, S3b). In *Thrinaxodon* the ventral portion of the septomaxilla is wider and contacts the dorsal surface of the premaxilla, similar to *Riograndia*, but in the anterior portion of the ventral region, the septomaxilla presents an anterodorsal projection (Figs. 5a, S3a). The projections seen in *Prozostrodon* and *Thrinaxodon* are different. Whereas in *Prozostrodon* it only rises in relation to the premaxilla, in *Thrinaxodon* the projection forms a concavity facing anteriorly, in the shape of a “C”. In *Chiniquodon*, *Prozostrodon*, *Riograndia*, and *Brasilodon*, the posteriormost portion of the septomaxilla contacts the maxilla laterally, and is overlapped by the descendent process of the nasal medially. This condition excludes the septomaxilla from the lateral wall of the nasal cavity in these taxa. In *Thrinaxodon* the septomaxilla only contacts the anterior border of the maxilla.

#### Maxilla

The maxilla forms the ventral and lateral walls of the nasal cavity (Figs. 4, 5). This bone has three main components: the facial process, the alveolar space for the canine and postcanine teeth and the palatal projection. Anteriorly it contacts the premaxilla and septomaxilla and dorsally the nasal, posteriorly the facial process contacts the lacrimal and the palatal projection reaches the palatine. In *Chiniquodon*, *Prozostrodon*, *Riograndia*, and *Brasilodon* both maxillae contact each other medially, forming part of the secondary palate, and the vomer dorsally to the medial line (Figs. 4, S2). In *Thrinaxodon* the maxillae do not contact in the medial line and do not contact the vomer (Fig. 6a), due to the incomplete secondary palate (see Jasinoski et al.^83^). In all analysed taxa, the palatal region of the maxilla is flat and contacts the palatine posteriorly. In *Riograndia* (UFRGS-PV-596-T) the participation of the maxilla in the floor of the nasal cavity is reduced in comparison to the other taxa (Figs. 4c, 6c), probably due to the reduction of the snout.

In all taxa, the anterior portion of the palatal region of the maxilla continues the depression formed by the opening of the incisive foramen of the premaxilla, becoming deeper in its medial region than in the lateral portion. In the lateral region of the palatal process, the maxilla continues the posterior portion of the premaxilla which forms a shelf that reduces its size posteriorly until it disappears in the lateral wall of the nasal cavity. In *Thrinaxodon* and *Chiniquodon* the shelf is smaller than in *Prozostrodon*, *Riograndia*, and *Brasilodon*, being more developed in *Riograndia* and *Brasilodon*.

In the lateral wall, the descending process of the nasal overlaps the most dorsal region of the facial process of the maxilla in *Thrinaxodon*, *Chiniquodon*, *Prozostrodon*, *Riograndia*, and *Brasilodon* (Figs. 5, S3). The exposed part of the maxilla on the lateral wall has a triangular shape in *Thrinaxodon*, *Prozostrodon*, and *Brasilodon*, with its anterior region small that expands dorsally posteriorly. In *Riograndia* (UFRGS-PV-596-T) the exposed part of the maxilla is reduced in comparison to the other taxa, probably due to the snout shortening. In *Chiniquodon* it was not possible to identify the limits of the nasal and maxilla in the lateral wall of the nasal cavity due to the tomography resolution (Fig. 3). In *Thrinaxodon* NHMUK PV R511 (Fig. 5a) the lateral wall presents three internal openings probably resulting from tooth resorption processes. In both sides of the skull, the first opening is positioned at the level of the paracanine fossa, the second is the largest and is positioned in the root of the canine, and the third one is positioned at the level of the second postcanine. The openings do not have regular shapes and differ between the two sides of the skull.

The anterior opening of the lacrimal duct is positioned in the posterior portion of the lateral wall of the nasal cavity, in contact between the lacrimal and the facial process of the maxilla. In *Riograndia* and *Brasilodon* the anterior opening of the lacrimal duct is formed by the maxilla laterally and by the lacrimal medially. Meanwhile, in *Thrinaxodon* the anterior opening of the lacrimal duct is completely formed by the lacrimal, and in *Prozostrodon* the anterior opening is completely formed by maxilla, both differing from the condition of *Riograndia* and *Brasilodon*. Furthermore, the anterior opening of the lacrimal duct is slightly slit-shaped and is facing anteroventrally in *Thrinaxodon* and *Riograndia*. In *Thrinaxodon* and *Riograndia*, the anterior opening of the lacrimal duct is more dorsal on the lateral wall, compared to *Prozostrodon* and *Brasilodon*.

In *Thrinaxodon* and *Riograndia* the anterior opening of the lacrimal duct does not present a nasolacrimal groove (Fig. 5a,c). In *Prozostrodon* and *Brasilodon* (Fig. 5b,d) the anterior opening of the lacrimal duct is rounded and presents a nasolacrimal groove positioned anteriorly. In *Prozostrodon* the nasolacrimal groove is shorter but deeper than in *Brasilodon*. In *Brasilodon* (UFRGS-PV-1043-T) this groove is dorsoventrally wider and its borders are much less marked and it gets shallower until it disappears anteriorly. This is in contrast to *Morganucodon* where the groove is narrower and longer (see in Kermack et al.^81^). In *Brasilodon*, the anterior opening of the lacrimal duct and the nasolacrimal groove faces anteriorly, whereas in *Prozostrodon* it faces anterodorsally. Furthermore, the maxillary canal (sensu^13,14^) is visible dorsally through the nasolacrimal groove in *Brasilodon* and *Prozostrodon*, indicating that they are not completely separated by ossification.

In *Thrinaxodon*, the posterodorsal edge of the anterior opening of lacrimal duct has a crest that is confluent with the crest that delimits ventrally the maxillary recess (Figs. 5a, S3a). In *Riograndia*, the dorsal edge of the anterior opening of lacrimal duct is confluent with the projection of the lateral wall, similar to a shelf, which delimits the dorsal region of the ventral region of the nasal cavity (Figs. 5c, S3c). This projection is preserved only in the right side and does not resemble a ridge but rather a thickening of the wall. Finally, *Prozostrodon* and *Brasilodon* do not present crests in the region anterior to the anterior opening of the lacrimal duct.

It is important to mention that, when removing the nasal, we observed a ridge on the ventral limit of the contact surface on the descending process of the nasal with the maxilla in *Brasilodon*. Furthermore, both *Brasilodon* (UFRGS-PV-1043-T) and *Riograndia* (UFRGS-PV-596-T) present the ventral edge of the descending process of the nasal slightly folded medially, which gives the impression of forming a crest. However, in other specimens (e.g., UFRGS-PV-833-T, UFRGS-PV-929-T, UFRGS-PV-1030-T) we observed that the descending process of the nasal is continuous with the lateral wall of the nasal cavity, which leads us to believe that this fold is an effect of the taphonomic processes.

#### Palatine

In all species the palatine forms the posterior portion of the floor of the nasal cavity, which comprises the posterior border of the secondary osseous palate through contact between the two palatal branches of the palatine (Figs. 4, S2). The palatine also participates in the formation of the nasopharyngeal duct through the contact between the transverse process of the palatine (tpp) with the vomerine plate (vpl) of the vomer, and the formation of the ventral part of the lateral wall of the nasal cavity.

In the medial line of the posterior portion of the secondary osseous palate, the contact of the two palatal branches of the palatine forms a ridge that contacts the vomer ventrally in *Prozostrodon*, *Riograndia*, and *Brasilodon* (Figs. 4, S2). This ridge gives continuity to the crests present in the contact of the palatal branches of maxillae, which has its greatest size on the palatine. In *Thrinaxodon*, there is no contact between the palatines in the sagittal line, where the secondary osseous palate remains incomplete^83^.

The palatine forms the ventral, lateral and part of the dorsal area of the nasopharyngeal duct (Figs. 4, 5, S2, S3). Hillenius^19^ and Crompton et al.^31^ define the dorsal region of the nasopharyngeal duct as a transverse lamina that is formed by the transverse process of the palatine (or palatine flanges sensu Crompton et al.^31^) and the vomerine plate of the vomer (vpl). The transverse process of the palatine (tpp) together with the vomerine plate of the vomer, form the transverse lamina (or primary plate), that forms the roof of the nasopharyngeal duct in all cynodonts^31,65^ (see also Vomer section here). In non-mammaliaform cynodonts the transverse process of the palatine is flattened and overlaps the vomerine plate (Figs. 4, S2). In *Thrinaxodon*, *Prozostrodon*, and *Brasilodon*, the anteromedial border of the transverse process presents a notch that articulates with the anteromedial border of the vomerine plate, leaving the vomer exposed in dorsal view, in the transverse lamina. In *Brasilodon* (UFRGS-PV-929-T, UFRGS-PV-1030-T, and UFRGS-PV-1043-T; see also see Fig. S7), the anterior border of the transverse lamina bends dorsally, forming a ridge. Both the transverse process of the palatine and the anterior edge of the vomerine plate participate in this ridge. The posterolateral portion of the transverse process of the palatine presents a depression in *Thrinaxodon* and *Prozostrodon*, being more strongly marked in the first taxon. In *Riograndia* and *Brasilodon*, the posterior portion of the transverse lamina is inclined laterally, but does not show the depression seen in *Thrinaxodon* and *Prozostrodon* (Fig. 4).

In the lateral wall of the nasal cavity, in *Thrinaxodon*, *Chiniquodon*, *Prozostrodon*, *Riograndia*, and *Brasilodon*, the palatine presents an anterodorsally facing concavity. In all these taxa, this structure is located in the lateralmost region of the transverse process of the palatine, next to the lateral wall of the nasal cavity. In general, this cavity is similar in *Thrinaxodon*, *Chiniquodon*, *Prozostrodon*, and *Riograndia*, but being deeper in *Riograndia* than in the others (Fig. 5c). In *Brasilodon* this structure is narrower laterally than in the other studied taxa and the morphology is conservative among different specimens (i.e., UFRGS-PV-929-T, UFRGS-PV-1030-T, and UFRGS-PV-1043-T) of the same genus. A similar structure in the palatine was identified in *Morganucodon* by Kermack et al.^81^ as the sphenopalatine sinus of the posterior nasal chamber, and may correspond to the most central region of the maxillary sinus (see “Discussion”).

In the palatal view, *Thrinaxodon*, *Chiniquodon*, *Prozostrodon*, and *Brasilodon* present the ventral edge of the secondary choana transversely rectilinear, whereas *Riograndia* presents a posterior projection (Fig. 6). This projection is convex, posteriorly rounded and is present in all analysed specimens of *Riograndia* (UFRGS-PV-596-T, UFRGS-PV-788-T, UFRGS-PV-833-T, MCN-PV 2264, UNISINOS 4881; see Figs. S4–S7). The projection is only in the most ventral portion of the palatine, not making contact with the lateral portion of the choana, similar to a platform. Furthermore, the crest that contacts the vomer, present on the floor of the nasal cavity, does not extend over to this projection. In *Riograndia*, the crest and the vomer diverge in the posterior portion of the nasopharyngeal duct and form a “V”-shaped notch.

#### Vomer

The general condition of the vomer of non-mammaliaform cynodonts is an anteroposteriorly elongated flat bone, which gives it a keel shape^31^ (Fig. 7). Anteriorly the vomer contacts the palatine process of the premaxilla, ventrally contacts the palatal processes of the maxilla and the palatine and posteriorly the vomer contacts the transverse process of the palatine (tpp—Fig. 4). Ventrally, the vomer contacts the secondary palate (formed by the palatal processes of the maxilla and palatine) through the ridge formed on the sagittal line by the contact between the palatals process of the two maxillaries and the palatine.

In addition, the vomer divides the ventral region of the nasal cavity, including the nasopharyngeal duct. In *Thrinaxodon*, the vomer is exposed at the opening of the secondary choana. The dorsal surface of the vomer is arched, in lateral view, gradually decreasing in height posteriorly (Fig. 7). Dorsally the vomer shows a groove for the cartilaginous attachment along its length, until to the anterior edge of the transverse lamina (Figs. 4, 7). Posteriorly, this sulcus converges medially into a crest that runs posteriorly along the rest of the length of the vomer. In *Thrinaxodon*, *Chiniquodon*, *Prozostrodon*, *Riograndia*, and *Brasilodon* (UFRGS-PV-1043-T) the groove runs the entire length of the vomer, starting at the contact of the premaxilla to the roof of the nasopharyngeal duct (Figs. 4, 6). In the smaller individuals of *Brasilodon* UFRGS-PV-929-T and UFRGS-PV-1030-T, the groove does not reach the anterior border of the vomer, being restricted to the posterior two-thirds.

Anteriorly, at the level of the incisive foramen, the vomer contacts between the two branches of the palatine process of the premaxilla (pp). Ventrally in this region, the paraseptal shelf of the vomer (psv) is dorsoventrally flat lamina, continues the shelf present in the palatine process of the premaxilla in all taxa. In *Prozostrodon*, *Riograndia*, and *Brasilodon* this shelf is formed by the vomer and premaxilla, and is more developed than in *Thrinaxodon*. This flat region is noted by Wible et al.^84^, Hillenius^75^, and Crompton et al.^31^ as a support area for the vomeronasal organ. Also, Ruf et al.^29^ marked a cavity inside the vomer of *Brasilodon* UFRGS-PV-1043-T as the structure that would accommodate the vomeronasal organ. This cavity in *Brasilodon* was mistakenly interpreted as part of the vomer but corresponds to the most posterior part of the palatine process of the premaxilla and is located anteriorly to the paraseptal shelf of the vomer.

In *Thrinaxodon*, *Prozostrodon*, *Riograndia*, and *Brasilodon* the paraseptal shelf of the vomer (psv) is rounded anteriorly and reduced gradually to the rear (Figs. 4, 7). In *Thrinaxodon* the paraseptal shelf is lateromedially more reduced (Fig. 7a) than in *Prozostrodon*, *Riograndia*, and *Brasilodon* (Fig. 7b–d). Probably due to a shorter snout, *Riograndia* has the posterior portion of the paraseptal shelf reduced too, but more abruptly than in *Thrinaxodon*, *Prozostrodon*, and *Brasilodon* (Figs. 4, 6). Furthermore, due to this condition, the posterior ends of the paraseptal shelf ends at the level of the anterior portion of the vomerine plate in dorsal view, in *Riograndia* (Fig. 7).

The vomerine plate is a dorsoventrally flattened lamina, positioned at the dorsal region of the vomer. This structure forms the transverse lamina together with the transverse process of the palatine (tpp) that partially overlaps the vomerine plate (vpl) (Fig. 4). The transverse lamina forms the roof of the nasopharyngeal duct and divides the respiratory region of the nasal cavity (below the transverse lamina) to the olfactory region (above the transverse lamina). In *Riograndia*, the anterior border of the vomerine plate starts at the level of the posterior end of the paraseptal shelf of the vomer, and expands laterally gradually in the anteroposterior direction (Fig. 4c). This condition is different to that of *Thrinaxodon*, *Prozostrodon*, and *Brasilodon* in which the anterior border of the vomerine plate starts at the level of the maxillary recess (mrs) and expands laterally gradually, forming the anteromedial border of the transverse lamina (Fig. 4a,b,d). In *Thrinaxodon*, this anterior border forms a 90° angle, in dorsal view, whereas in *Prozostrodon* and *Brasilodon* the angle is greater than 90° in dorsal view.

In *Thrinaxodon*, *Prozostrodon*, and *Brasilodon*, the vomer forms the anteromedial border of the transverse lamina with an elevation in this region of the vomerine plate (vpl—Fig. 4a,b,d). This elevation is articulated with a notch present in the transverse process of the palatine. In *Riograndia*, there is no elevation in the anterior border of the vomerine plate (vpl) that is completely overlapped by the transverse process of the palatine (tpp), not being visible in dorsal view. In *Prozostrodon*, this elevation is rectangular in dorsal view and more expanded anteroposteriorly than in *Thrinaxodon* and *Brasilodon*. The anterior elevation of the vomerine plate is rounded in the specimen UFRGS-PV-543-T of *Prozostrodon* (see Kerber et al.^79^), differing from the condition seen in the holotype UFRGS-PV-248-T.

In general, the posterior portion of the vomerine plate is dorsoventrally flat and is almost covered dorsally by the transverse process of the palatine, except the medial region (Fig. 4). The vomerine plate has a ‘rounded shovel’ shape with the lateral expansion of the lateral border that forms a concave ventral region. In *Riograndia* and *Brasilodon* the concavity is shallow, whereas in *Prozostrodon* and *Thrinaxodon* it is deeper. Also, the concavity in *Thrinaxodon* is "V"-shaped in cross section being less accentuated in *Prozostrodon*.

Posteriorly to the paraseptal shelf, the vomer becomes a vertical flat lamina that contacts a dorsal crest in the palatal region of the maxilla in *Prozostrodon*, *Riograndia*, and *Brasilodon*. In *Thrinaxodon*, due to the open secondary osseous palate, the ventral lamina of vomer does not contact the maxilla. Also, the ventral lamina starts to reduce its size at the level of the contact of the maxilla with the palatine and disappears completely at the level of the anterior edge of the transverse lamina. In *Riograndia* the ventral lamina reduces in size gradually, posteriorly, and disappears completely in the middle of the vomerine plate. At the level of the anterior edge of the transverse lamina, the maxillary crest reduces in size and diverges from the ventral crest of the vomer. In *Brasilodon* the ventral lamina of the vomer is similar to *Prozostrodon* and has the posterior portion abruptly reduced, anterior to the level of the transverse lamina. In addition, the vomer extends more posteriorly in the specimen UFRGS-PV-1043-T of *Brasilodon* than in UFRGS-PV-1030-T. In the ventral surface of the vomerine plate, another crest emerges in the sagittal plane, and runs at the end of the vomer. In the specimen UFRGS-PV-1043-T the ventral crest present in the ventral surface of the vomerine plate is tallest than in UFRGS-PV-1030-T.

#### Lacrimal

The lacrimal bone participates in the formation of the posterior region of the lateral wall of the nasal cavity (Fig. 5). In all analysed taxa, the lacrimal meets the nasal anterodorsally and the maxilla anteroventrally. The contact with the palatine is made by two ventral projections in *Thrinaxodon*, whereas in *Prozostrodon*, *Riograndia*, and *Brasilodon*, the ventral contact occurs along the entire lacrimal length (Figs. 5, S3). In addition, in *Riograndia*, and *Brasilodon*, the lacrimal meets the palatine posteroventrally. In the dorsal region of *Thrinaxodon* and *Prozostrodon*, probably because this region is less ossified than in the other, the lacrimal joints the prefrontal. However, in *Prozostrodon* the lacrimal also meets the frontal, posterior to the contact of the prefrontal. In *Riograndia* and *Brasilodon* the lacrimal contacts the frontal posterodorsally.

The lacrimal forms the entire border of the anterior opening of the lacrimal duct in *Thrinaxodon* whereas in *Riograndia* and *Brasilodon*, the lacrimal forms only the posterior border, which is positioned more medially in relation to the lateral wall. In *Prozostrodon* the anterior opening of the lacrimal duct is entirely formed by the maxilla. In *Thrinaxodon* and *Riograndia* the anterior opening of the lacrimal duct faces anteroventrally, whereas in *Brasilodon* it faces anterodorsally, and is in a more ventral position than in *Thrinaxodon* and *Riograndia*.

### Secondary choana

The secondary choana is formed by the palatine, ventrally and laterally, and dorsally by the vomer. In *Prozostrodon* and *Brasilodon*, the posterior end of the vomer that contacts the pterygoid cannot be determined due to the state of preservation of the fossils. Therefore, it is not possible to claim whether the pterygoid would contribute to the choana. Nonetheless, in specimens such as CAPPA/UFSM 0210 *Prozostrodon* the vomer extends posteriorly to the level of the secondary osseous palate^72^.

In palatal view, the ventral border of the choana does not reach the level of the last postcanine tooth in *Thrinaxodon*, differing from the condition of *Chiniquodon*, *Prozostrodon*, *Riograndia* and *Brasilodon* where the choana does reach the last postcanine. In *Riograndia*, the posterior border of the palatal region of the palatine, that forms the ventral border of the choana, presents a convex posterior projection. In *Thrinaxodon*, the dorsal border of the primary choana (the anterior border of the transverse lamina) is positioned at the same level of the border of the secondary choana (i.e., the posterior edge of the incomplete secondary palate). This condition exposes the interior of the nasal cavity through the secondary choana, in the ventral view of the palate.

The posterior portion of the crest of the vomer is exposed ventrally in the choana in *Thrinaxodon*. In *Chiniquodon*, *Prozostrodon*, *Riograndia*, and *Brasilodon*, the crest of the vomer contacts the palatal region of the palatine through the dorsal crest present in the sagittal line, and reduces gradually before the opening of the secondary choana, at the level of the anterior border of the transverse lamina.

## Discussion

### Nasal cavity

#### Nasal ridges and foramina

The topology of the nasal cavity roof is similar in *Thrinaxodon*, *Chiniquodon*, *Prozostrodon*, *Riograndia*, and *Brasilodon*. All taxa present two ridges that form a groove in the ventral surface of the nasal and run through its entire length to culminate in the region of the olfactory bulbs. These structures are similar to the condition reported for the early-diverging mammaliaform *Morganucodon*^81^. Kermack et al.^81^ described in *Morganucodon* two parallel longitudinal ridges in the ventral surface of the nasal that extended to the ventral surface of the frontal, and named them the ethmoid ridge (the medial) and the lateral ridge (the lateral). Furthermore, between these two ridges there is a groove named as the ethmoid vessel that is perforated by three foramina. These same ridges were identified on the ventral surface of the nasal and frontal of *Procynosuchus*^19^, *Nythosaurus*^39^, *Galesaurus*^36^, *Thrinaxodon*^19,78^, and *Diademodon*^19^, forming a groove between them. All these structures are identified as for blood vessels or nerves^81^. Ruf et al.^29^ and Laaß and Kaestner^40^ identified this groove as for the olfactory nerve; however, Angielczyk et al.^85^ interpreted it as the ophthalmic canals of the trigeminal nerve. Due to the grooves formed by the ethmoidal and lateral ridges are divided into branches anteriorly that end in the posterior border of the external nares would support the hypothesis that these grooves housed the olfactory nerve, as proposed by others^29,40^.

In *Didelphis virginiana* the lateral ridge of the nasal has a similar position to the semicircular lamina present in the ventral surface of the nasal. In *Thrinaxodon*, *Chiniquodon*, *Prozostrodon*, *Riograndia*, and *Brasilodon* the lateral ridge of the nasal (lrn) is well developed in comparison with the ethmoid ridge (ethr), and is projected medioventrally, similar to the semicircular lamina in *Didelphis*. In this cynodonts the lateral ridges form a constriction of the nasal cavity, delimiting a narrower dorsal region in relation to the more ventral region of the nasal cavity. This condition is similar in *Didelphis*^82^.

**Figure 8:**
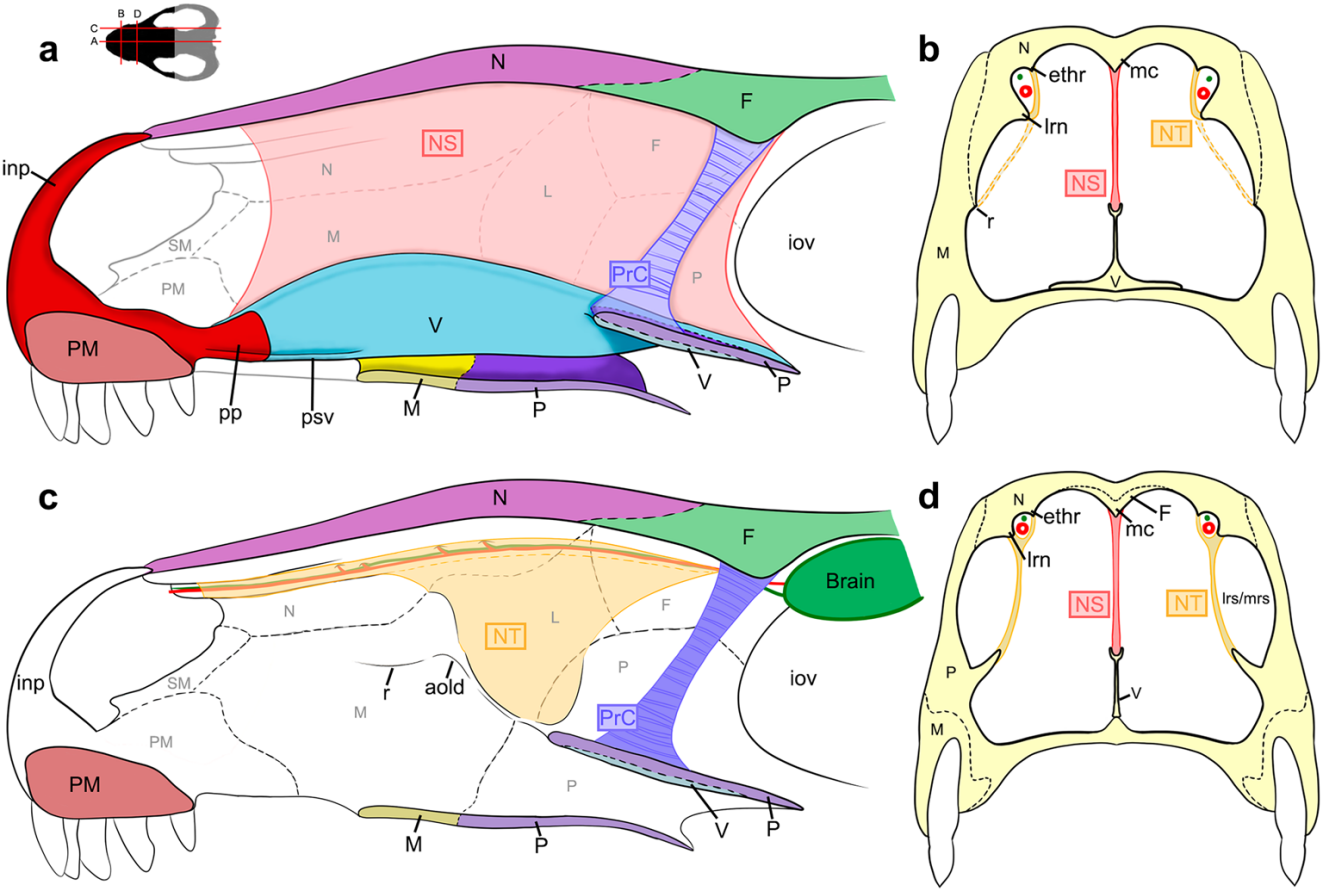
Schematic diagram of a sectioned non-mammaliaform cynodont skull, reconstructing the position of cartilaginous structures inside the nasal cavity based on new interpretations. (**a**) Section on the sagittal plane in lateral view. (**b**) Anterior cross-section of the skull, in anterior view. (**c**) Section in parasagittal plane in lateral view. (**d**) Posterior cross-section of the skull, in anterior view. Black dashed lines represent bone sutures. Yellow dashed lines represent a possible continuation of the nasoturbinal in *Riograndia* (see discussion). The red lines in (**c**) and red circles in (**b**–**d)** represent blood vessels. The green lines in C and green circles in (**b**–**d**) represent nerves. aold, anterior opening of lacrimal duct; ethr, ethmoid ridge of nasal; F, frontal; inp, internarial process of premaxilla; L, lacrimal; lrn, lateral ridge of nasal; lrs/mrs, lateral recess/maxillar recess; M, maxilla; mc, median crest; N, nasal; NS, nasal septum; NT, nasoturbinal; iov, orbital vacuity; P, palatine; PM, premaxilla; pp, palatine process of the premaxilla; PrC, “proto” cribriform plate; psv, paraseptal shelf of the vomer; r, ridge; SM, septomaxilla; V, vomer.

The function of the lateral and ethmoid ridges of the nasal cavity was identified by several authors as an anchoring structure for the turbinals in non-mammaliaform cynodonts^8,19,29,31,40,73,79^. However, it was not discussed which of these crests would have this function. In *Didelphis* the semicircular lamina anchors the anterior part of the nasoturbinal^26,64^ (as in most mammals^64,86^), and probably anchors a similar turbinal in non-mammaliaform cynodonts, as proposed by several authors^19,29,31^. However, the ethmoid ridge, which is more medially placed and reduced in size in comparison with the lateral ridge, does not show an analogous crest in *Didelphis*. Therefore, we believe that the ethmoid ridges cannot be associated with the turbinals. Instead, we observe that in *Thrinaxodon*, *Chiniquodon*, *Prozostrodon*, *Riograndia*, and *Brasilodon* the ethmoid ridges form a continuous groove that runs the entire length of the nasal cavity, starting at the opening of the external nostril and culminating at the limit between the nasal cavity and the encephalic cavity. In addition, this groove bears several foramina along its length, varying in number according to the species (see Nasal section). In *Galesaurus* it is possible to observe the presence of similar foramina in the groove formed by the crests of the ventral surface of the nasal^39^. This same groove is present in *Morganucodon*, and Kermack et al.^81^ proposed that the trough formed between the ridges could accommodate blood vessels or nerves, which would branch out into the exterior of the nasal through the foramina present in the groove. These vascular and/or nervous structures would be associated with the irrigation of possible cartilaginous turbinals, with the olfactory sense^23^, with respiratory function^23^ (e.g. moisture loss and air filtration), and also the vascularization and innervation of a fleshy snout (rhinarium) in *Morganucodon*^81^. We agree with Kermack et al.^81^ and we hypothesize that the lateral ridge of the nasal is likely linked to the presence of a turbinal, probably analogous to the nasoturbinal, whereas the ethmoid ridges would be more associated with the formation of the groove (Fig. 8). Furthermore, probably the groove is closed by the soft tissues that form the turbinal (Fig. 8) and olfactory epithelium. As aforementioned and also suggested by other authors^29,40^, the groove of the ethmoid ridges would housed the olfactory nerve and hold an olfaction function^28^of the non-ossified nasoturbinals.

The presence of these ridges, the lateral and ethmoid ones, in the inner walls of the nasal cavity is reported for several different groups of synapsids, with different topologies that were interpreted as being related to the attachment of turbinals (namely the nasoturbinal)^19,29,31,34,39,40,67,73,81,87–89^. Current studies that have taken into account other indirect thermophysiological evidence^40,42,46,53,59,60,90–92^ suggest that the endothermy appeared in cynodonts and maybe dicynodonts (depending on the proxy employed to determine the presence of endothermy), arising independently in these two clades. Concerning non-mammaliaform cynodonts and mammaliaforms, there is no consensus on the time of origin of endothermy. For example, Newham et al.^59^ (see also reference^60^) demonstrated that *Morganucodon* would have a metabolism closer to reptiles than to crown mammals, while Araújo et al.^42^ proposed the emergence of endothermy earlier, within the non-mammaliaform Eucynodontia clade, in the last common ancestor between *Pseudotherium* and other prozostrodonts. If we take into account these two hypotheses, we can consider that, if turbinals were present in the most basal branches of the synapsids (as advocated, for example, by several authors^19,87–89^), they probably would not be related to the endothermy, as discussed by Newham et al.^59^ and Martinez et al.^64^. Thus, the presence of turbinals precedes their role in endothermy since they are present in cynodonts putatively ectotherms as well as in endotherms^42^. This would be plausible since, in extant mammals, turbinals have functions that vary according to the type of cell present in the tissues that cover them, in addition to the two main functions (control of heat and moisture loss)^23,64^. Therefore, the presence of turbinals could be more associated with the control of moisture loss and air filtration in the more basal synapsids, since these lineages lived in warmer periods (Permian and Triassic) under low oxygen conditions on Earth^93^. Moreover, Martinez et al.^64^ point out that in extant mammals there is no direct evidence that corroborates the association of turbinals (maxilloturbinals) with metabolic rates, associating complexity and development of turbinals with multifactorial issues.

Associated with this condition, the grooves present in the ventral surface of the nasal of *Thrinaxodon*, *Chiniquodon*, *Prozostrodon*, *Riograndia*, and *Brasilodon*, support a large-diameter vascular system that would extend through the entire nasal cavity reaching the brain. This vascular system could help to cool the brain, similar to the vascular system present in the nasal cavity in modern mammals^94,95^. According to Taylor and Lyman^94^, the vascular system present in the nasal cavity of cursorial animals in desert environments, such as gazelles, acts as a way of cooling the brain and preventing damage to the structures in this region. Therefore, a vascular system in the nasal cavity of the studied cynodonts (i.e., *Thrinaxodon*, *Chiniquodon*, *Prozostrodon*, *Riograndia*, and *Brasilodon*) could have initially emerged as a brain cooling system, preceding the emergence of this functionality in turbinals or helping less complex (or efficient) turbinals to control heat loss. An efficient brain cooling system is an effective response to the hot and dry environment that prevailed in the Triassic period^93,96–99^. Furthermore, the presence of the grooves in the roof of the nasal cavity in presumably non-endothermic synapsids, as well as in different synapsid taxa that are endothermic^42,90–92^, would support the hypothesis that these vascular structures are more associated with responses to a hot environment than to endothermy, since they appear in taxa that presumably are not endothermic (sensu Araújo et al.^42^). Associated with this, an efficient brain cooling system would allow the development of larger brains^100^, corroborating published hypotheses of brain development in non-mammaliaform cynodonts^26,47,58,101–104^.

It is interesting to note that the semicircular lamina of *Didelphis virginiana* has a groove, related to the olfactory nerve, that runs through the entire length of the nasal cavity, starting at the external nostril and culminating in the region of the olfactory bulb^26,82^, similar to the condition in *Thrinaxodon*, *Chiniquodon*, *Prozostrodon*, *Riograndia*, and *Brasilodon*. However, in addition to the dimensions of the non-mammaliaform cynodonts trough being wider compared to that of *Didelphis*, in the former taxa the trough branches into two or more troughs in the anterior region (see Results). Furthermore, if turbinal anchorage occurs in the lateral ridge of the nasal, as in *Didelphis*, this extensive trough structure could delimit a lateral recess in *Thrinaxodon*, *Chiniquodon*, *Prozostrodon*, *Riograndia*, and *Brasilodon,* as well as in extant mammals (see^23^). This hypothesis is supported by the general morphology of the nasal cavity, since the lateral ridge of the nasal is more marked anteriorly at the beginning of the expansion of the nasal cavity and runs through its entire length, to the transverse crests (Fig. 2). The lateral crest lies medially on the skull in relation to the expansion of the nasal cavity, and would probably support the turbinals that would enclose the inner wall of the lateral recess. If this condition reflects the presence of the lateral recess, and the embryological development of non-mammaliaform cynodonts was similar to that of modern mammals, the pars lateralis would be present, contrary to Ruf et al.^29^. Furthermore, the concave structure present in the palatine bone, located lateral to the opening of the nasopharyngeal duct, ventrally delimits the maxillary sinus, which is also present in extant mammals^19,64^ and therefore would also be present in *Thrinaxodon*, *Chiniquodon*, *Prozostrodon*, *Riograndia*, and *Brasilodon*.

It is important to note that, on the most ventral surface of the descending process of the nasal of *Riograndia* UFRGS-PV-596-T, there is a weakly marked ridge, parallel to the lateral ridge of the nasal. This ridge could be analogous to the ridge where the maxillary turbinals are anchored in *Didelphis*, helping to anchor different turbinals, or even the same turbinals that anchor on the lateral ridge of the nasal. However, since this ridge was not found in any other specimen of *Riograndia*, nor in the other analysed taxa, this ridge could be an effect of the taphonomic processes, since it is located in the rearmost portion of the bone, or even an artefact of the CT scans.

#### Ossification of the nasal cavity in *Brasilodon*: turbinals or no turbinals?

**Figure 9:**
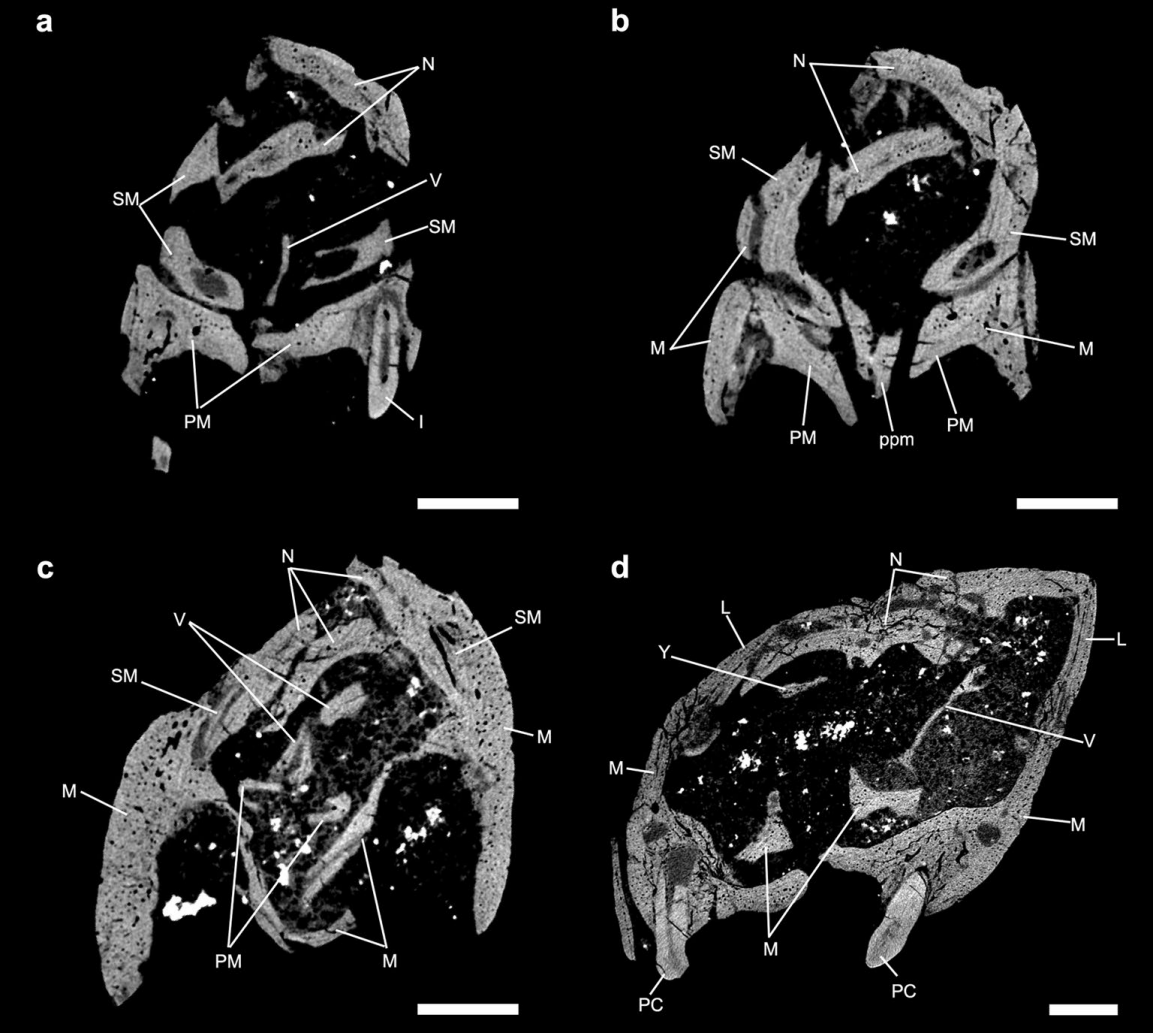
*Brasilodon quadrangularis* (UFRGS-PV-1043-T). (**a–d**) Transverse section slice of the nasal region of *Brasilodon*. The position of each slice follows the sections present in the Fig. 3a of Ruf et al.^29^. Scale bar equals 1 mm. I, upper incisive; L, lacrimal; M, maxilla; N, nasal; PC, upper postcanine; PM, premaxilla; ppm, palatal process of the premaxilla; SM, septomaxilla; V, vomer; Y, structure identified as a nasoturbinal by Ruf et al.^29^, here interpreted as a fragment of the descending process of the nasal.

**Figure 10:**
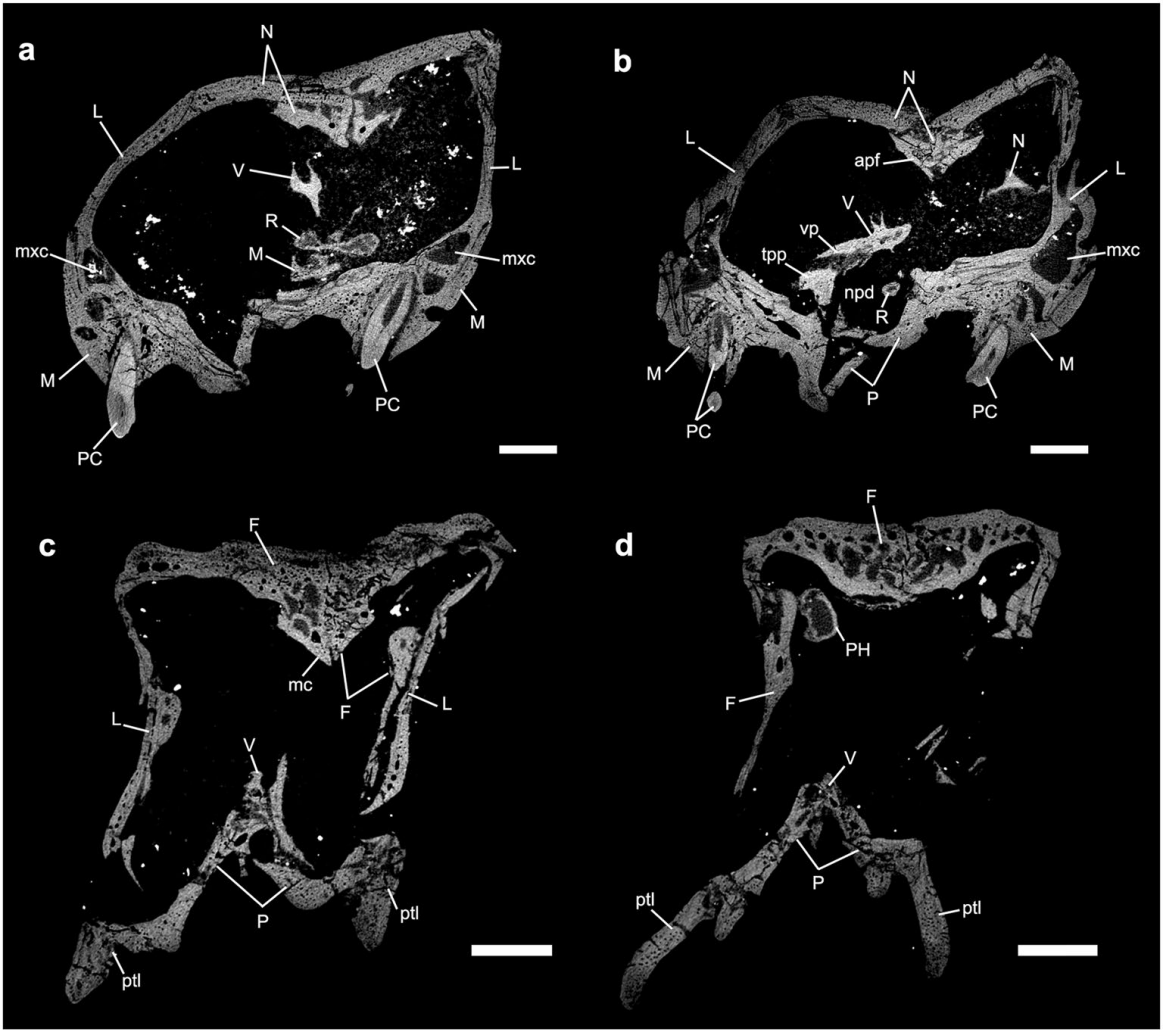
*Brasilodon quadrangularis* (UFRGS-PV-1043-T). (**a–d**) Transverse section slice of the nasal region. The position of each slice follows the sections present in the Figs. 4 and 5 of Ruf et al.^29^. Scale bar equals 1 mm. apf, anterior projection of the frontal; F, frontal; PH, phalanx; L, lacrimal; M, maxilla; mc, median crest; mxc, maxillary canal; N, nasal; npd, nasopharyngeal duct; P, palatine; PC, upper postcanine; ptl, lateral flange of pterygoid; R, ribs; tpp, transverse process of the palatine; V, vomer; vp, ventral projection.

In the description of the nasal cavity of *Brasilodon* (UFRGS-PV-1043-T), Ruf et al.^29^ identified some structures as ossified nasoturbinals, ethmoturbinals, and part of the nasal septum. The fragment identified as an ossified nasal septum by Ruf et al.^29^ corresponds to a small fragment of a laminar bone (figs. 3a and 10a,c in reference^29^). This structure is located in the anterior region of the nasal cavity that is very damaged in the specimen UFRGS-PV-1043-T. Furthermore, the vomer presents fractures along its length, and damaged regions, and it is not possible to determine the origin of this fragment. However, we observe in the three-dimensional model of UFRGS-PV-1043-T that the dorsal groove reaches the anterior portion of the vomer, until contact with the premaxilla (Fig. 3). The dorsal groove indicates the presence of an unossified nasal septum, covering the entire length of the nasal cavity, to the most anterior region. This condition is observed in all analysed non-mammaliaform cynodonts (see Description and Fig. 3). Furthermore, the ossification of the nasal septum in extant mammals occurs from the posterior towards the anterior region, remaining there cartilaginous^24–26^. Therefore, we believe that the fragment previously identified as ossified nasal septum by Ruf et al.^29^ corresponds to a fragment of the vomer (Fig. 9a), and the nasal septum remains cartilaginous in the adult stages of all non-mammaliaform cynodonts.

The two structures identified by Ruf et al.^29^ (figs. 3d, 7, 9) as the nasoturbinals are a small fragment located in the left side of the skull, and a fragment of a flattened bone positioned in the right side of the skull, both inside the sedimentary matrix present inside the nasal cavity. The small fragment is a part of the ventral portion of the medial crest of the nasal. The flatted bone presents some foramina in the anterior portion and the general shape is similar to a lamina that has the medial border wider than the lateral one. Furthermore, the descendent process of the nasal is broken and the shape of the isolated bone is similar to the missing part of that bone. Therefore, we agree with Crompton et al.^31^ that the structure presented by Ruf et al.^29^ is not a nasoturbinal. We believe that the structure is actually a fragment of the nasal, being the smaller part of the medial crest and the large one part of the descendent process of the nasal. By its turn, the ethmoturbinal identified by Ruf et al.^29^ (i.e., figs. 4b, 5a) is located in a region of the skull that presents the lateral wall fragmented (Fig. 9), probably from the descendent process of the nasal. In our interpretation, this structure is a ventral part of the descendent process of the frontal (Fig. 10c). This region is also damaged by taphonomic processes, as well as the other fragments; however, it clearly shows the continuity of the dorsal region of the frontal in the other slices. In addition to the turbinals, Ruf et al.^29^ identified two structures as possible hyoids (Fig. 5b in Ruf et al.^29^), which are actually two phalanxes (Fig. 10d).

Despite identifying ossified cartilaginous structures as the turbinals, Ruf et al.^29^ pointed out that the boundary of the nasal cavity and brain cavity remains unossified in *Brasilodon* UFRGS-PV-1043-T. We agree with this hypothesis, since no bone structures were found from secondary ossifications, as happens in living mammals^24–26^, and we observe the presence of a ventral crest and a ventral projection in the roof of the nasal cavity, probably related to the anchorage of the cartilaginous structures. In addition, we note that the crest and ventral projection is much more developed in taxa closer to mammaliaforms, such as *Brasilodon*, than in other earlier cynodonts (Fig. 2), and it is also present in the earliest mammaliaforms, such as *Morganucodon* (see^81^).

#### The medial crest and frontal projection

**Figure 11:**
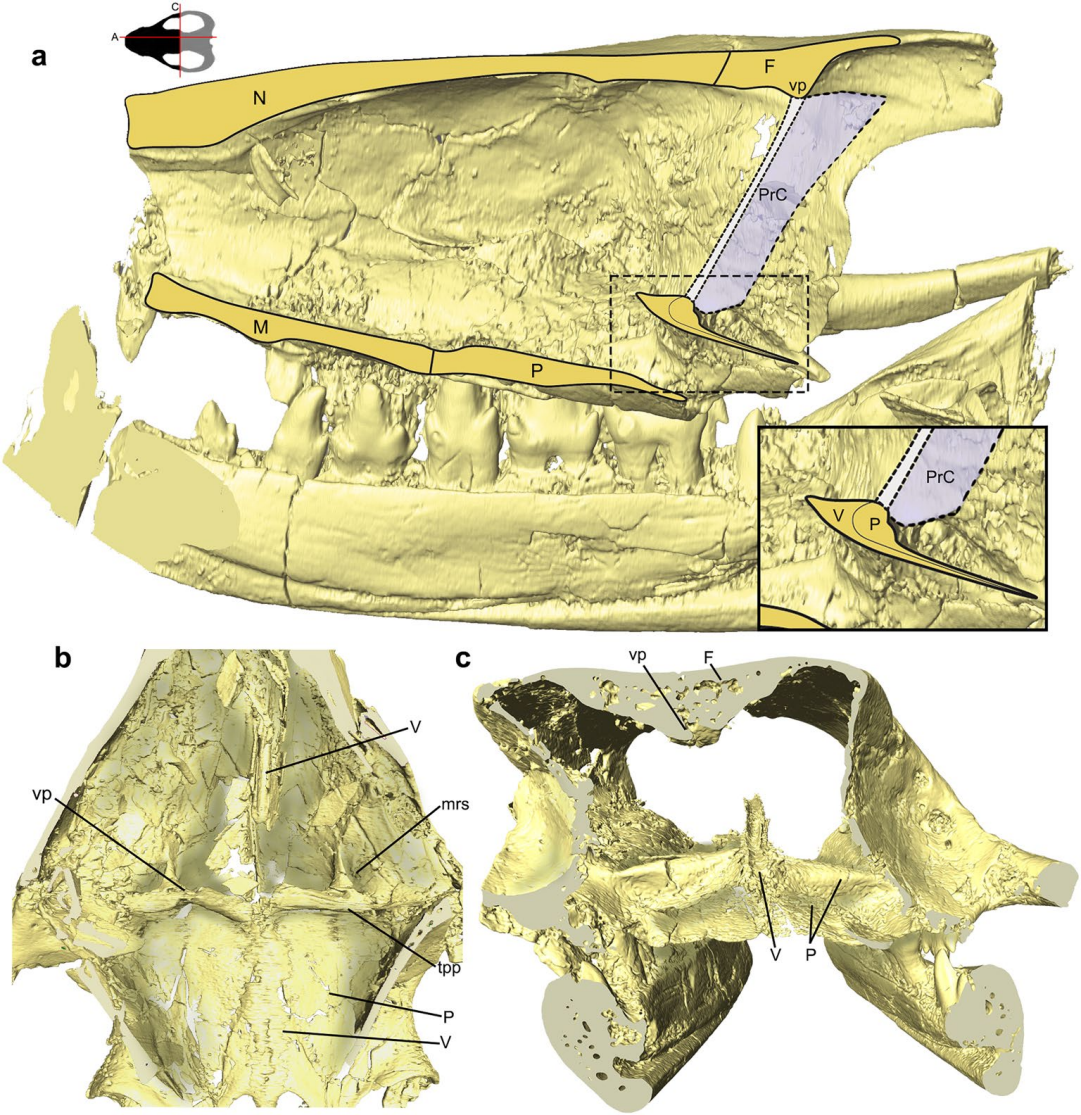
Sections of the skull of *Brasilodon quadrangularis*. (**a**) Right half of the skull in lateral view of UFRGS-PV-1030-T, exhibiting the lateral wall of the nasal cavity and the scheme of the “proto” cribriform plate. (**b**) Skull section in dorsal view of UFRGS-PV-929-T, exhibiting the dorsal region of the floor of the nasal cavity. (**c**) Skull section in posterior view of UFRGS-PV-1030-T, exhibiting the posterior region of the ventral projection of the frontal and the dorsal surface of nasopharingeal duct (primary choane). F, frontal; M, Maxilla; mrs, maxillary recess; N, Nasal; P, palatine; PrC, “proto” cribriform plate; V, Vomer; vp, ventral projection; tpp, transverse process of the palatine.

In this re-analysis and segmentation of the specimen UFRGS-PV-1043-T, used in Ruf et al.^29^, and other *Brasilodon* specimens (e.g., UFRGS-PV-929-T, UFRGS-PV-1030-T), as well as the other specimens (e.g., *Riograndia*, *Prozostrodon*, *Chiniquodon*, and *Thrinaxodon*) it is clear that the structure pointed out by Ruf et al.^29^ as a possible mesethmoid is, actually, the posteriormost portion of the median crest of the frontal bone. In this area, the crest forms a ventral projection that is triangular in cross-section. This ventral projection is formed by cancellous bone, differing from the adjacent regions (e.g., the descending palatal process of the frontal) that are formed by laminar bone, with compact internal tissues (Fig. 10c). This structure is present in other non-mammaliaform cynodonts as was indicated by Crompton et al.^31^, as well as in new specimens described here (see Description section). Furthermore, according to Broom^24^ (see also^26,105^) the mesethmoid is a dorsal and posterior ossification of the cartilage of the nasal septum, present in extant mammals, which ossifies during the prenatal phase. In the new tomography of the specimen UFRGS-PV-1043-T there is no evidence of a secondary ossification. Crompton et al.^31^ attributed the composition of cancellous bone to the difficulty of identifying the suture between the frontals, which can hide secondary ossification points as well. Nonetheless, in the anterior portion of the anterior projection of the frontal, the suture between both frontals is present and gradually disappears posteriorly (Figs. 2, 11, 13). Furthermore, in the anterior portion of the frontal projection, the separation of the frontal and nasal is clear, disagreeing with the proposal of Crompton et al.^31^ of the participation of the nasal in the formation of the ventral projection of the frontal.

Despite being present in other taxa besides *Brasilodon*, the median crest and the ventral projection have different morphologies among non-mammaliaform cynodonts. In earlier diverging species, such as *Thrinaxodon*, this structure is more reduced or less marked, whereas in *Brasilodon* and *Riograndia* it is much deeper and pronounced. Noteworthy, a similar structure is described for *Morganucodon*, by Kermack et al.^81^, which proposed that in the early stage of evolution of the mammaliaforms (e.g., *Morganucodon*) the median ridge and the transverse crest attached the nasal cartilage (e.g., mesethmoid cartilage) which may or may not be ossified in *Morganucodon*. Thus, the presence of the median ridge, as well as the ventral projection and the well-marked transverse ridge in non-mammaliaform cynodonts, could serve as an anchor point for a structure that remains cartilaginous. This becomes more plausible when we observe in living mammals that the support structure for the bones of the nasal septum is flat, and does not present a sharp crest as in non-mammaliaforms cynodonts (see^82^).

**Figure 12:**
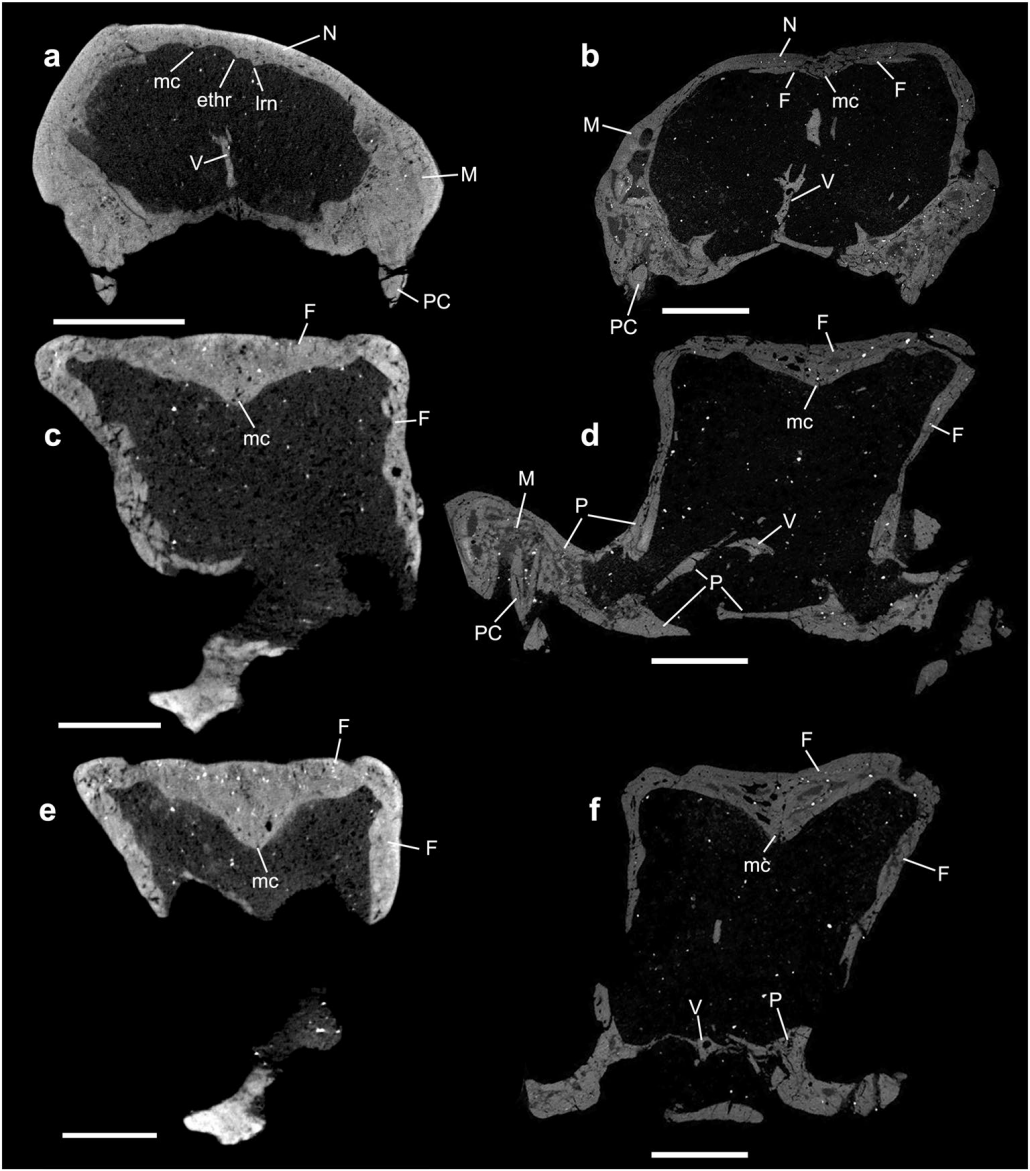
*Riograndia guaibensis*. (**a**–**f**) Transverse section slice of the nasal region of holotype MCN-PV 2264 (**a**, **c**, **e**) and UFRGS-PV-596-T (**b**, **d**, **f**). ethr, ethmoid ridge of nasal; F, frontal; lrn, lateral ridge of nasal; M, maxilla; mc, median crest; N, nasal; P, palatine; PC, upper postcanine; V, vomer; vp, ventral projection. Scale bar 2.5 cm.

Even though Ruf et al.^29^ and Wallace et al.^33^ identify possible ossifications inside the nasal cavity of *Brasilodon* and *Pseudotherium*, respectively, we believe that these structures do not correspond to ossifications of the nasal cavity cartilages, but to artefacts of fossilization. Furthermore, Kerber et al.^73^ did not find any evidence of ossifications in *Prozostrodon* CAPPA/UFSM 0210. The general condition observed in non-mammaliaform cynodonts is the same in the internal structures of the nasal cavity, observed in different specimens of the same species (e.g., *Riograndia* UFRGS-PV-1043-T and MCN-PV 2264) (Fig. 12) even in individuals of the same species with different ontogenetic stages (e.g. *Brasilodon* UFRGS-PV-929-T and UFRGS-PV-1043-T). This proposal is supported by records of ethmoid bones and cribriform plates restricted to the Gondwanatheria and Multituberculata^106–109^, as well as a dubious record for Docodonta^109,110^. Furthermore, in living mammals, the ossification of the nasal septum cartilage occurs in a period prior to birth^24,26^. Therefore, the ossification of these structures should be present in all the specimens analysed here since they all represent characteristics of more advanced ontogenetic stages^111^.

#### What do these data mean?

**Figure 13:**
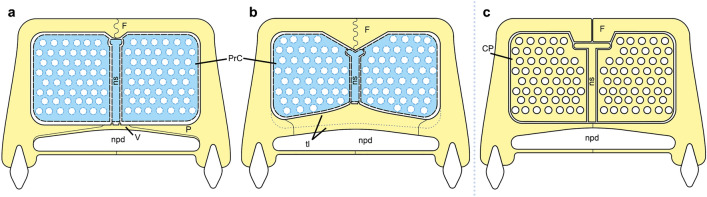
Schematic diagram of morphological patterns during the evolution of the ventral projection of the frontal and the dorsal crest of the transverse lamina. (**a**) A basal probainognathian, in which the ventral crest of the frontal is small and the transverse lamina (tl) is shallow and does not have a dorsal projection. (**b**) *Brasilodon*/*Morganucodon* in which the ventral crest of the frontal is well developed and there is a dorsal projection on the deeper transverse lamina. (**c**) The pattern found in extant mammals (e.g., *Didelphis virginiana*). In light blue are marked the structures that remain cartilaginous and in yellow the structures that ossify. CP, cribriform plate; F, frontal; npd, nasopharyngeal duct; ns, nasal septum; P, palatine; PrC, “proto” cribriform plate; tl, transverse lamina; V, vomer.

The configuration of the ventral projection of the nasal roof in non-mammaliaform cynodonts suggests at least two different evolutionary pulses in this region, if related to what is observed in extant mammals (Fig. 13). The condition of the first pulse is observed in non-mammaliaform cynodonts: the frontal projects gradually form the ventral process (i.e., ventral projection of the frontal), forming a callus, that is evidently more developed in more advanced non-mammaliaform cynodonts (e.g., *Brasilodon*) than in early diverging prozostrodonts (e.g., *Prozostrodon* and *Riograndia*) (Figs. 2, 4). As cited by Crompton et al.^31^, this ventral projection is also present in the traversodontid *Massetognathus* but it appears to be smaller than in non-mammaliaform probainognathian cynodonts. Furthermore, the frontal projects ventral and anteriorly below the ventral surface of the nasal, and forms a sagittal ridge that connects with the ventral projection. This anterior projection is also more developed in more advanced non-mammaliaform cynodonts. In *Brasilodon*, the projection of the frontal exhibits its most development among non-mammaliaform cynodonts, being larger than *Riograndia* and *Prozostrodon*. Once the nasal septum remains cartilaginous throughout the life of the non-mammaliaform cynodonts, the presence of the ridge and callus works as a support for nasal septum cartilage anchorage, that divides the nasal cavity of cynodonts.

The condition of the nasal septum in extant mammals is different from that observed in non-mammaliaform cynodonts. As mentioned by Broom^24^, in mammals the nasal septum is completely ossified before the birth of the individual. Therefore, the condition of the nasal septum attachment is different from the non-mammaliaform cynodonts, and this pattern corresponds to the second evolutionary pulse. In mammals (here represented by *Didelphis virginiana*^82^), it is observed that the ventral surface of the frontal and nasal has a reduced structure to connect the ossified nasal septum. In the ventral surface of the nasal are observed two conditions in the contact of the nasal cavity with the ossified nasal septum. The anterior portion of the nasal, with the nasal forming more of the roof of the nasal cavity than the frontal, has a tiny sagittal ridge in its ventral surface. In the posterior portion of the nasal, where the frontal forms the greater part of the roof of the nasal cavity, the ventral surface is flattened. The ventral surface of the frontal presents a ventral projection that is smaller than that of *Brasilodon*, and has a flattened surface in the contact with the nasal septum. In this surface there is a tiny rugosity. Therefore, the condition present in current mammals suggests that during the transition of non-mammaliaform cynodonts to current mammals, the ventral projection of the frontal reduces in size when the nasal septum ossified, and this reduction corresponds to the second evolutionary pulse we propose here. This becomes evident since the frontal projection is more developed in non-mammaliaform cynodonts which do not have an ossified nasal septum and absent/or reduced compared to extant mammals, which present ossification of the nasal septum. Of course, the vast diversity of Mesozoic mammaliaform lineages^7,112,113^ may further indicate a much more complex evolutionary scheme and will enable us to explore the morphological disparity of the nasal cavity within the millions of years of evolution between non-mammaliaform cynodonts and therian mammals is out of the scope of the present contribution.

### The lateral wall of the nasal cavity

Several literatures associate the ridge present in the anterodorsal edge of the anterior opening of lacrimal duct with the maxilloturbinal anchorage (e.g.,^19,39,80^). However, there is a large morphological variation in the taxa analyzed here when compared with the literature. In *Riograndia*, the transverse process of the palatine (tpp) extends laterally, forming a shelf which gradually narrows until it reaches the ventral border of the opening of the lacrimal duct (anterior medial opening of the lacrimal duct in Kermack et al.^81^), inside the nasal cavity. This shelf is aligned with what appears to be a ridge present in the most ventral region of the descending process of the nasal, present only on the right side of the skull. We consider that this ridge is an effect of taphonomic processes in *Riograndia*. In *Brasilodon*, a similar ridge runs along the entire lateral wall, at the level of the first postcanine until close to the lateral border of the transverse process of the palatine. This ridge is formed by the maxilla, anteriorly, and the lacrimal, posteriorly, and is the contact surface of the descending process of the nasal.

The opening of the lacrimal duct into the nasal cavity, in all analysed non-mammaliaform cynodonts, including *Prozostrodon* and *Thrinaxodon*, is facing forward and downward, and the posterior dorsal border of this opening is aligned with the lateral border of the transverse process of the palatine, which in turn forms the ridge in the lateral wall. Due to this condition, we hypothesize that the posterodorsal border of the opening of the lacrimal duct, inside the nasal cavity, participates of the ridge, being the most anterior limit of the ridge in taxa such as *Prozostrodon* and *Thrinaxodon*. This lateral ridge is at the same level as the anterior edge of the vomerine plate, in the dorsal region of the lateral face of the vomer. This ridge extends anteriorly at the same level as the ridge present on the lateral wall of nasal cavity. These two ridges (in the lateral wall and the vomerine plate) are likely to be attachment points for cartilaginous tissue, and the presence of these two ridges demarcate the boundary of the division of the olfactory region from the respiratory region of the nasal cavity (Fig. 4).

It is important to note that, Pusch et al.^36^ identified a small ridge anterior to the opening of the lacrimal duct in *Galeasaurus* and associated this structure with the anchorage of the maxilloturbinal. We did not observe the presence of a similar ridge in this region in *Thrinaxodon*, *Prozostrodon*, and *Brasilodon*, except for a small projection on the right lateral wall of *Riograndia* UFRGS-PV-596-T. Therefore, we cannot confirm that this elevation is a ridge and is associated with the maxilloturbinal since it is only found on one side of the *Riograndia* skull; may be the effect of taphonomy.

### The vomer and anchoring of the nasal septum

In the dorsal region of the vomer, the presence of the groove is a strong indication that the nasal septum remained cartilaginous in all non-mammaliaform cynodonts (Figs. 5, 9). Associated with this, the presence of the medial crest on the roof of the nasal cavity (Figs. 2, S1) corroborates this hypothesis. Although the presence of the dorsal groove in the vomer occurs along the entire length of the vomer in *Thrinaxodon*, *Chiniquodon*, *Prozostrodon*, *Riograndia*, and *Brasilodon*, in the smaller individuals of *Brasilodon* (UFRGS-PV-929-T and UFRGS-PV-1030-T) the groove is absent in the anterior portion. In the specimen UFRGS-PV-929-T the anterior dorsal region of the vomer is broken, which makes this condition doubtful. However, in the specimen UFRGS-PV-1030-T the vomer appears to be complete and has no fractures in this region, which leads us to believe that this condition is an ontogenetic variation. A similar condition is reported in *Probainognathus jenseni* by Crompton et al.^31^, which pointed to the presence of the vomer dorsal groove only in the posterior two thirds. However, we do not rule out the possibility that both in *Brasilodon* UFRGS-PV-929-T and UFRGS-PV-1030-T and in *Probainognathus jenseni*^31^ this region is altered due to taphonomic issues.

Posterior to the groove, all studied taxa present a crest originating from the junction of the walls of the groove medially, which runs along the entire length of the vomer up to contact with the pterygoid. The presence of this crest may be associated with the continuity of the cartilages of the nasal septum that divide the nasal cavity. If this structure is associated with a cartilage, it would be in a similar position to the crista galli of extant mammals^26^. This structure crosses the middle region of the cribriform plate and may or may not be visible posterior to it (Figs. 5, 8), varying according to the species in current mammals^26^. In addition, this structure can anchor some connective tissues in the brain^25,26^. In non-mammaliaform cynodonts, this structure may serve as a support point for the cartilages of the cribriform plate, and may be associated with the cartilages of posterior regions of the nasal cavity, such as the cartilages present in the interorbital vacuity.

The paraseptal shelf of the vomer supports the vomeronasal organ in marsupials^25,26^. In non-mammaliaform cynodonts, Wible et al.^84^, Hillenius^75^ and Crompton et al.^31^ agreed that the shelf present in the anterior region (here called the paraseptal shelf) would support the same structure as in living marsupials. Therefore, we believe that the paraseptal shelf is homologous between non-mammaliaform cynodonts and extant mammals, and probably, as in marsupials, would support the vomeronasal organ. Nonetheless, Ruf et al.^29^ pointed to an inner cavity in the ventral region of the vomer as the cavity where the vomeronasal organ is located. However, we believe that this cavity is an artefact of the taphonomic processes, once this cavity was not observed in any other specimen of *Brasilodon* or in the other species analyzed. We agree with the other authors about the position of this structure.

### The roof of the nasopharyngeal duct

Crompton et al.^31^ use the terminology “transverse lamina” to refer to the roof of the nasopharyngeal duct (nasopharyngeal passage), which also corresponds to the floor of the olfactory cavity, in *Massetognathus*, *Probainognathus*, *Elliotherium*, and *Morganucodon*. This structure is formed by the vomerine plate and palatine flange^31^, and is also present in *Thrinaxodon*, *Prozostrodon*, *Riograndia*, and *Brasilodon*. However, for extant mammals, the terminology “posterior transverse lamina” refers to the same region of the nasopharyngeal duct, where it is formed by the cartilages of the nasal capsule, with or without contribution from the vomer and palatine bones^23,26^, and may present the participation of ethmoturbinals (see discussion in^23,64^). Therefore, we believe that the structures that form the roof of the nasopharyngeal duct in non-mammaliaform cynodonts and extant mammals are not homologous. For this reason, we use the same nomenclature as Crompton et al.^31^.

Even so, the topology and function of the transverse lamina is similar in non-mammaliaform cynodonts and mammals, separating the olfactory from the respiratory region. Furthermore, the dorsal region of the transverse lamina corresponds to the most posterior region of the nasal cavity and was probably filled by structures similar to the turbinal and cribriform plate in non-mammaliaform cynodonts, as in extant mammals as described by Macrini^26^. If this interpretation is true, the cribriform plate and turbinals, although not ossified, would probably be well developed. Added to that, the presence of the ventral projection of the medial crest and the transverse crest on the ventral surface of the frontal reinforces the presence of a structure that would separate the nasal cavity from the rest of the braincase. This structure would probably be more developed in more derived forms, such as *Riograndia* and *Brasilodon*, where the frontal projection is more prominent, than in *Thrinaxodon*, *Prozostrodon*, and probably *Chiniquodon* where this structure is reduced (Fig. 3). Furthermore, the folding of the anterior edge of the transverse lamina in *Brasilodon* is probably related to an anchoring reinforcement of the cartilaginous structures such as a turbinal and the “proto” cribriform plate itself.

It is worth mentioning that in mammals^25,26^, the transverse lamina is an analogous structure, as presented by Hillenius^19^ and Crompton et al.^31^. In mammals the posterior transverse lamina is a structure formed by the paraseptal shelf of the vomer (also called the turbinovomerine lamina) and part of the endoturbinals (ethmoid plate) and has no participation in the palatine (*sensu*^25,26^), whereas in the non-mammaliaform cynodonts the transverse lamina is a structure formed by the vomerine plate and palatine flanges (transverse processes of the palatine) (*sensu*^31^). Despite this difference, both structures are present in the same position, and divide the olfactory region (dorsal to the transverse lamina) of the nasal cavity to the respiratory region (ventral to the transverse lamina)^25,26,31^. The condition presented by Crompton et al.^31^ is the same condition observed in *Thrinaxodon*, *Chiniquodon*, *Prozostrodon*, *Riograndia*, and *Brasilodon*.

### The evolution of the secondary choana

The conditions presented for the choana in the sampled taxa demonstrate the changes undergone during the process of closing and extending of the secondary palate. In *Thrinaxodon*, the secondary choana is ventrally facing and the primary palate (transverse lamina) is more oblique in relation to the secondary bony palate. With the closure of the secondary bony palate, the vomer contacts the maxilla and palatine, dividing the nasopharyngeal duct, and the posterior portion of the secondary bony palate develops posteriorly until it reaches the posterior level of the postcanine row, as seen in *Chiniquodon*, *Prozostrodon*, *Riograndia*, and *Brasilodon* (Figs. 10c,d, 12a,b).

In *Thrinaxodon*, the primary palate exhibits a lateral depression in the palatine, and the posterior portion of the vomer (and probably the anterior portion of the pterygoid) is facing downwards in dorsal view, which makes the secondary choana more concave than in other cynodonts. In *Chiniquodon*, *Prozostrodon*, and *Riograndia*, the same depression and vomer curvature is present, but very reduced, being more marked in *Prozostrodon*. In *Brasilodon*, the primary palate does not present a depression, but the transverse lamina is gently concave in ventral view. With the development of the secondary bony palate, the primary palate (transverse lamina) becomes more parallel to the secondary bony palate, causing the bony part of the nasopharyngeal duct to become more elongated and the secondary choana to face more posteriorly.

**Figure 14:**
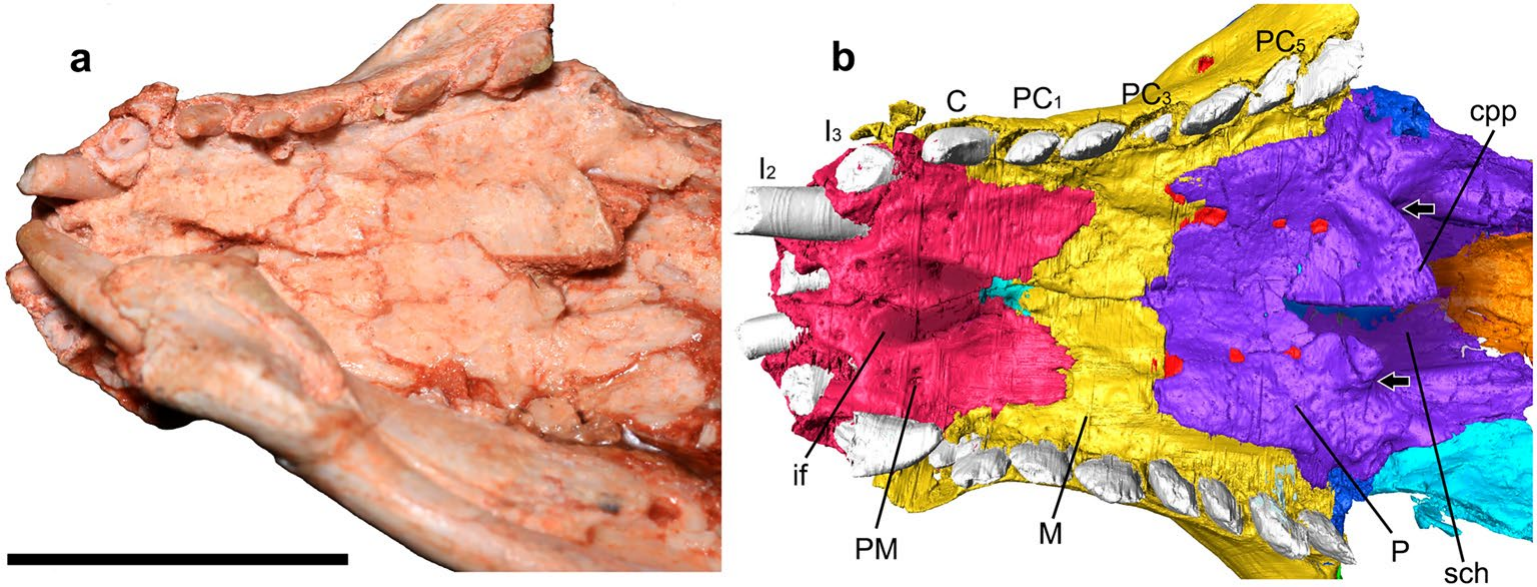
Ventral view of the palatal region of the *Riograndia* UFRGS-PV-596-T. (**a**) Photograph of the palatal region of the specimen, with the right mandible in occlusion. (**b**) Model generated through CT scan of the specimen, showing the palatal region. Black arrows indicate the notch in the lateral of the posterior projection of palatine. C, upper canine; cpp, convex posterior projection of palatine; I, upper incisive; if, incisive foramen; M, maxilla; P, palatine; PC, upper postcanine; PM, premaxilla; sch, secondary choana.

It is important to note that, although the secondary choana of *Riograndia* reaches the posterior level of the postcanine row, the anatomy of the ventral border of the secondary choana is different from that of *Prozostrodon*^72^ and *Brasilodon*. In *Riograndia* (UFRGS-PV-596-T), the posterior portion of the left palatine is well preserved, showing a convex posterior projection (Figs. 6, 14). When complete, the posterior border of the secondary bony palate has a triangular shape, differing from the straight border present in *Prozostrodon* and *Brasilodon* (Fig. 6). This configuration in *Riograndia* forms a notch in the palatine between the posterior border of the secondary palate and its laterodorsal process (Figs. 6, 14b, notch indicated with arrows). In *Chiniquodon*, the posteroventral border of the secondary choana is straight as in *Prozostrodon* and *Brasilodon*; however, it presents a tiny notch in the lateral face, similar to *Riograndia* but reduced. This anatomical difference in the ventral border of the secondary choana (i.e., the posterior border of the secondary bony palate) could indicate that the development of the secondary bony palate happens independently in different lineages within probainognathians, adopting different evolutionary strategies among taxa.

The secondary choana in the five studied taxa illustrates an evolutionary sequence of this structure in non-mammaliaform cynodonts. In *Thrinaxodon* the ventral border of the secondary choana is more anteriorly placed than the other taxa. Furthermore, the opening of the secondary choana is turned ventrally when compared to other cynodonts, which have the choana turned more posteriorly, as well as the dorsal edge of the primary choana is positioned at the same level as the ventral edge of the secondary choana. Thus, the ossified part of the nasopharyngeal duct is more reduced in *Thrinaxodon*. In *Chiniquodon*, *Prozostrodon*, *Riograndia*, and *Brasilodon* the nasopharyngeal duct is more elongated and both edges of the choanae are not at the same level.

## Conclusion

In this contribution we provide new information on the cranial anatomy (rostrum, nasal cavity, palate, as well as its inner structures) of five species of non-mammaliaform cynodonts using µCT-scan images, providing a comparative overview of its organization at different phylogenetic levels within Cynodontia. One of the main observations of the nasal cavity of non-mammaliaform cynodonts here analysed is the absence of ossified structures inside the nasal capsule, such as turbinals and nasal septum, contrary to the proposals of Ruf et al.^29^ for *Brasilodon*. The structures identified as fragments of ossified regions are parts of the adjacent snout bones that fragmented during the fossilization process. The structure identified as mesethmoid by Ruf et al.^29^ corresponds to the most posterior portion of the median crest of the ventral surface of the frontal. It is evident in the new CT scans that this structure is entirely formed by the frontal bone and does not show signs of participation in secondary ossifications. This condition is present in all analysed taxa (*Thrinaxodon*, *Prozostrodon*, *Riograndia* and *Brasilodon*); however, is more developed in *Brasilodon* than in the others. Therefore, we believe that the structures originating from the cartilages inside the nasal cavity (nasal capsule) remain cartilaginous throughout the individuals lifespan, in all non-mammaliaform cynodont taxa as suggested by Crompton et al.^31^. These cartilages probably only ossified later in mammaliaform groups, since *Morganucodon*^81^ presents a topology similar to that of non-mammaliaform probainognathians, such as *Brasilodon,* and there are no records of these ossified structures in that taxon.

Nonetheless, we do not exclude the presence of non-ossified structures similar to the turbinals in non-mammaliaform cynodonts. The ridges present on the lateral and dorsal walls of the nasal cavity are possible anchor points of turbinals, as proposed in the literature^19,28–40^. However, we believe that not all of these ridges are associated with the anchorage function of turbinals. In all analysed taxa, but mainly in *Riograndia* and *Brasilodon*, the lateral ridge (ventral surface of the nasal) presents a topology similar to the semicircular lamina of extant mammals (*Didelphis virginiana*), which anchors the nasoturbinal. However, the ethmoid ridges, which lie medially to the lateral ridge, probably do not have the same function as it is shorter than the lateral ridge, has a more medial position and is much less developed than the lateral ridge. We associate this crest with the ventral nasal groove that would have a vascular and/or nervous function, associated with the olfactory and respiratory stimuli. We believe that this groove is responsible for the vascularization of soft tissues that covered the cartilaginous turbinals and is probably a key part of the brain's cooling function, as in some extant mammals^94,95^, since it runs along the entire length of the nasal cavity and culminates in the cephalic region. Furthermore, this structure has a large dimension, which would allow a high level of irrigation through the blood vessels, and we do not find analogous structures in living mammals. Therefore, we hypothesize that ossified turbinals could not be fully developed in non-mammaliaform cynodonts or in the first mammaliaforms as happens in current mammals (with scrolled and branched turbinals), or if present it does not have a thermoregulatory function. With higher metabolism in taxa more derived than non-mammaliaform cynodonts^42^, or even within mammaliaforms^59,60^, a simpler system of cooling the brain through blood vessel could be plausible. The development of these structures for cooling the brain may be linked to the development of the brain throughout the cynodont lineage.

The ventral projection of the frontal is positioned between the nasal cavity anteriorly and the brain cavity posteriorly in all analysed taxa. We believe that this structure anchors the cartilages of the nasal septum, together with the medial crest, and is a similar structure to the cribriform plate of modern mammals. In *Brasilodon*, the ridge formed by the fold of the anterior edge of the transverse lamina also would be associated with the anchorage of cartilage that closes the posterior region of the nasal cavity, analogous to the cribriform plate. We cannot state that this structure would be a direct precursor of the cribriform plate, but it is clear that this region is much more developed in *Brasilodon* than in other non-mammaliaform cynodonts, and *Brasilodon* is recovered as the sister taxon of mammaliaforms in most current phylogenetic hypotheses^71–73,114–116^. Furthermore, we observe that the topology of the roof of the nasal cavity of *Morganucodon*^81^ is very similar in *Brasilodon* and, despite not having a specimen with the transverse lamina well preserved; we believe that the anchoring structures are similar. Therefore, we observed an increase in the complexity of this region (medial crest, transverse crest, transverse lamina) in non-mammaliaform cynodont lineages closer to mammaliaforms. This also reinforces the idea that these structures (e.g., turbinals, “proto” cribriform plate) remain cartilaginous in non-mammaliaform cynodonts, and therefore present a very different topology from that found in modern mammals. In addition, the presence of turbinals in other synapsids^40^ would not necessarily be associated with temperature control or the development of endothermy. We therefore believe that the ridges present in the nasal cavity of other synapsids should be reassessed to understand its homologies.

In addition, we emphasize the importance of considering the quality and resolution of CT scans when interpreting the data, since poor resolution can hide structures, or even modify the interpretation of them. We also highlight the importance of editing the CT scans, which allows us to analyse the structures from a different angle than conventional orthogonal slices, as well as retrodeformation of material and structures (although this does not rule out the importance of using CT slices as a way of obtaining data). We believe that the internal structures of the nasal cavity can help with taxonomic questions, in addition to bringing information about the anatomy and physiology of fossil forms, as well as information from the brain and ear structures.

## Methods

### Analysed fossil specimens

A total of 10 specimens belonging to five species of non-mammaliaform cynodonts are studied, as follows: four specimens of *Riograndia guaibensis* (UFRGS-PV-596-T, UFRGS-PV-788-T, UFRGS-PV-833-T, MCN-PV 2264-holotype-, and UNISINOS-4881), three of *Brasilodon quadrangularis* (UFRGS-PV-1043-T, UFRGS-PV-929-T, and UFRGS-PV-1030-T), the holotype of *Prozostrodon brasiliensis* (UFRGS-PV-248-T), and one specimen of *Chiniquodon theotonicus* (NHMUK PV R8429) described by Kemp^44^ [this specimen was originally referred to as *Probelesodon lewisi*, a taxon erected by Romer^117^ that was latter synonymized as a junior synonym of *Chiniquodon theotonicus* by Abdala and Giannini^118^]. *Thrinaxodon liorhinus* is represented by one specimen (NHMUK PV R511). Specimens UFRGS-PV-1043-T and UFRGS-PV-929-T were originally referred to *Brasilitherium riograndensis*^29,69,104,119,120^ but this taxon was synonymized with *B. quadrangularis*^98,111,121–124^. UFRGS-PV-1030-T was referred to *Minicynodon maieri*^111,112^ but is now considered a juvenile of *B. quadrangularis*^123,124^. The CT scan of *Didelphis virginiana* was taken from Macrini^82^, available in the DigiMorph.org database. Additional images of the described specimens are provided in Supplementary Data 1, as well as 3D videos of the rostrum of *Thrinaxodon*, *Prozostrodon*, *Riograndia*, and *Brasilodon*.

### Location and stratigraphic data of fossil specimens

The specimen UFRGS-PV-0596-T and MCN-PV 2264 are from Sesmaria do Pinhal 1 outcrop, Candelária municipality, state of Rio Grande do Sul, Brazil, and the specimens UFRGS-PV-788-T, UFRGS-PV-0833-T, UFRGS-PV-929-T, UFRGS-PV-1030-T, UFRGS-PV-1043-T and UNISINOS-4881 are from Linha São Luiz outcrop, Faxinal do Soturno municipality, state of Rio Grande do Sul, Brazil. Both localities belong to the early Norian *Riograndia* Assemblage Zone (AZ), of the Candelária Sequence, Santa Maria Supersequence^98^. Specimen UFRGS-PV-248-T is from an outcrop on the BR-216 road, near to the outcrop Cerrito, Santa Maria city, state of Rio Grande do Sul, Brazil, from the late Carnian *Hyperodapedon* AZ, of the Candelária Sequence^90^. The specimen NHMUK PV R8429 is from Chanãres River, Campo de Talampaya, La Rioja Province, Argentina, from the early Carnian Chañares Formation (Ischigualasto-Villa Unión Basin)^97^. The specimen NHMUK PV R511 is from the Early Triassic *Lystrosaurus* AZ of the Karoo Basin of Free State, South Africa^83^.

### Scanning procedures and three-dimensional modeling

The specimens UFRGS-PV-0929-T and UFRGS-PV-833-T were scanned with a μCT Phoenix High-Resolution X-Ray x|s Nanofocus, in the Institute of biotechnology, University of Helsinki, Finland, and the specimens UFRGS-PV-248-T, UFRGS-PV-596-T, UFRGS-PV-788-T, UFRGS-PV-1030-T, UFRGS-PV-1043-T, and UNISINOS-4881 were scanned with a μCT NIKON XTH225ST, in the School of Earth Sciences, University of Bristol, United Kingdom in the last five years. Specimens NHMUK PV R511 and NHMUK PV R8429 were scanned in a Zeiss Xradia Versa 520 machine at the National History Museum of London. The specimen MCN-PV 2264 was scanned with Bruker SkyScan 1173 at the Instituto de Petróleo e dos Recursos Naturais (Laboratório de Sedimentologia e Petrologia) of Pontifícia Universidade Católica do Rio Grande do Sul (PUCRS), Porto Alegre, Brazil. Additional data on the scans is provided in Supplmentary Data 1. The slices were analyded and edited in Avizo (version 7.1, 8.1, and 9.1, Visualisation Sciences Group), through manual editing using a tablet One by WACOM. The specimens of *Riograndia* and *Prozostrodon* had the bones of the anterior region of the snout (premaxilla, septomaxilla, maxilla, nasal, frontal, vomer, palatine, and lacrimal) digitally separated. In the specimens of *Brasilodon* only the nasal, frontal, maxilla and palatine are digitally separated.

### Anatomical terminology

The anatomical nomenclature follows Broom^24^, Macrini^26^, and Crompton et al.^31^ for structures inside the nasal cavity and cartilaginous structures of the ethmoid bones. Other references used for nomenclature are Kermack et al.^81^, Hillenius^19,75^, Ruf et al.^29^ and Benoit et al.^14^.

### Supplementary Information

Supplementary Information 1.

Supplementary Information 2.

## Data Availability

Additional information, including figures and videos of the analysed specimens used in this study, is available in the Supplementary Information files.

## Abbreviations

NHMUK: Natural History Museum, London, UK
UFRGS: Universidade Federal do Rio Grande do Sul, Porto Alegre, Brazil
UNISINOS: Universidade do Vale do Rio dos Sinos, São Leopoldo, Rio Grande do Sul, Brazil
MCN: Museu de Ciências Naturais, Secretaria Estadual do Meio Ambiente e Infrastrutura, Porto Alegre, Rio Grande do Sul, Brazil

## References

[1] Rowe T (1988). Definition, diagnosis, and origin of Mammalia. J. Vertebr. Paleontol..

[2] Hopson JA, Kitching JW (2001). A probainognathian cynodont from South Africa and the phylogeny of nonmammalian cynodonts. Bull. Mus. Comp. Zool..

[3] Benson RJ (2012). Interrelationships of basal synapsids: Cranial and postcranial morphological partitions suggest different topologies. J. Syst. Paleontol..

[4] Burgin CJ, Colella JP, Kahn PL, Upham NS (2018). How many species of mammals are there?. J. Mammal..

[5] Rowe T (1996). Coevolution of the mammalian middle ear and neocortex. Science.

[6] Luo Z-X, Kielan-Jaworowska Z, Cifelli RL (2004). Evolution of dental replacement in mammals. Bull. Carn. Mus. Nat. Hist..

[7] Kielan-Jaworowska Z, Cifelli RL, Luo Z-X (2004). Mammals from the Age of Dinosaurs: Origins, Evolution, and Structure.

[8] Kemp TS (2005). The Origin and Evolution of Mammals.

[9] Heesy CP, Hall MI (2010). The nocturnal bottleneck and the evolution of mammalian vision. Brain Behav. Evol..

[10] Rowe TB, Macrini TE, Luo Z-X (2011). Fossil evidence on the origin of the mammalian brain. Science.

[11] Rowe TB, Shepherd GM (2016). Role of ortho-retronasal olfaction in mammalian cortical evolution. J. Comp. Neurol..

[12] Rowe T, Kaas J (2017). The emergence of mammals. Evolution of Nervous Systems.

[13] Benoit J, Manger PR, Rubidge BS (2016). Palaeoneurological clues to the evolution of defining mammalian soft tissue traits. Sci. Rep..

[14] Benoit J, Ruf I, Miyamae JA, Fernandez V, Rodrigues PG, Rubidge BS (2020). The evolution of the maxillary canal in Probainognathia (Cynodontia, Synapsida): Reassessment of the homology of the infraorbital foramen in mammalian ancestors. J. Mammal. Evol..

[15] Norton LA, Abdala F, Benoit J (2023). Craniodental anatomy in Permian–Jurassic Cynodontia and Mammaliaformes (Synapsida, Therapsida) as a gateway to defining mammalian soft tissue and behavioural traits. Philos. Trans. R. Soc. B.

[16] Bennett AF, Ruben JA, Hotton N, Maclean PD, Roth JJ, Roth EC (1986). The metabolic and thermoregulatory status of therapsids. The Ecology and Biology of Mammal-Like Reptiles.

[17] Hopson JA (2012). The role of foraging mode in the origin of therapsids: Implications for the origin of mammalian endothermy. Fieldiana.

[18] Knaus PL, van Heteren AH, Lungmus JK, Sander PM (2021). High blood flow into the femur indicates elevated aerobic capacity in synapsids since the Synapsida-Sauropsida split. Front. Ecol. Evol..

[19] Hillenius WJ (1994). Turbinates in therapsids: Evidence for late Permian origins of mammalian endothermy. Evolution.

[20] Moore WJ (1981). The Mammalian Skull.

[21] Van Valkenburgh B, Smith TD, Craven BA (2014). Tour of a labyrinth: Exploring the vertebrate nose. Anat. Rec..

[22] Smith TD, Eiting TP, Bhatnagar KP, Doty RL (2015). Anatomy of the nasal passages in mammals. Handbook of Olfaction and Gustation.

[23] Martinez Q (2023). Turbinal bones are still one of the last frontiers of the tetrapod skull: Hypotheses, challenges and perspectives. PaleorXiv.

[24] Broom R (1927). Some further points on the structure of the mammalian basicranial axis. Proc. Zool. Soc. Lond..

[25] Rowe TB, Eiting TP, Macrini TE, Ketcham RA (2005). Organization of the olfactory and respiratory skeleton in the nose of the gray short-tailed opossum *Monodelphis domestica*. J. Mammal. Evol..

[26] Macrini TE (2012). Comparative morphology of the internal nasal skeleton of adult marsupials based on X-ray computed tomography. Bull. Am. Mus. Nat. Hist..

[27] Kemp TS (1979). The primitive cynodont *Procynosuchus*: Functional anatomy of the skull and relationships. Philos. Trans. R. Soc. Lond. B.

[28] Sigurdsen T (2006). New features of the snout and orbit of a therocephalian therapsid from South Africa. Acta Palaeont. Pol..

[29] Ruf I, Maier W, Rodrigues PG, Schultz CL (2014). Nasal Anatomy of the non-mammaliaform cynodont *Brasilitherium riograndensis* (Eucynodontia, Therapsida) reveals new insight into mammalian evolution. Anat. Rec..

[30] Crompton AW, Musinsky C, Owerkowicz T, Dial K, Shubin N, Brainerd E (2015). Evolution of the mammalian nose. Great Transformations in Vertebrate Evolution.

[31] Crompton AW, Owerkowicz T, Bhullar BA, Musinsky C (2017). Structure of the nasal region of non-mammalian cynodonts and mammaliaforms: Speculations on the evolution of mammalian endothermy. J. Vertebr. Paleontol..

[32] Bendel E-M, Kammerer CF, Kardjilov N, Fernandez V, Fröbisch J (2018). Cranial anatomy of the gorgonopsian *Cynariops robustus* based on CT-reconstruction. PLoS ONE.

[33] Wallace RV, Martínez R, Rowe T (2019). First record of a basal mammaliamorph from the early Late Triassic Ischigualasto Formation of Argentina. PLoS ONE.

[34] Franco AS, Müller RT, Martinelli AG, Hoffmann CA, Kerber L (2021). The nasal cavity of two traversodontid cynodonts (Eucynodontia, Gomphodontia) from the Upper Triassic of Brazil. J. Paleontol..

[35] Huttenlocker AK, Sidor CA (2020). A basal nonmammaliaform cynodont from the Permian of Zambia and the origins of mammalian endocranial and postcranial anatomy. J. Vertebr. Paleontol..

[36] Pusch LC, Kammerer CF, Fröbisch J (2019). Cranial anatomy of the early cynodont *Galesaurus planiceps* and the origin of mammalian endocranial characters. J. Anat..

[37] Pusch LC, Ponstein J, Kammerer CF, Fröbisch J (2020). Novel endocranial data on the early therocephalian *Lycosuchus vanderrieti* underpin high character variability in early theriodont evolution. Front. Ecol. Evol..

[38] Pusch LC, Kammerer CF, Fröbisch J (2021). Cranial anatomy of *Bolotridon frerensis*, an enigmatic cynodont from the Middle Triassic of South Africa, and its phylogenetic significance. PeerJ.

[39] Pusch LC, Kammerer CF, Fröbisch J (2024). The origin and evolution of Cynodontia (Synapsida, Therapsida): Reassessment of the phylogeny and systematics of the earliest members of this clade using 3D-imaging technologies. Anat. Rec..

[40] Laaß M, Kaestner A (2023). Nasal turbinates of the dicynodont *Kawingasaurus fossilis* and the possible impact of the fossorial habitat on the evolution of endothermy. J. Morphol..

[41] Grigg G, Nowack J, Bicudo JEPW, Bal NC, Woodward HN, Seymour RS (2022). Whole-body endothermy: Ancient, homologous and widespread among the ancestors of mammals, birds and crocodylians. Biol. Rev..

[42] Araújo R, David R, Benoit J, Lungmus JK, Stoessel A, Barrett PM (2022). Inner ear biomechanics reveals a Late Triassic origin for mammalian endothermy. Nature.

[43] Clarke A, Pörtner H-O (2010). Temperature, metabolic power and the evolution of endothermy. Biol. Rev..

[44] Lovegrove BG (2017). A phenology of the evolution of endothermy in birds and mammals. Biol. Rev. Camb. Philos. Soc..

[45] Legendre LJ, Davesne D (2020). The evolution of mechanisms involved in vertebrate endothermy. Philos. Trans. R. Soc. B.

[46] Olivier C, Houssaye A, Jalil N-E, Cubo J (2017). First palaeohistological inference of resting metabolic rate in an extinct synapsid, *Moghreberia nmachouensis* (Therapsida: Anomodontia). Biol. J. Linn. Soc..

[47] Jerison HJ (1971). More on why birds and mammals have big brains. Am. Nat..

[48] Taylor CR, Schmidt-Nielsen K, Bolis L, Taylor CR (1980). Evolution of mammalian homeothermy: A two-step process?. Comparative Physiology: Primitive Mammals.

[49] Crompton AW, Taylor CR, Jagger JA (1978). Evolution of homeothermy in mammals. Nature.

[50] Kemp TS (2006). The origin of mammalian endothermy: A paradigm for the evolution of a complex biological structure. Zool. J. Linn. Soc..

[51] Kemp TS (2009). The endocranial cavity of a nonmammalian eucynodont, *Chiniquodon theotenicus*, and its implications for the origin of the mammalian brain. J. Vertebr. Paleontol..

[52] Farmer CG (2020). Parental care, destabilizing selection, and the evolution of tetrapod endothermy. Physiology.

[53] Farmer CG (2000). Parental care: The key to understanding endothermy and other convergent features in birds and mammals. Am. Nat..

[54] Farmer CG (2003). Reproduction: The adaptive significance of endothermy. Am. Nat..

[55] Benoit J, Abdala F, Manger P, Rubidge B (2016). The sixth sense in mammalian forerunners: Variability of the parietal foramen and the evolution of the pineal eye in South African Permo-Triassic eutheriodont therapsids. Acta Palaeontol. Pol..

[56] Benoit J, Abdala F, Van den Brandt MJ, Manger PR, Rubidge BS (2015). Physiological implications of the abnormal absence of the parietal foramen in a late Permian cynodont (Therapsida). Sci. Nat..

[57] Benoit J, Manger PR, Norton LA, Fernandez V, Rubidge BS (2017). Synchrotron scanning reveals the palaeoneurology of the headbutting *Moschops capensis* (Synapsida, Dinocephalia). PeerJ.

[58] Benoit J, Dollman KN, Smith RM, Manger PR (2022). At the root of the mammalian mind: The sensory organs, brain and behavior of pre-mammalian synapsids. Progr. Brain Res..

[59] Newham E, Gill PG, Brewer P, Benton MJ, Fernandez V, Gostling NJ (2020). Reptile-like physiology in early Jurassic stem-mammals. Nat. Commun..

[60] Newham E, Gill PG, Corfe IJ (2022). New tools suggest a middle Jurassic origin for mammalian endothermy: Advances in state-of-the-art techniques uncover new insights on the evolutionary patterns of mammalian endothermy through time. BioEssays.

[61] Hu Q, Nelson TJ, Seymour RS (2020). Bone foramen dimensions and blood flow calculation: Best practices. J. Anat..

[62] Benton MJ (2021). The origin of endothermy in synapsids and archosaurs and arms races in the Triassic. Gondwana Res..

[63] Hu Q, Nelson TJ, Seymour RS (2021). Morphology of the nutrient artery and its foramen in relation to femoral bone perfusion rates of laying and non-laying hens. J. Exp. Biol..

[64] Martinez Q, Okrouhlík J, Šumbera R, Wright M, Araújo R, Braude S, Hildebrandt TB, Holtze S, Ruf I, Fabre PH (2023). Mammalian maxilloturbinal evolution does not reflect thermal biology. Nat. Commun..

[65] Parrington FR, Westoll TS (1940). On the evolution of the mammalian palate. Philos. Trans. R. Soc. Lond. B.

[66] Maier W, van den Heever J, Durand F (1996). New therapsid specimens and the origin of the secondary hard and soft palate of mammals. J. Zool. Syst. Evol. Res..

[67] Sidor CA, Smith RMH (2004). A new galesaurid (Therapsida: Cynodontia) from the lower Triassic of South Africa. Palaeontology.

[68] Hopson JA, Barghusen H, Hotton N, Maclean PD, Roth JJ, Roth EC (1986). An analysis of therapsid relationships. The Ecology and Biology of Mammal-Like Reptiles.

[69] Bonaparte JF, Martinelli AG, Schultz CL (2005). New information on *Brasilodon* and *Brasilitherium* (Cynodontia, Probainognathia) from the Late Triassic of southern Brazil. Rev. Bras. Paleontol..

[70] Soares MB, Schultz CL, Horn BLD (2011). New information on *Riograndia guaibensis* Bonaparte, Ferigolo & Ribeiro, 2001 (Eucynodontia, Tritheledontidae) from the Late Triassic of southern Brazil: Anatomical and biostratigraphic implications. An. Acad. Bras. Ciênc..

[71] Martinelli AG, Ezcurra MD, Fiorelli LE, Escobar J, Hechenleitner EM, von Baczko MB, Taborda JRA, Desojo JB (2024). A new early-diverging probainognathian cynodont and a revision of the occurrence of cf.
* Aleodon
* from the Chañares formation, northwestern Argentina: New clues on the faunistic composition of the latest Middle–?earliest Late Triassic
* Tarjadia
* Assemblage Zone. Anat. Rec..

[72] Stefanello M, Martinelli AG, Müller RT, Dias-da-Silva S, Kerber L (2023). A complete skull of a stem mammal from the Late Triassic of Brazil illuminates the early evolution of prozostrodontian cynodonts. J. Mammal. Evol..

[73] Kerber L, Roese-Miron L, Bubadué JM, Martinelli AG (2024). Endocranial anatomy of the early prozostrodonts (Eucynodontia: Probainognathia) and the neurosensory evolution of the mammalian forerunners. Anat. Rec..

[74] Martinelli AG, Kammerer CF, Melo TP, Paes Neto VD, Ribeiro AM, Da-Rosa AAS, Schultz CL, Soares MB (2017). The African cynodont *Aleodon* (Cynodontia, Probainognathia) in the Triassic of southern Brazil and its biostratigraphic significance. PLoS ONE.

[75] Hillenius WJ (2000). Septomaxilla of nonmammalian synapsids: Soft-tissuecorrelates and a new functional interpretation. J. Morphol..

[76] Brink AS (1955). A study on the skeleton of *Diademodon*. Palaeontol. Afr..

[77] Bonaparte JF (1966). Sobre las cavidades cerebral, nasal y otras estructuras del cráneo de *Exaeretodon* sp. (Cynodontia, Traversodontidae). Acta Geol. Lilloana.

[78] Fourie S (1974). The cranial morphology of *Thrinaxodon liorhinus*. Ann. S. Afr. Mus..

[79] Kerber L, Martinelli AG, Rodrigues PG, Ribeiro AM, Schultz CL, Soares M (2020). New record of *Prozostrodon brasiliensis* (Eucynodontia: Prozostrodontia) from its type-locality (Upper Triassic, southern Brazil): Comments on the endocranial morphology. Rev. Bras. Paleontol..

[80] Pusch LC, Kammerer CF, Fernandez V, Fröbisch J (2022). Cranial anatomy of *Nythosaurus larvatus* Owen, 1876, an Early Triassic cynodont preserving a natural endocast. J. Vertebr. Paleontol..

[81] Kermack KA, Mussett F, Rigney HW (1981). The skull of *Morganucodon*. Zool. J. Linn. Soc..

[82] Macrini, T. E. *Didelphis virginiana* (On-line), Digital Morphology. Accessed August 24, 2023; http://digimorph.org/specimens/Didelphis_virginiana (2005).

[83] Jasinoski SC, Abdala F, Fernandez V (2015). Ontogeny of the early Triassic cynodont *Thrinaxodon liorhinus* (Therapsida): Cranial morphology. Anat. Rec..

[84] Wible JR, Miao D, Hopson JA (1990). The septomaxilla of fossil and recent synapsids and the problem of the septomaxilla of monotremes and armadillos. Zool. J. Linn. Soc..

[85] Angielczyk KD, Benoit J, Rubidge BS (2019). A new tusked cistecephalid dicynodont (Therapsida, Anomodontia) from the upper Permian Madumabisa Mudstone Formation, Luangwa Basin, Zambia. Pap. Palaeontol..

[86] Ruf I (2020). Ontogenetic transformations of the ethmoidal region in Muroidea (Rodentia, Mammalia): New insights from perinatal stages. Vertebr. Zool..

[87] Cluver MA (1971). The cranial morphology of the dicynodont genus *Lystrosaurus*. Ann. S. Afr. Mus..

[88] Barry TH (1967). The cranial morphology of the Permo-Triassic anomodont *Pristerodon buffaloensis* with special reference to the neural endocranium and visceral arch skeleton. Ann. S. Afr. Mus..

[89] Romer AS, Price LW, Price LI (1940). Review of the Pelycosauria.

[90] Rey K (2017). Oxygen isotopes suggest elevated thermometabolism within multiple Permo-Triassic therapsid clades. eLife.

[91] Huttenlocker AK, Farmer CG (2017). Bone microvasculature tracks red blood cell size diminution in Triassic mammal and dinosaur forerunners. Curr. Biol..

[92] Faure-Brac MG, Cubo J (2020). Were the synapsids primitively endotherms? A palaeohistological approach using phylogenetic eigenvector maps. Philos. Trans. R. Soc. B.

[93] Mancuso AC, Horn BLD, Benavente CA, Schultz CL, Irmis RB (2021). The paleoclimatic context for South American Triassic vertebrate evolution. J. S. Am. Earth Sci..

[94] Taylor CR, Lyman CP (1972). Heat storage in running antelopes: Independence of brain and body temperatures. Am. J. Physiol. Leg. Content.

[95] Tada S, Tsuihiji T, Matsumoto R, Hanai T, Iwami Y, Tomita N, Hideaki S, Tsogtbaatar K (2023). Evolutionary process toward avian-like cephalic thermoregulation system in Theropoda elucidated based on nasal structures. R. Soc. Open Sci..

[96] Horn BLD, Goldberg K, Schultz CL (2018). A loess deposit in the Late Triassic of southern Gondwana, and its significance to global paleoclimate. J. S. Am. Earth Sci..

[97] Fiorelli LE, Rocher S, Martinelli AG, Ezcurra MD, Hechenleitner EM, Ezpeleta M (2018). Tetrapod burrows from the Middle−Upper Triassic Chañares Formation (La Rioja, Argentina) and its palaeoecological implications. Palaeogeogr. Palaeoclimatol. Palaeoecol..

[98] Schultz CL, Martinelli AG, Soares MB, Pinheiro FL, Kerber L, Horn BL, Pretto FA, Müller RT, Melo TP (2020). Triassic faunal successions of the Paraná Basin, southern Brazil. J. S. Am. Earth Sci..

[99] Corecco L, Pereira VP, Soares MB, Schultz CL (2020). Geochemical study of the vertebrate assemblage zones of the Santa Maria Supersequence (Middle to Late Triassic), Paraná Basin, Brazil. Braz. J Geol..

[100] Martin RD (1981). Relative brain size and basal metabolic rate in terrestrial vertebrates. Nature.

[101] Hulbert AJ, Schmidt-Nielsen K, Bolis L, Taylor CR (1980). The evolution of energy metabolism in mammals. Comparative Physiology: Primitive Mammals.

[102] Allman J (1990). The origin of the neocortex. Neurosciences.

[103] Allman J (2000). Evolving Brains.

[104] Rodrigues PG, Ruf I, Schultz CL (2013). Digital reconstruction of the otic Region and inner ear of the non-mammalian cynodont *Brasilitherium riograndensis* (Late Triassic, Brazil) and its relevance to the evolution of the mammalian ear. J. Mammal. Evol..

[105] Ferigolo J (1981). The mesethmoid bone and the Edentata. Anais Acad Bras. Ciênc..

[106] Hurum JH (1998). The braincase of two Late Cretaceous Asian multituberculates studied by serial sections. Acta Palaeontol. Pol..

[107] Macrini, T. E. The evolution of endocranial space in mammals and non-mammalian cynodonts. Ph.D. dissertation, 295 (University of Texas at Austin, Austin, Texas, 2006).

[108] Krause DW, Wible JR, Hoffmann S, Groenke JR, O’Connor PM, Holloway WL, Rossie JB (2014). Craniofacial morphology of *Vintana sertichi* (Mammalia, Gondwanatheria) from the Late Cretaceous of Madagascar. J. Vertebr. Paleontol..

[109] Hoffmann S, O’Connor PM, Kirk EC, Wible JR, Krause DW (2014). Endocranial and inner ear morphology of *Vintana sertichi* (Mammalia, Gondwanatheria) from the Late Cretaceous of Madagascar. J. Vertebr. Paleontol..

[110] Lillegraven JA, Krusat G (1991). Cranio-mandibular anatomy of *Haldanodon exspectatus* (Docodonta, Mammalia) from the Late Jurassic of Portugal and its implications to the evolution of mammalian characters. Contrib. Geol. Univ. Wyo..

[111] Botha-Brink J, Soares MB, Martinelli AG (2018). Osteohistology of Late Triassic prozostrodontian cynodonts from Brazil. PeerJ.

[112] Grossnickle DM, Smith SM, Wilson GP (2019). Untangling the multiple ecological radiations of early mammals. Trends Ecol. Evol..

[113] Rougier, G. W., Martinelli, A. G. & Forasiepi, A. M. *Mesozoic Mammals from South America and Their Forerunners*. (Springer, 2021) i-xvii + 388p.

[114] Martinelli AG, Eltink E, Da-Rosa ÁAS, Langer MC (2017). A new cynodont (Therapsida) from the *Hyperodapedon* Assemblage Zone (upper Carnian-Norian) of southern Brazil improves the Late Triassic probainognathian diversity. Pap. Palaeontol..

[115] Martinelli AG, Soares MB, Oliveira TV, Rodrigues PG, Schultz CL (2017). The Triassic eucynodont *Candelariodon barberenai* revisited and the early diversity of stem prozostrodontians. Acta Palaeont. Polon..

[116] Benoit J, Nxumalo M, Norton LA, Fernandez V, Gaetano LC, Rubidge B, Abdala F (2022). Synchrotron scanning sheds new light on *Lumkuia fuzzi* (Therapsida, Cynodontia) from the Middle Triassic of South Africa and its phylogenetic placement. J. Afr. Earth Sci..

[117] Romer AS (1969). The Chanares (Argentina) Triassic reptile fauna. V. A new chiniquodontid cynodont, *Probelesodon lewisi*—cynodont ancestry. Breviora.

[118] Abdala F, Giannini NP (2002). Chiniquodontid cynodonts: Systematic and morphometric considerations. Palaeontology.

[119] Bonaparte JF (2013). Evolution of the Brasilodontidae (Cynodontia-Eucynodontia). Hist. Biol..

[120] Bonaparte JF, Soares MB, Martinelli AG (2012). Discoveries in the Late Triassic of Brazil improve knowledge on the origin of mammals. Hist. Nat. Terc. Ser..

[121] Liu J, Olsen P (2010). The phylogenetic relationships of Eucynodontia (Amniota: Synapsida). J. Mammal. Evol..

[122] Martinelli AG, Bonaparte JF, Calvo J, Porfiri J, González-Riga B, dos Santos D (2011). Postcanine replacement in *Brasilodon* and *Brasilitherium* (Cynodontia, Probainognathia) and its bearing in cynodont evolution. Dinosaurios y Paleontología desde América Latina.

[123] Martinelli, A. G. Contribuição ao conhecimento dos cinodontes probainognátios (Therapsida, Cynodontia, Probainognathia) do Triássico da América do Sul e seu impacto na origem dos Mammaliaformes. Ph.D. thesis. Universidade Federal do Rio Grande do Sul, Brazil, 645 (2017).

[124] Martinelli, A. G., Gill, P. G., Corfe, I. J., Rodrigues, P. G., Fonseca, P. H., Schultz, C., Soares, M. B. & Rayfield, E. J. Ontogeny and tooth replacement in the brazilian cynodonts *Brasilodon quadrangularis*, *Brasilitherium riograndensis* and *Minicynodon maieri*. Reunión de Comunicaciones de la Asociación Paleontológica Argentina, La Plata, Libro Resúmenes, 62–63 (2019).

