# Supplementary material for: New evidence from high-resolution computed microtomography of Triassic stem-mammal skulls from South America enhances discussions on turbinates before the origin of Mammaliaformes
Source: Sci Rep. 2024 Jun 15;14:13817. doi: 10.1038/s41598-024-64434-5 (PMC11180108; doi:10.1038/s41598-024-64434-5)
Supplement: Supplementary file 1 — Supplementary Information 1. [file 41598_2024_64434_MOESM1_ESM.docx]

**Supplementary Data for:**

**New evidence from high-resolution computed microtomography of Triassic stem-mammal skulls from South America enhances discussions on turbinates before the origin of Mammaliaformes**

Pedro H. M. Fonseca*, Agustín G. Martinelli*, Pamela G. Gill*, Emily J. Rayfield*, Cesar L. Schultz, Leonardo Kerber, Ana Maria Ribeiro, Heitor Francischini, & Marina B. Soares*

**Correspondence and requests for materials should be addressed to P.H.M.F. ([phmorais.bio@gmail.com](mailto:phmorais.bio@gmail.com)), A.G.M. ([agustin_martinelli@yahoo.com.ar](mailto:agustin_martinelli@yahoo.com.ar)), P.G.G. (glpgg@bristol.ac.uk), E.J.R. ([e.rayfield@bristol.ac.uk](mailto:e.rayfield@bristol.ac.uk)) and M.B.S. ([marina.soares@mn.ufrj.br](mailto:marina.soares@mn.ufrj.br))

**List of content:**

1. CT Scanning data of the studied specimens.
2. Interpretative line drawings of the 3D models used in the figures 2, 4 and 5 of the main text.
3. Additional 3D imagens of the nasal cavity of *Riograndia guaibensis*.
4. Additional 3D imagens of the nasal cavity of *Brasilodon quadrangularis*.
5. **CT Scanning data of the specimens used.**

| **Collection Number** | **Taxon** | **Scanning Details** | **XraykV/µA** | **Voxel Size (mm)** |
| --- | --- | --- | --- | --- |
| NHMUK-PV-R8429 | *Chiniquodon theotonicus* | Natural History Museum, London, Zeiss Xradia Versa 520 |  | 0.063109 |
| NHMUK-PV-R511 | *Thrinaxodon liorhynnus* | Natural History Museum, London, Nikon Metrology HMX ST 225 |  | 0.015004 |
| UFRGS-PV-248-T | *Prozostrodon brasiliensis* | University of Bristol, Nikon XTH225 ST Micro CT | 190/36 | 0.021533 |
| UFRGS-PV-1043-T | *Brasilodon quadrangularis* | University of Bristol, Nikon XTH225 ST Micro CT | 150/47 | 0.012823 |
| UFRGS-PV-929-T | *Brasilodon quadrangularis* | University of Finland, Phoenix High-Resolution X-ray x\|s Nanofocus |  | 0.013333 |
| UFRGS-PV-1030-T | *Brasilodon quadrangularis* (formerly holotype *Minicynodon maieri*) | University of Bristol, Nikon XTH225 ST Micro CT | 180/39 | 0.008020 |
| UFRGS-PV-596-T | *Riograndia guaibensis* | University of Bristol, Nikon XTH225 ST Micro CT | 180/38 | 0.012324 |
| UFRGS-PV-788-T | *Riograndia guaibensis* | University of Bristol, Nikon XTH225 ST Micro CT | 155/171 | 0.02449 |
| UNISINOS-4881 | *Riograndia guaibensis* | University of Bristol, Nikon XTH225 ST Micro CT | 150/175 | 0.017428 |
| MCN-PV 2264 (holotype) | *Riograndia guaibensis* | Pontifícia Universidade Católica do Rio Grande do Sul, Bruker SkyScan 1173 | 80/100 | 0.01411 |
| UFRGS-PV-833-T | *Riograndia guaibensis* | University of Finland, Phoenix High-Resolution X-ray x\|s Nanofocus |  | 0.018333 |

1. **Interpretative line drawings of the 3D models used in the figures 2, 4 and 5 of the main text.**

**
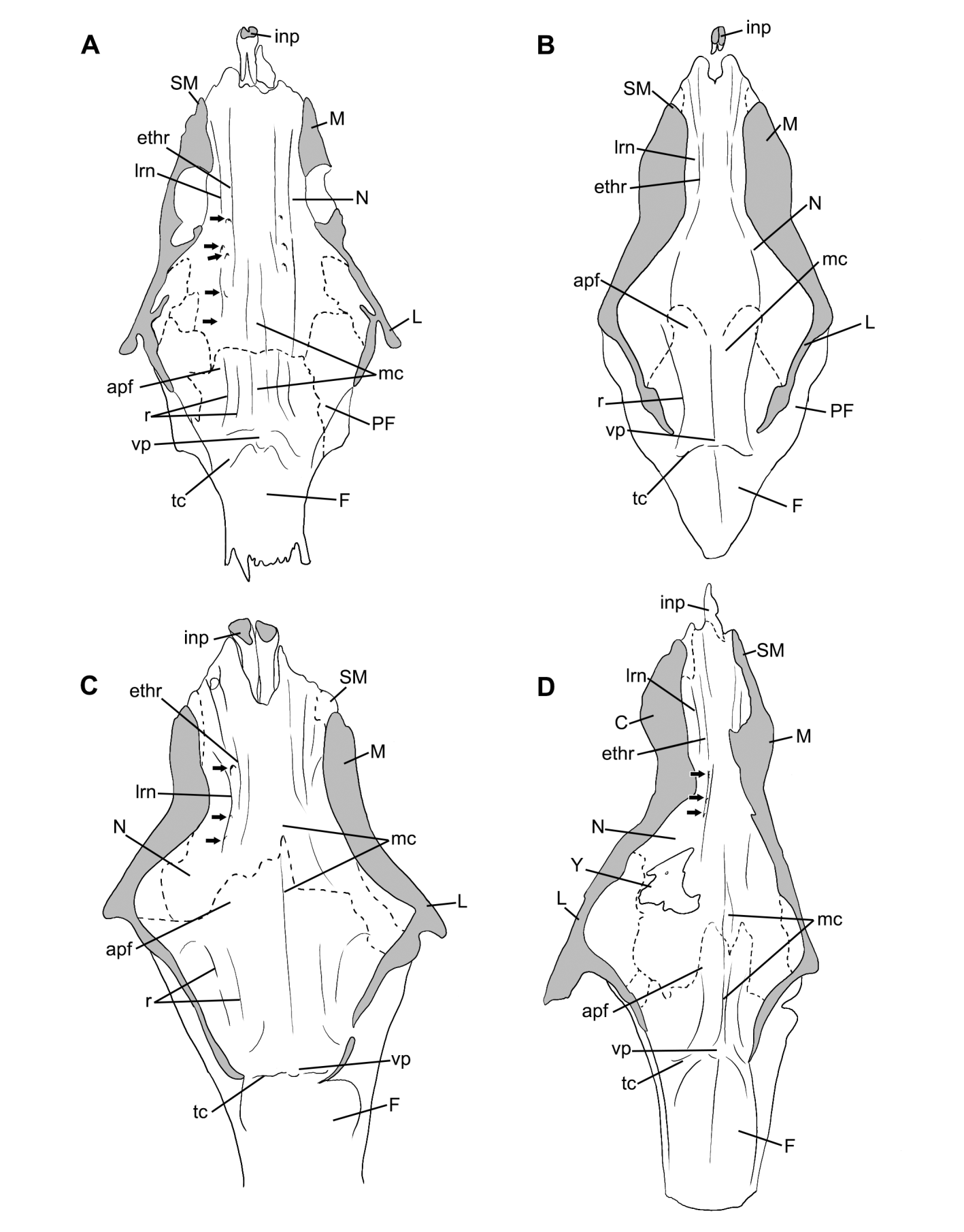
**

**Figure S1.** **Nasal cavities of the studied cynodonts.** **A-D**, Skull section in ventral (internal) view, exhibiting the ventral region of the roof of the nasal cavity, in *Thrinaxodon liorhinus* (NHMUK PV R511) (**A**), *Prozostrodon brasiliensis* (UFRGS-PV-248-T; the left half of skull is mirrored) (**B**), *Riograndia guaibensis* (UFRGS-PV-596-T) (**C**), and *Brasilodon* *quadrangularis* (UFRGS-PV-1043-T) (**D**). Black arrows indicate visible foramina. apf, anterior projection of the frontal; C, canine; F, frontal; ethr, ethmoid ridge of the nasal; inp, internarial process; L, lacrimal; lrn, lateral ridge of the nasal; M, maxilla; mc, median crest; N, nasal; PF, prefrontal; r, ridge; SM, Septomaxilla; tc, transverse crest; vp, ventral projection; Y, structure identified as a turbinal by Ruf *et al.* (2014).

**
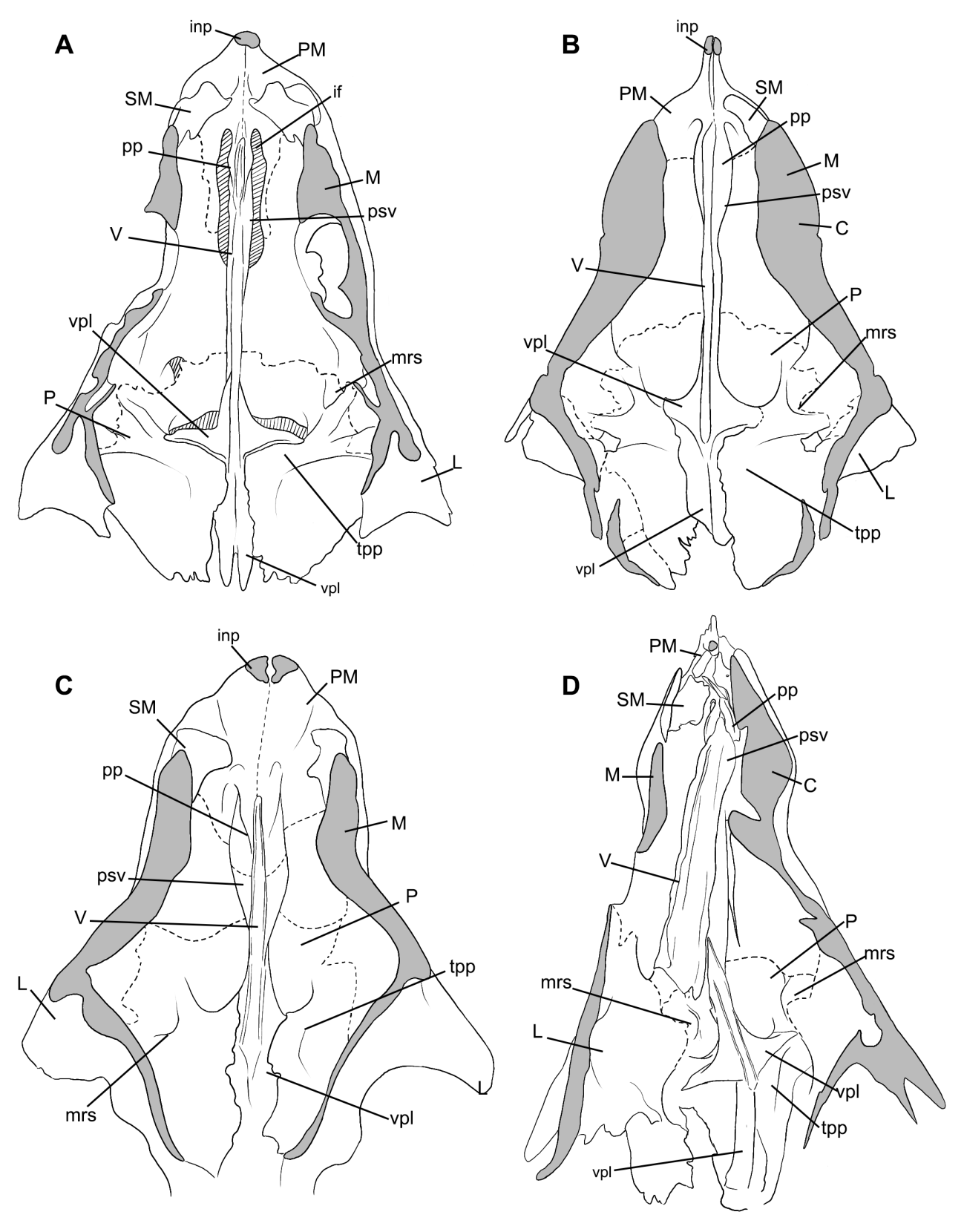
**

**Figure S2. Nasal cavities of the studied cynodonts. A-D,** Skull section in dorsal view, exhibiting the dorsal region of the floor of the nasal cavity in *Thrinaxodon liorhinus* (NHMUK PV R511) (**A**), *Prozostrodon brasiliensis* (UFRGS-PV-248-T, left half of the skull is mirrored) (**B**), *Riograndia guaibensis* (UFRGS-PV-596-T) (**C**), and *Brasilodon* *quadrangularis* (UFRGS-PV-1043-T) (**D**). C, canine; if, incisive foramen; inp, internarial process of premaxilla; L, lacrimal; M, maxilla; mrs, maxillary recess; P, palatine; PM, premaxilla; pp, palatine process of the premaxilla; psv, paraseptal shelf of the vomer; SM, septomaxilla; tpp, transverse process of the palatine; V, vomer; vpl, vomerine plate.


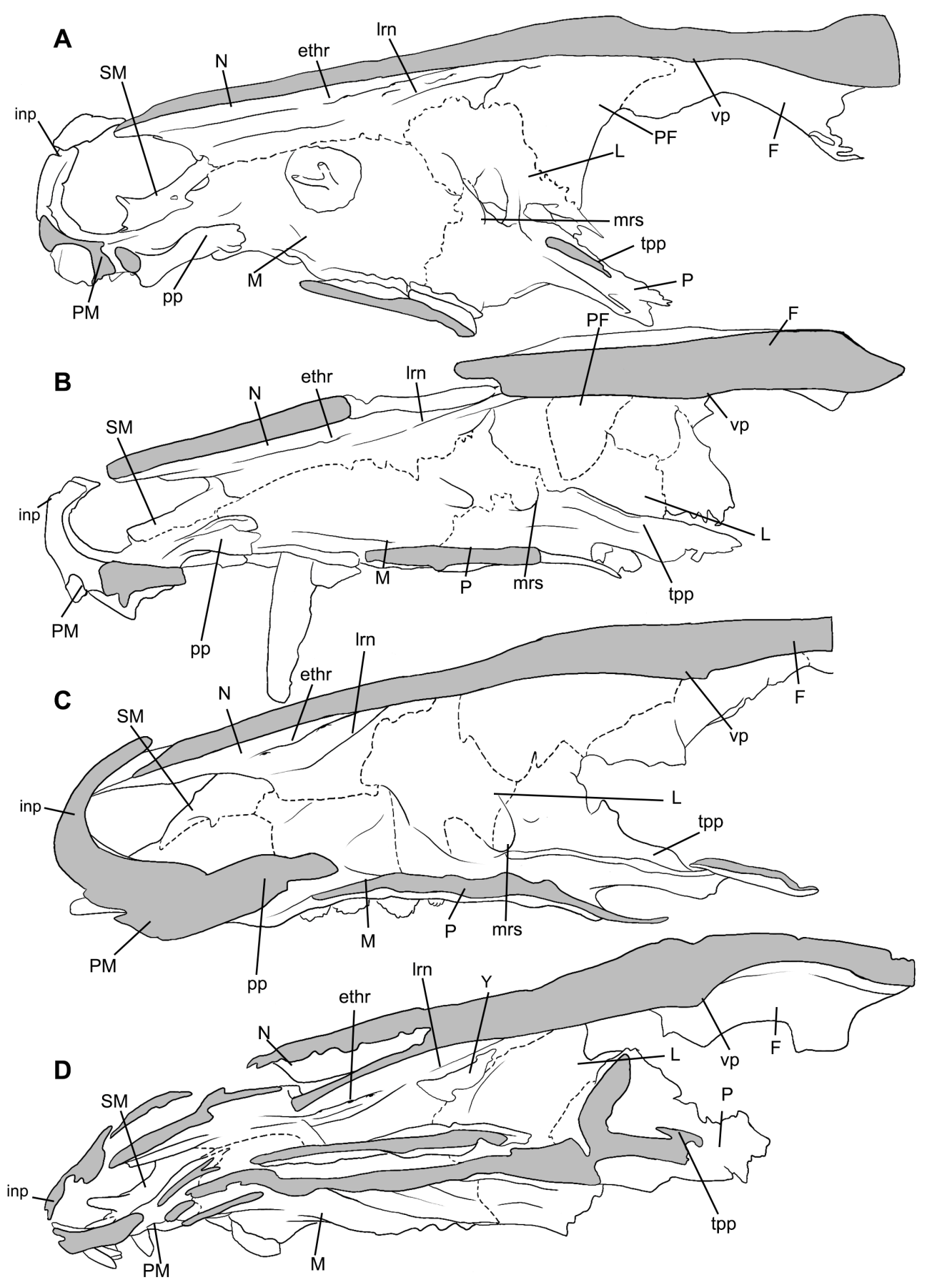


**Figure S3. Nasal cavities of the studied cynodonts.** **A-D**, Right half of the skull in lateral view, exhibiting the lateral wall of the nasal cavity, in *Thrinaxodon liorhinus* (NHMUK PV R511) (**A**), *Prozostrodon brasiliensis* (UFRGS-PV-248-T, left half of the skull is mirrored) (**B**), *Riograndia guaibensis* (UFRGS-PV-596-T) (**C**), and *Brasilodon* *quadrangularis* (UFRGS-PV-1043-T) (**D**). aold, anterior opening of lacrimal duct; apf, anterior projection of the frontal; ethr, ethmoid ridge of nasal; F, frontal; inp, internarial process of premaxilla; L, lacrimal; lrn, lateral ridge of nasal; M, maxilla; mrs, maxillar recess; N, nasal; P, palatine; PF, prefrontal; PM, premaxilla; pp, palatine process of the premaxilla; SM, septomaxilla; tpp, transverse process of the palatine; vp, ventral projection; Y, structure identified as a turbinal by Ruf *et al.* (2014).

1. **Additional 3D imagens of the nasal cavity of *Riograndia guaibensis*.**


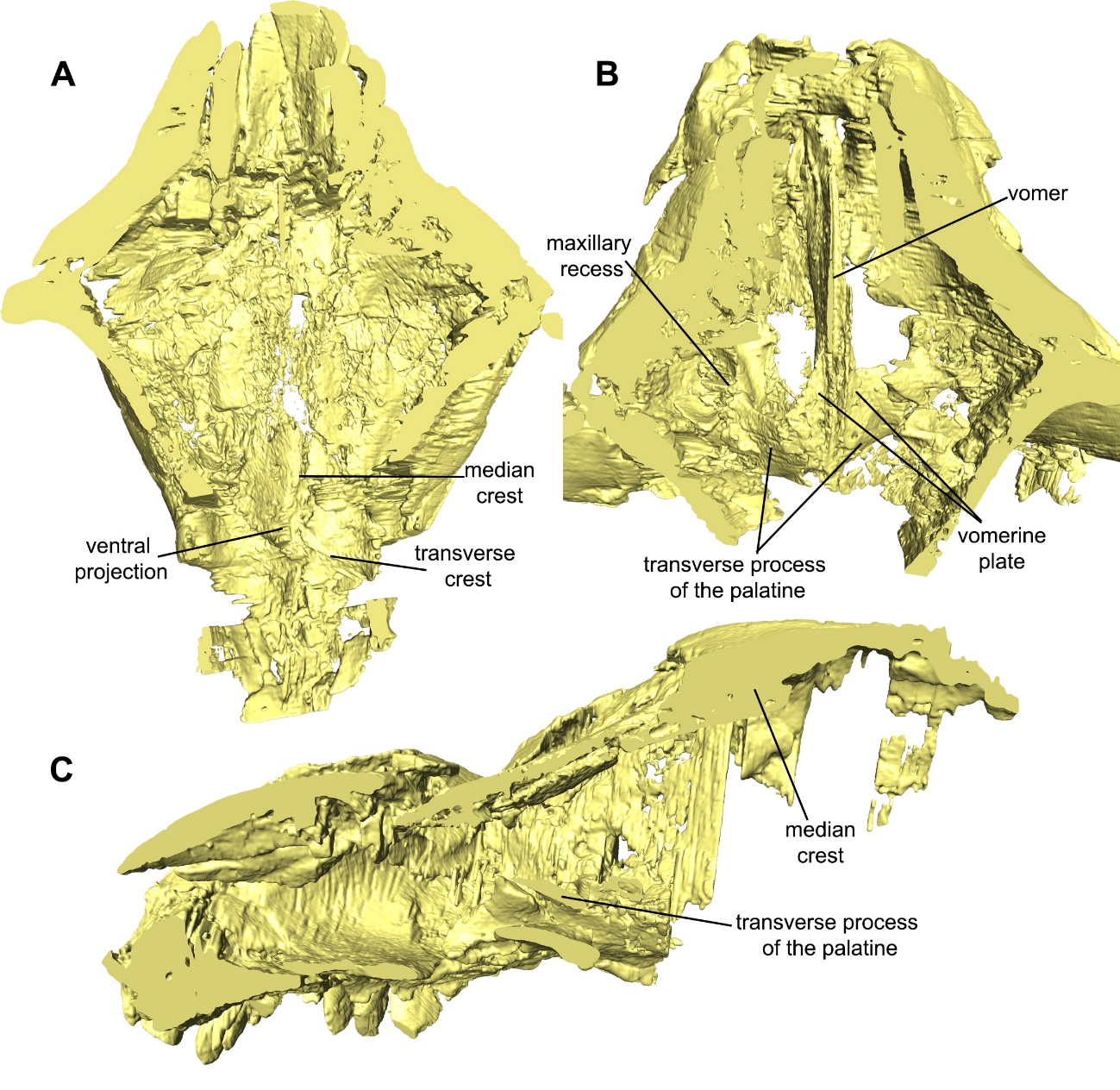


**Figure S4.** *Riograndia guaibensis*. 3D reconstruction of the nasal cavity of UFRGS-PV-833-T, showing the roof (A), the floor (B) and the right internal wall (C).


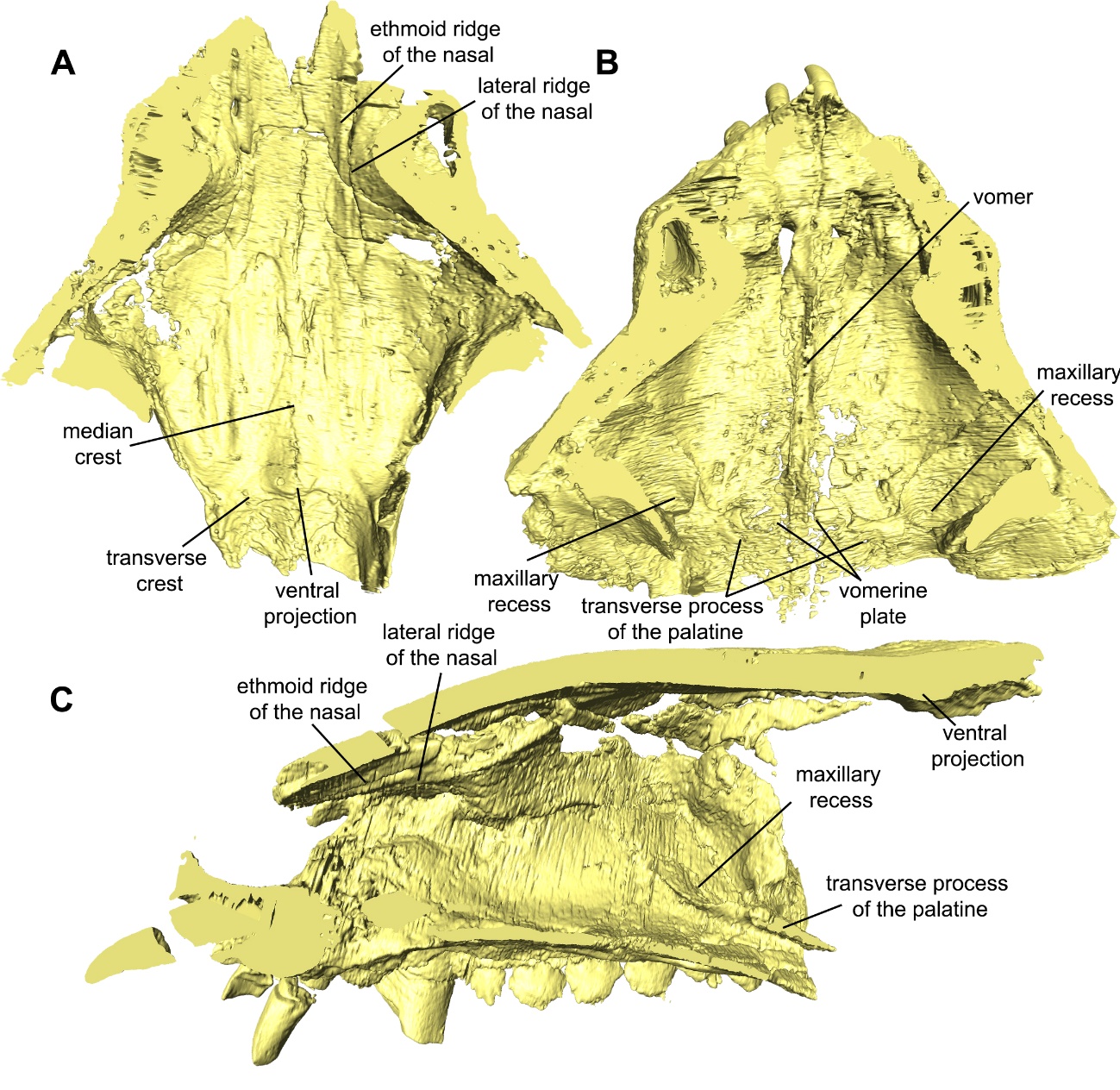


**Figure S5.** *Riograndia guaibensis*. 3D reconstruction of the nasal cavity of UNISINOS 4881, showing the roof (A), the floor (B) and the right internal wall (C).


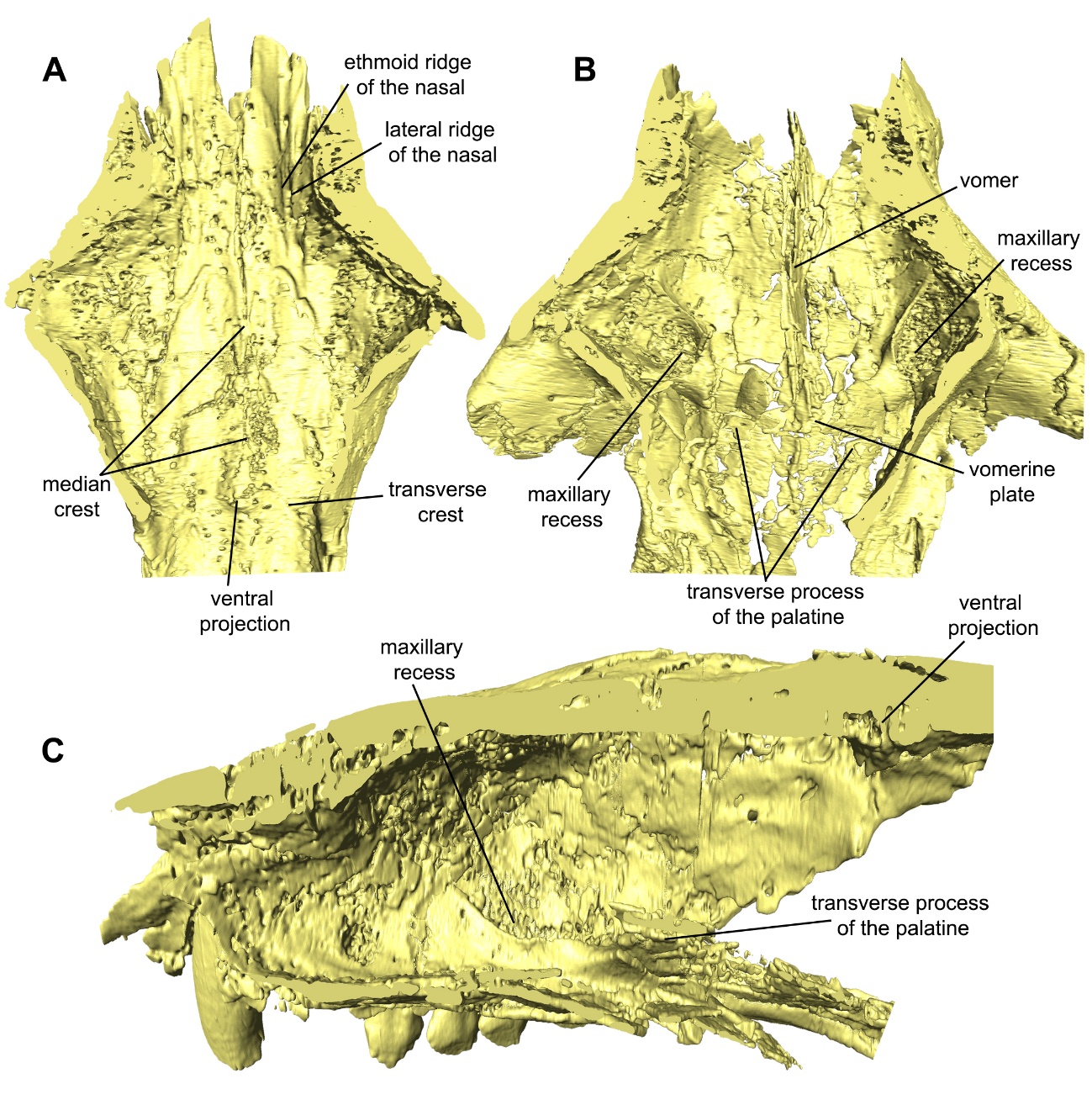


**Figure S6.** *Riograndia guaibensis*. 3D reconstruction of the nasal cavity of UFRGS-PV-788-T, showing the roof (A), the floor (B) and the right internal wall (C).

1. **Additional 3D imagens of the nasal cavity of *Brasilodon quadrangularis*.**


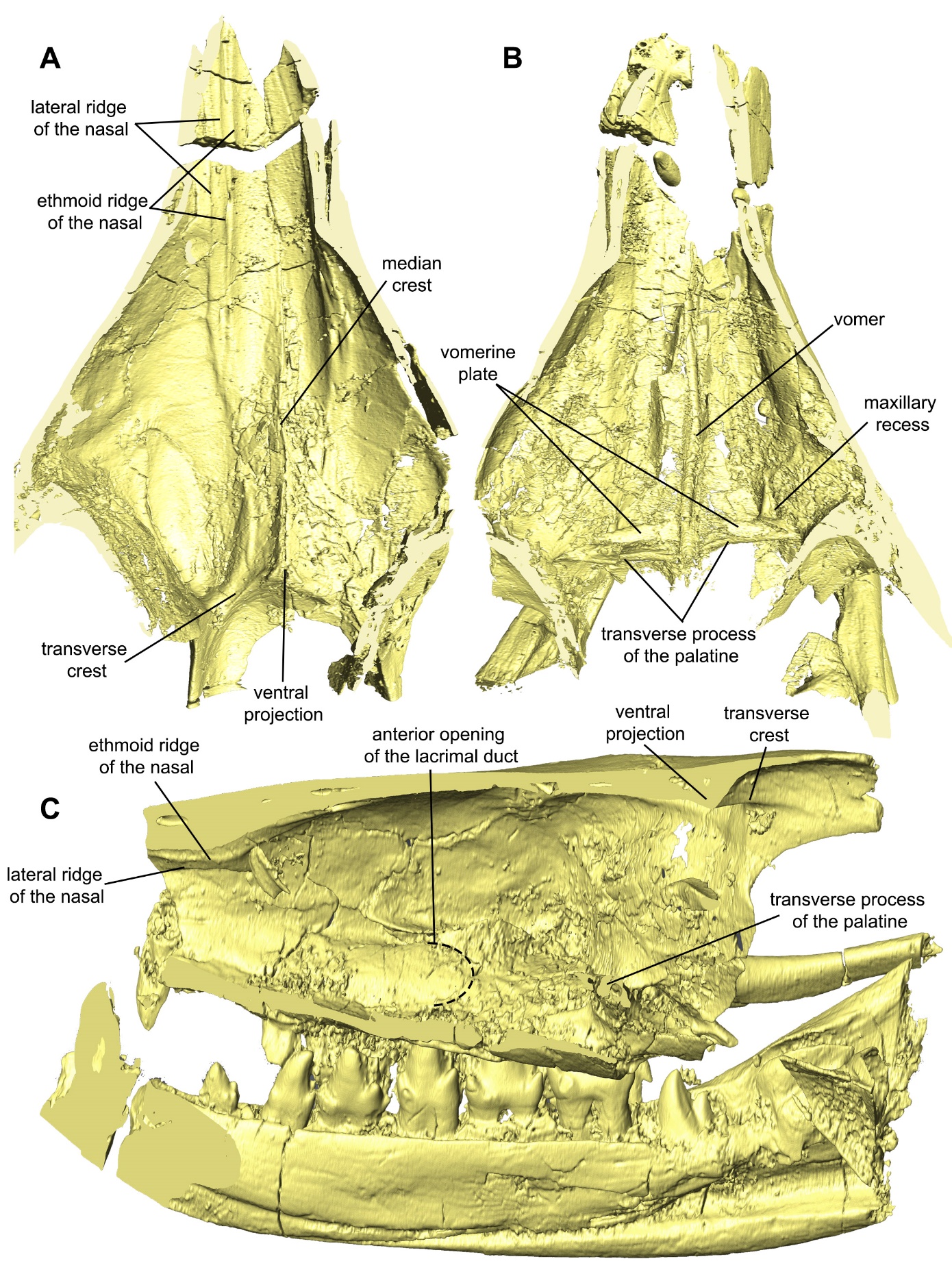


**Figure S7.** *Brasilodon quadrangularis*. 3D reconstruction of the nasal cavity of UFRGS-PV-1030-T, showing the roof (A), the floor (B) and the right internal wall (C), with the lower jaw in occlusion.

**Bibliography**

Ruf, I., Maier, W., Rodrigues, P. G., & Schultz, C. L. (2014). Nasal Anatomy of the non‐mammaliaform cynodont *Brasilitherium riograndensis* (Eucynodontia, Therapsida) reveals new insight into mammalian evolution. *Anat. Record*, **297**(11), 2018-2030
