# Supplementary material for: New evidence from high-resolution computed microtomography of Triassic stem-mammal skulls from South America enhances discussions on turbinates before the origin of Mammaliaformes
Source: Sci Rep. 2024 Jun 15;14:13817. doi: 10.1038/s41598-024-64434-5 (PMC11180108; doi:10.1038/s41598-024-64434-5)
Supplement: Supplementary file 2 — Supplementary Information 2. [file 41598_2024_64434_MOESM2_ESM.pptx]

## Slide 1
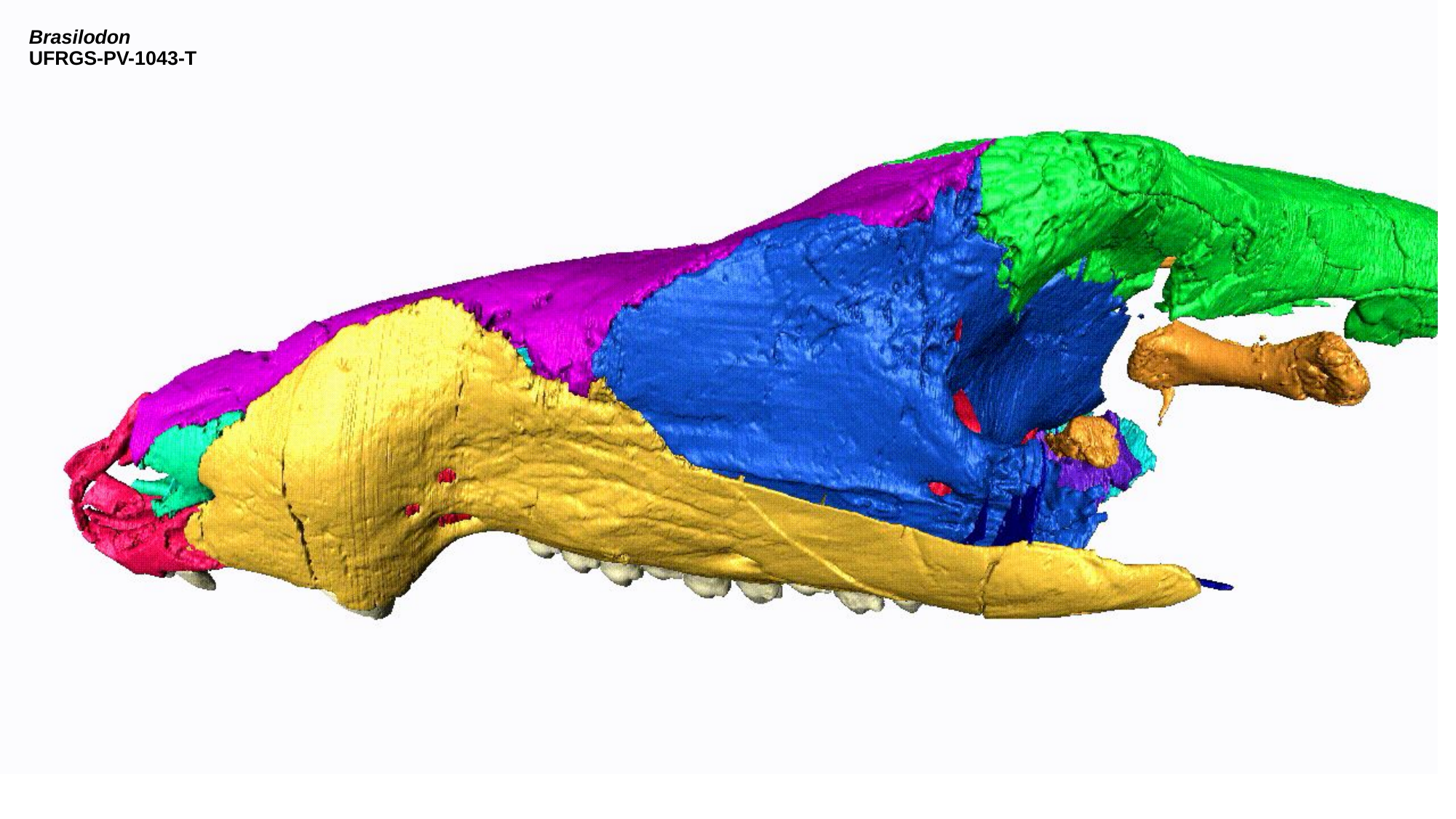

Brasilodon
UFRGS-PV-1043-T
#

## Slide 2
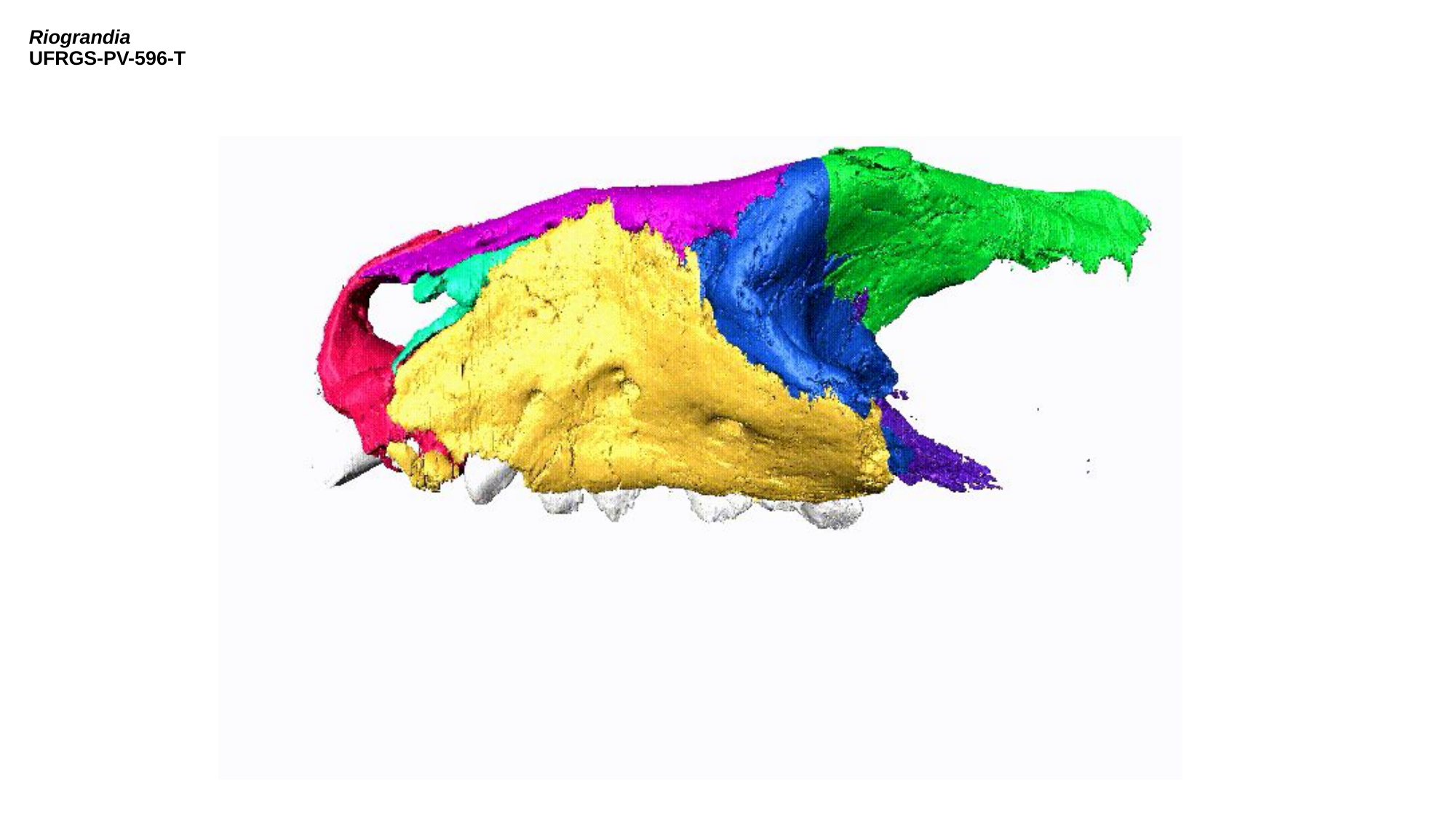

Riograndia
UFRGS-PV-596-T

## Slide 3
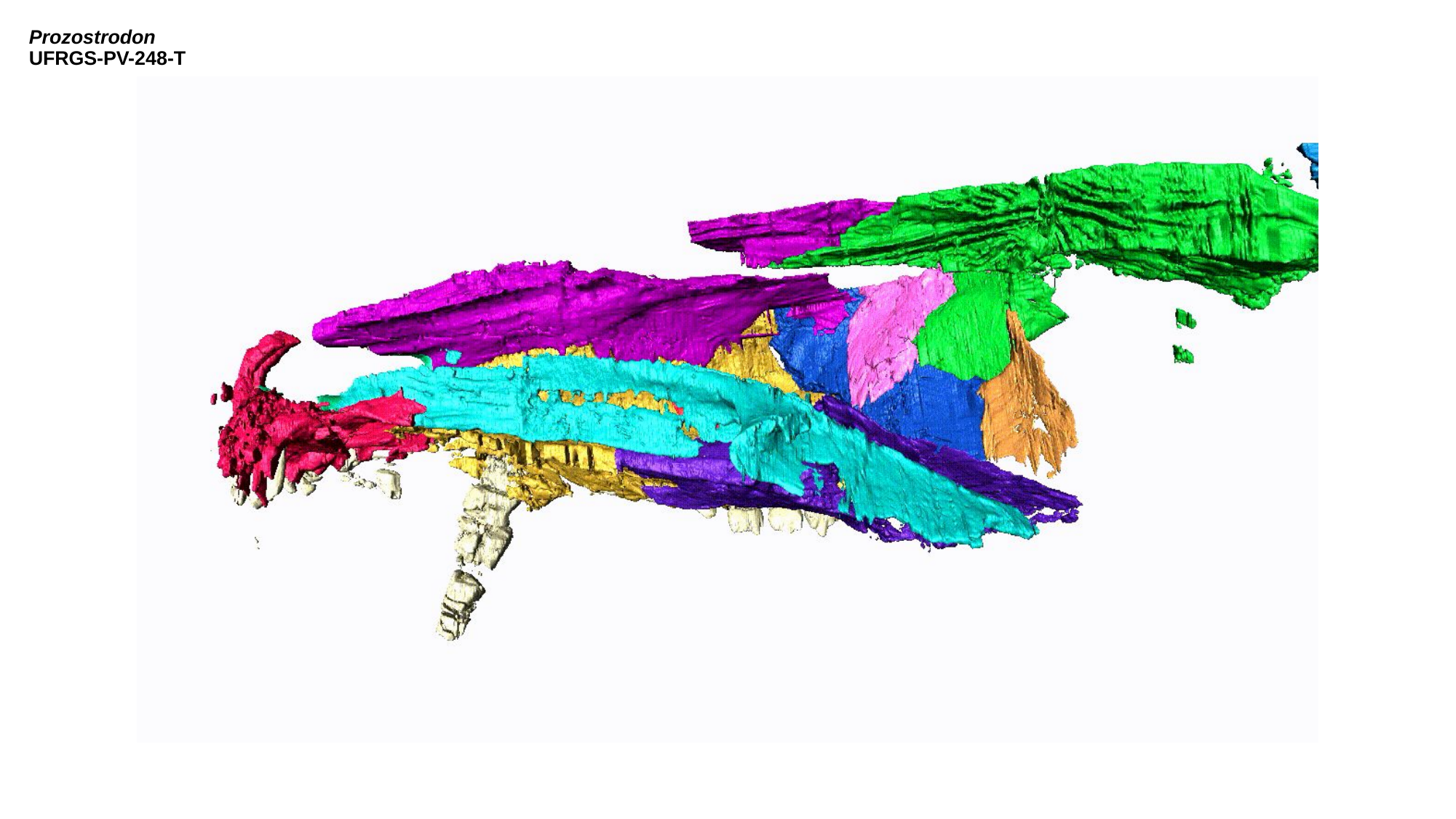

Prozostrodon
UFRGS-PV-248-T
#

## Slide 4
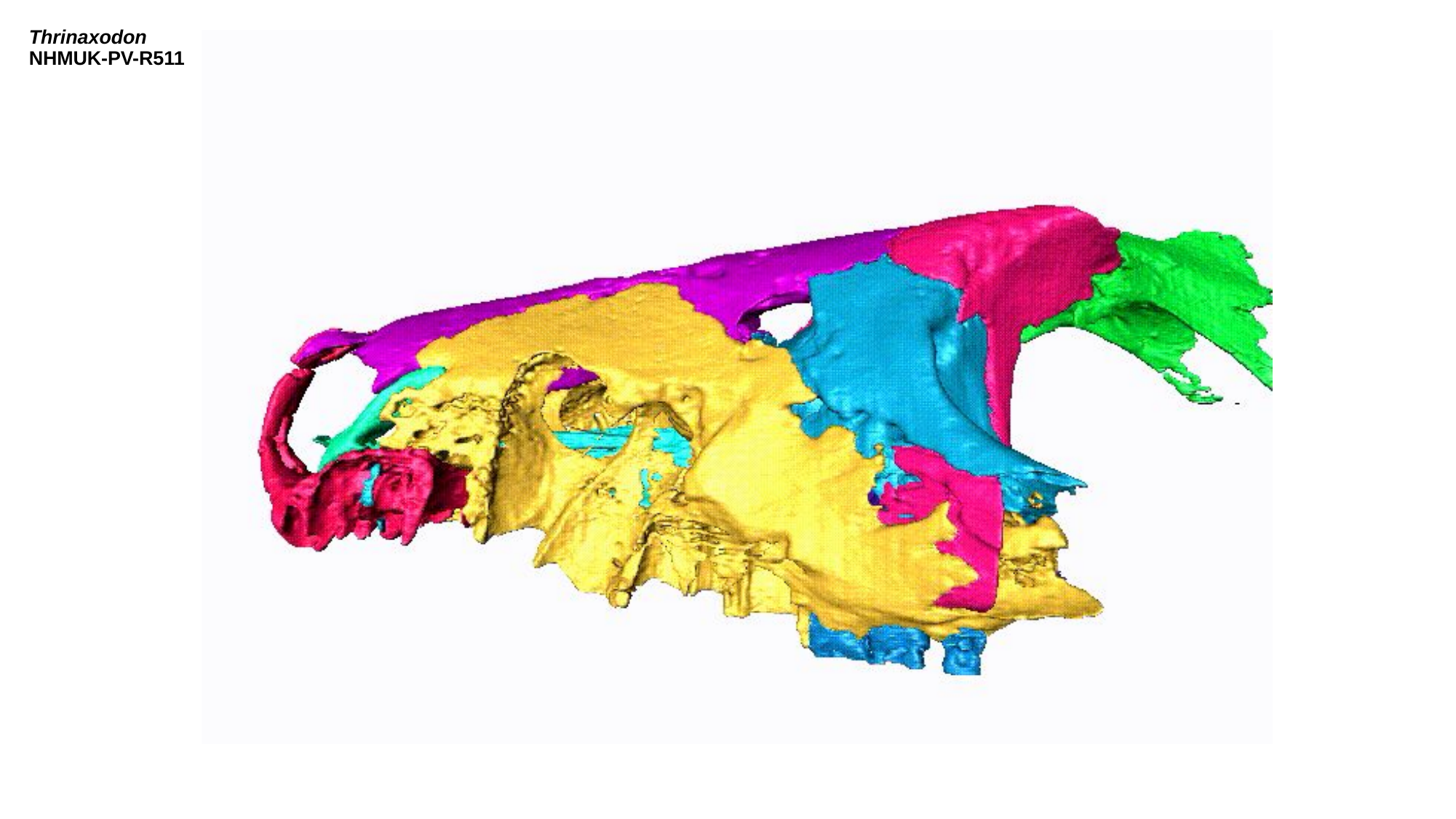

Thrinaxodon
NHMUK-PV-R511
